# Construction and validation of a novel cuproptosis-related long noncoding RNA signature for predicting the outcome of prostate cancer

**DOI:** 10.3389/fgene.2022.976850

**Published:** 2022-12-06

**Authors:** Shaoqin Jiang, Zhihao Li, Ruiling Dou, Zequn Lin, Jili Zhang, Wenhui Zhang, Zeyu Chen, Xianqi Shen, Jin Ji, Min Qu, Yan Wang, Mengqiang Li, Xu Gao

**Affiliations:** ^1^ Department of Urology, Changhai Hospital, Second Military Medical University, Shanghai, China; ^2^ Department of Urology, Fujian Union Hospital, Fujian Medical University, Fuzhou, Fujian, China

**Keywords:** cuproptosis, prostate cancer, long noncoding RNA signature, prognosis model, nomogram

## Abstract

**Background:** Prostate cancer (PCa) is one of the most common tumors of the urinary system. Cuproptosis is a novel mode of controlled cell death that is related to the development of various tumor types. However, the functions of cuproptosis-related long noncoding RNAs (CRLs) in PCa have not yet been well studied.

**Methods:** In this study, data of PCa patients were obtained from The Cancer Genome Atlas (TCGA) and from the Changhai Hospital. Univariate and multivariate Cox regression analyses and LASSO regression analysis were conducted to screen CRLs linked to the prognosis of PCa patients. A risk score model was constructed on the basis of CRLs to predict prognosis. PCa patients were categorized into high- and low-risk cohorts. The predictive value of the risk score was evaluated by Kaplan–Meier survival analysis, receiver operating characteristic curves, and nomograms. In addition, gene ontology (GO) and Kyoto Encyclopedia of Genes and Genomes (KEGG) enrichment analyses were used to explore possible pathways involving CRLs in PCa. Immune function analysis confirmed the correlation between CRLs and immunity in PCa. Finally, we explored the tumor mutational burden and drug response in the high- and low-risk cohorts.

**Results:** First, we identified seven CRLs (C1orf229, C9orf139, LIPE-AS1, MCPH1-AS1, PRR26, SGMS1-AS1, and SNHG1) that were closely related to prognosis in PCa. The risk score model based on the selected CRLs could accurately predict the prognosis of PCa patients. The high-risk cohort was associated with worse disease-free survival (DFS) time in PCa patients (*p* < 0.001). ROC curve analysis was performed to confirm the validity of the signature (area under the curve (AUC) at 1 year: 0.703). Nomograms were constructed based on the risk score and clinicopathological features and also exhibited great predictive efficiency for PCa. GO and KEGG analyses showed that the CRLs were mainly enriched in metabolism-related biological pathways in PCa. In addition, immune function analysis showed that patients in the high-risk cohort had higher TMB and were more sensitive to conventional chemotherapy and targeted drugs including doxorubicin, epothilone B, etoposide, and mitomycin C.

**Conclusion:** We constructed a novel CRL-related risk score model to accurately predict the prognosis of PCa patients. Our results indicate that CRLs are potential targets for drug therapy in PCa and provide a possible new direction for personalized treatment of PCa patients.

## Introduction

Prostate cancer (PCa) is the second most common malignant tumor and the sixth most common cause of cancer-related deaths in men worldwide (Culp et al., 2020). Early screening for PCa is not common, especially in Asia, and most patients have locally advanced or metastatic PCa at the time of diagnosis. Androgen-deprivation therapy (ADT) is the main treatment for advanced PCa. However, long-term ADT treatment can induce a castration-resistant state. Castration-resistant PCa (CRPC) is associated with a higher risk of distant metastasis and worse disease-free survival (DFS) (Moreira et al., 2017). Proper diagnosis and management contribute greatly to preventing development of CRPC. Therefore, it is of great importance to find new biomarkers for the prognosis of PCa.

Cuproptosis is a newly discovered form of cell death that is different from pyroptosis, ferroptosis, etc. Cuproptosis blocks the tricarboxylic acid (TCA) cycle by the accumulation of intracellular copper, which leads to protein toxic stress and induces cell death (Tsvetkov et al., 2022). Excessive accumulation of glycolytic intermediates and enrichment of genes involved in the TCA cycle have significant roles in the development of PCa (Costello et al., 2005; Shao et al., 2018; Singh and Mills, 2021). The above biological processes induce overactivation of the TCA cycle to facilitate proliferation of PCa. This suggests that cuproptosis may be related to the progression of PCa. Cuproptosis-related genes could also serve as novel biomarkers to predict prognosis of PCa patients.

Long noncoding RNAs (lncRNAs) are noncoding RNAs with a length of more than 200 nucleotides. Abundant studies have shown that lncRNAs have key roles in tumor proliferation, migration, and programmed cell death (Wen et al., 2020; Zhang et al., 2020; Tan et al., 2021). For example, lncRNA MNX1-AS1 regulates the migration and invasion of PCa cells by targeting miR-2113 (Liang et al., 2022). Overexpression of RP1-59D14.5 could activate the Hippo signaling pathway to inhibit the proliferation, migration, and invasion of PCa (Zhong et al., 2022). Linc00963 promotes proliferation, metastasis, invasion, and epithelial–mesenchymal transition of PCa via regulating the miR-542-3p/NOP2 axis (Sun et al., 2020). This lncRNA can also upregulate the expression of TRIM24 to promote the proliferation of CRPC (Bai et al., 2021). A study indicated that silencing Linc01963 led to downregulation of levels of TrkB, which improved the sensitivity of docetaxel-resistant PCa to chemotherapy (Xing et al., 2022). To date, there has been no study of the function of cuproptosis-related lncRNAs (CRLs) in PCa; this requires further investigation.

In this study, we constructed a risk predictive model based on CRLs in PCa. The risk score was compared with other clinicopathological variables to assess the prognostic efficacy of the model. We also analyzed the mechanism of CRLs in PCa by performing gene ontology (GO) and Kyoto Encyclopedia of Genes and Genomes (KEGG) analyses, immune-related function analysis, and drug sensitivity analysis. The purpose of our study was to confirm the predictive value of CRLs in the prognosis of PCa and demonstrate that they may have a role in the development of new therapeutic strategies for PCa.

## Materials and methods

### Patients and datasets

This study included two independent PCa patient cohorts, from The Cancer Genome Atlas (TCGA) and Changhai Hospital, respectively. Transcriptome data (FPKM values) from TCGA-PCa were downloaded from the TCGA official website (https://portal.gdc.cancer.gov/) and normalized. The corresponding clinical information of 490 PCa patients was also obtained from TCGA. The download of TCGA data was completed on 20 May 2022. To facilitate study of the differences in gene expression and other bioinformation of PCa tissues between Chinese and Western populations, our team developed a genomic and epigenomic atlas of PCa in Asian populations (Li et al., 2020). We extracted complete FPKM-standardized RNA sequencing (RNA-seq) data for 136 PCa patients from Changhai Hospital in the previous study. DFS data for PCa patients in this dataset were based on follow-up until 15 March 2022. The transcriptome data of the two cohorts were combined via log2 normalization and removal of batch effects with the “combat” R package. A total of 19 cuproptosis-related genes were obtained from a previous study (NFE2L2, NLRP3, ATP7B, ATP7A, SLC31A1, FDX1, LIAS, LIPT1, LIPT2, DLD, DLAT, PDHA1, PDHB, MTF1, GLS, CDKN2A, DBT, GCSH, and DLST) (Kahlson and Dixon, 2022).

Gene mutation data for PCa were downloaded from the TCGA official website (https://portal.gdc.cancer.gov/, accessed on 20 May 2022). We selected “Simple Nucleus Variation” as the data category, “Masked Somatic Mutation” as the data type, and “mar” as the form to save the downloaded file.

### Construction of the CRL predictive model

A PCa risk score model was established on the basis of RNA-seq data from the TCGA cohort and the Changhai Hospital cohort, which included 19 cuproptosis-related genes. The “limma” R package was used to calculate the correlations between expression of cuproptosis-related genes and lncRNAs. A total of 173 CRLs were extracted using the criteria |R2| ≥ 0.4 and *p* < 0.05. We randomly divided the PCa patients into a training set and a test set. In the training set, univariate Cox regression analysis was used to screen out the CRLs (*p* < 0.05) that were associated with DFS of PCa patients. To avoid overfitting of the model, we performed LASSO regression analysis with the “glmnet” R package and further screened out CRLs in PCa. Multivariate Cox regression analysis was performed and identified seven CRLs (C1orf229, C9orf139, LIPE-AS1, MCPH1-AS1, PRR26, SGMS1-AS1, and SNHG1) that were used to construct a risk score model for PCa. The risk scores of PCa patients were calculated by the following formula:

$$RISK\;Scores=\sum \left(expression\left(lncRNAs\right)*\beta \right)$$

Using the newly constructed PCa prognostic risk score formula, PCa patients in the training set and the test set were categorized into high- and low-risk cohorts. The “survival” and “survminer” R packages were used for Kaplan–Meier survival analysis to compare the DFS of PCa patients in the high- and low-risk cohorts. The “timeROC” package was used to evaluate the accuracy of the novel risk score model in predicting the DFS of PCa patients. The prediction efficiency of the new model was confirmed in the test set.

To evaluate the applicability of the CRL risk score model, we divided different subgroups according to patients’ age, Gleason score (GS), pathological T stage, pathological N stage, and surgical margin (SM). Kaplan–Meier survival analysis was used to evaluate the predictive power of the new model in subgroups with different clinical characteristics.

To better evaluate prognosis in PCa, we combined the cuproptosis-related risk score model with patient clinicopathological characteristics (age, GS, T stage, N stage, and SM). The “rms,” “regplot,” and “survival” R packages were used to build nomograms that could predict the 1-, 3-, and 5-year DFS of PCa patients. We also constructed a calibration curve to test the accuracy and reliability of the nomograms.

### Functional enrichment analysis of CRL predictors

The risk score model was used to classify PCa patients into high- and low-risk cohorts. Cuproptosis-related genes with differential expression between the high- and low-risk cohort were screened out using the “limma” R package (|log2 fold change| ≥ 0.585, and false discovery rate <0.05). We further performed GO and the KEGG analyses to annotate gene functions of the differentially expressed genes (DEGs) using the “clusterProfiler” R package. Then, the “GSVA” R package was used to evaluate the activities of 13 immune-related pathways involving DEGs in the high- and low-risk cohorts.

On 20 May 2022, we downloaded PCa somatic mutation data through the full exome sequencing platform available through the official TCGA website. The downloaded data format was mutation annotation format (MAF). The R package “maftools” was used to screen the gene mutation frequencies of patients in the high- and low-risk cohorts, and the mutation sites and the highest mutation frequencies of the 15 genes in PCa were visualized. The “limma” package was used to compare the degree of tumor mutation burden (TMB) between the high- and low-risk cohorts. The survival and prognosis of PCa patients with different degrees of TMB were analyzed using the “survminer” R package.

### Prediction of potential therapeutic drug response in prostate cancer

To evaluate the ability of the CRL risk score model to predict drug response in PCa, we used the “pRRophetic0.5″ R package to calculate the half-maximal inhibitory concentration (IC50) of certain conventional clinical drugs for the treatment of PCa. Then, the “limma” R package was used to analyze the differences in IC50 values between the high- and low-risk cohorts to guide clinical decision-making.

### Statistical analysis

All data were sorted and analyzed using R software (version 4.1.1) and Perl 5 (version 5.30.0). Univariate Cox regression was used to analyze the relationships between CRLs and DFS of PCa patients. LASSO regression was used to avoid overfitting of the model and obtain a model with optimized variables. Finally, multivariate Cox analysis was used to screen out CRLs that could be used to construct a risk score model to predict the progression of PCa. ROC curve analysis was used to evaluate the predictive accuracy of the DFS prognostic model. The risk score was combined with clinical characteristics to construct a nomogram to predict the DFS of PCa. *p* < 0.05 was considered the threshold for statistical significance; all *p*-values were two-tailed.

## Results

### Identification of CRLs in prostate cancer

**FIGURE 1:**
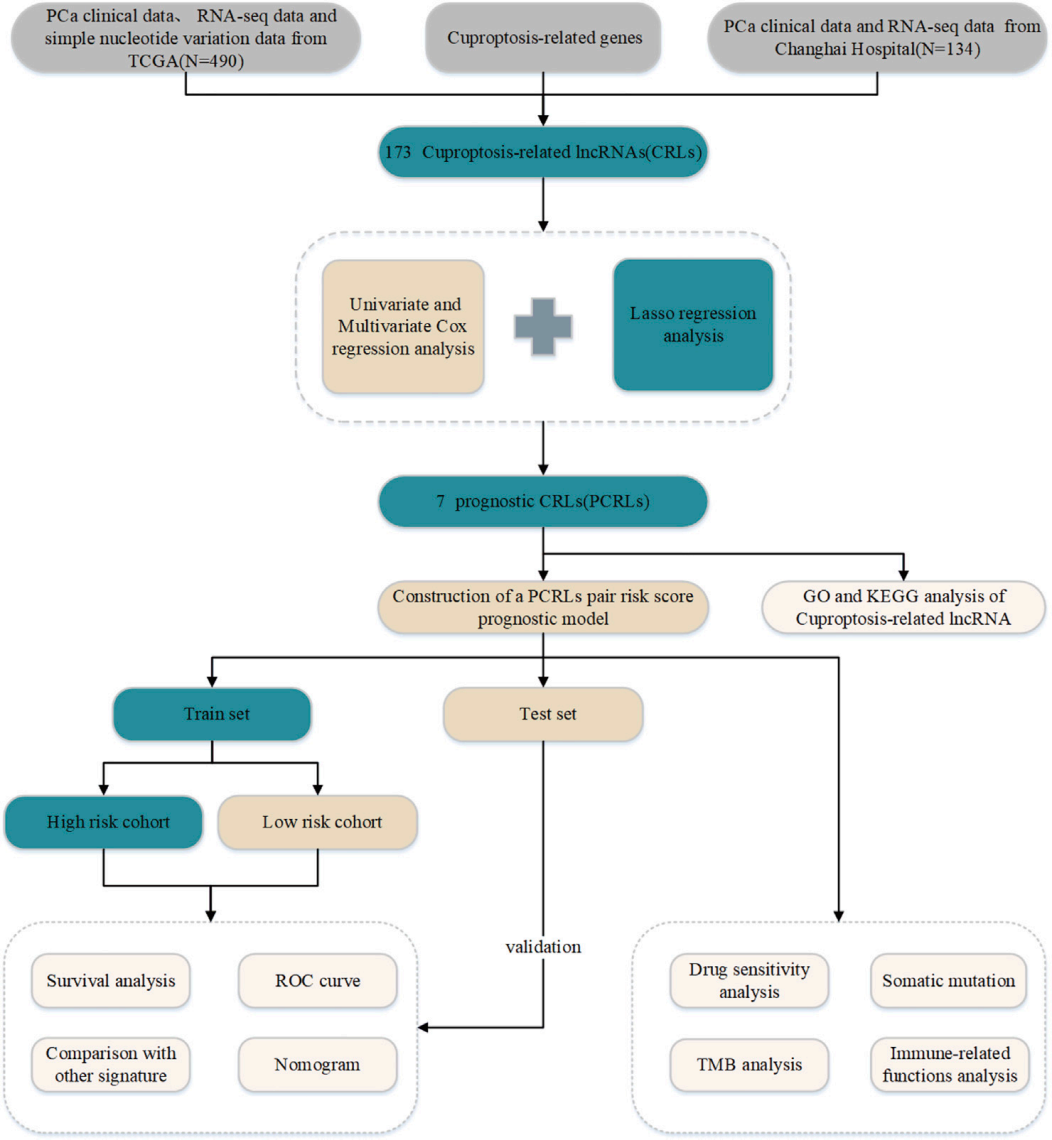
Flow chart of the study. TCGA: The Cancer Genome Atlas; PCa: prostate cancer; lncRNAs: long noncoding RNAs. GO: gene ontology; KEGG: Kyoto Encyclopedia of Genes and Genomes.

**FIGURE 2:**
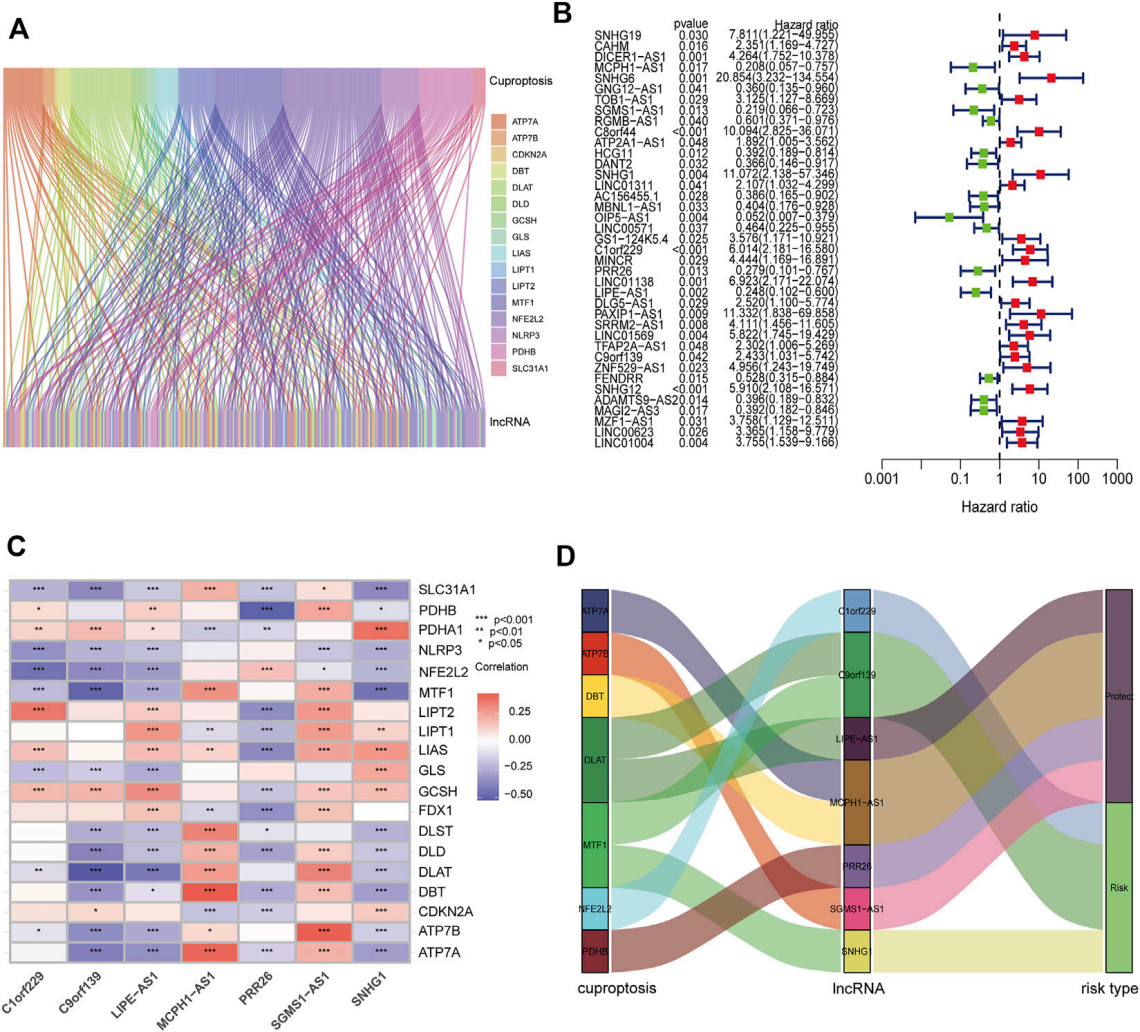
Construction of prognostic CRL signature in PCa. **(A)** Sankey diagram showing the relationships between 173 CRLs and cuproptosis-related genes. **(B)** Forest plot of 39 prognostic CRLs selected by univariate Cox regression analysis. **(C)** Heatmap of the associations between the expression levels of the seven CRLs and cuproptosis-related genes. **(D)** Sankey diagram showing the correlations of seven CRLs, cuproptosis-related genes, and risk types.

A flowchart of this study is shown in Figure 1. First, we obtained a list of 19 cuproptosis-related genes (Tsvetkov et al., 2022). The RNA-seq dataset for the above genes was obtained from the TCGA and Changhai Hospital data. Finally, we obtained 173 CRLs (Figure 2A).

### Construction of the CRL risk score model in prostate cancer

To verify the prognostic potential of CRLs in PCa, the TCGA and Changhai Hospital dataset was randomly categorized into a training set and test set. The clinical characteristics of the samples in the two sets are shown in Supplementary Table S1. A total of 39 CRLs related to the prognosis of PCa were preliminarily identified by univariate Cox regression analysis performed on the training set (Figure 2B). Then, LASSO regression analysis was used to filter 17 of the 39 CRLs related to the prognosis of PCa. Supplementary Figures S1A,B show the cvfit and lambda curves, respectively. Further, multivariate Cox regression was used to screen seven CRLs that were used to build a prognostic risk score model. The correlations between the expression of these seven CRLs and cuproptosis-related genes are shown in Figure 2C. A Sankey diagram was used to visualize the co-expression relationships between these seven CRLs and cuproptosis-related genes. The results indicated that C1orf229, C9orf139, and SNHG1 were risk factors, whereas LIPE-AS1, MCPH1-AS1, PRR26, and SGMS1-AS1 were protective factors for PCa patients (Figure 2D). The CRL risk score was calculated as follows: risk score = MCPH1-AS1*(−1.53858002747392)–SGMS1-AS1*0.905123102552302 + SNHG1*1.50996720923543 + C1orf229*1.41525455919748–PRR26*1.05076026105109–LIPE-AS1* 1.29480669719969 + C9orf139*0.783456595077748.

### Validation of the CRL risk score model

**FIGURE 3:**
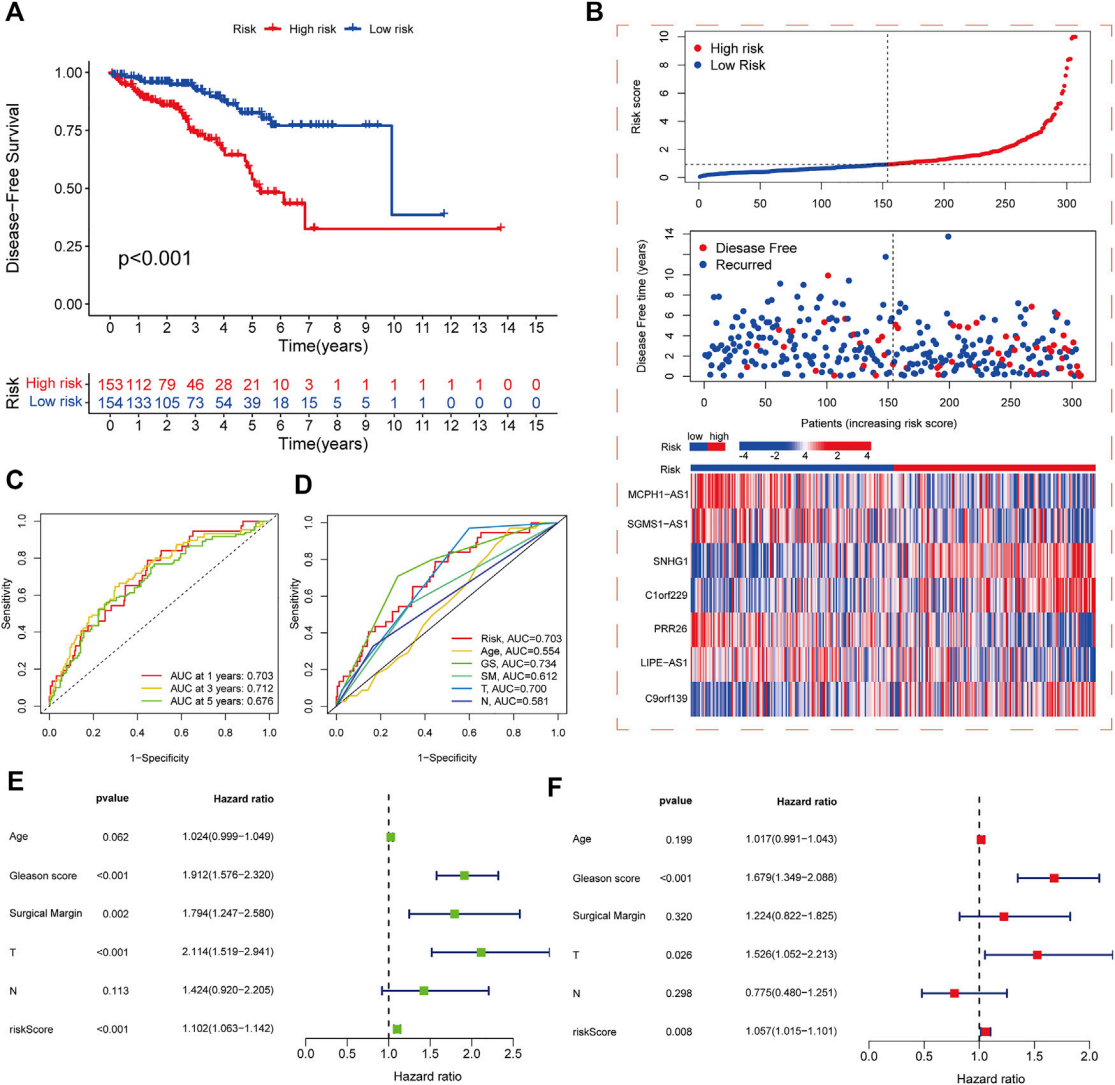
Development and validation of risk score model. **(A)** Kaplan-Meier analysis of DFS rates in high- and low-risk cohorts in the training set. **(B)** Risk score distribution, survival distribution, and heatmap of CRL expression for patients in high- and low-risk cohorts in the training set. **(C)** ROC curves for 1-, 3-, and 5-year DFS based on the risk score model in the training set. **(D)** ROC curves for risk score and other clinicopathological characteristics based on DFS in PCa patients. **(E)** Forest plot of the associations between risk score and other clinicopathological characteristics by univariate Cox regression analysis. **(F)** Forest plot of the associations between the risk score and other clinicopathological characteristics by multivariate Cox regression analysis.

The samples in the training and test sets were divided into high- and low-risk cohorts according to the median risk score. PCa patients in the high-risk cohort in the training set had significantly worse DFS than those in the low-risk cohort (*p* < 0.001) (Figure 3A), and the distribution of risk scores and survival was displayed in the training set (Figure 3B). The sample distribution was also reasonable for the high- and low-risk cohorts in all sets (Supplementary Figures S2A,B) and the test set (Supplementary Figures S2C,D). The 1-, 3-, and 5-year DFS area under the curve values were 0.703, 0.712, and 0.676 respectively, indicating good predictive performance (Figure 3C). Based on the ROC curves constructed from risk scores and clinicopathological parameters, pathological T stage, GS, and risk score showed better predictive efficacy than other clinicopathological parameters (Figure 3D). Both univariate and multivariate Cox regression analyses showed that pathological T stage, GS, and risk score were independent predictors of DFS in PCa patients (Figures 3E,F).

**FIGURE 4:**
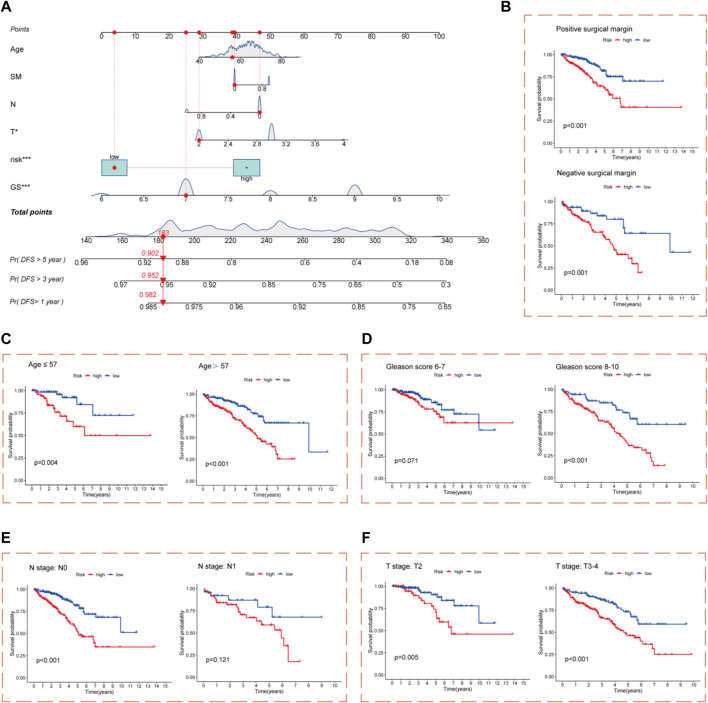
Construction of nomograms. **(A)** Nomograms composed of clinicopathological characteristics and risk score to predict 1-, 3-, and 5-year DFS of PCa patients; Kaplan–Meier survival curves for the high- and low-risk cohort based on clinicopathological variables are shown **(B)** Positive and negative SM subgroups. **(C)** Age (57 years and ≤57 years) subgroups. **(D)** GS (6–7 and 8–10) subgroups. **(E)** Pathological N0 and N1 stage subgroups. **(F)** Pathological T2 and T3–4 stage subgroups.

Nomograms incorporating clinicopathological features and risk score were constructed to improve the clinical applications of the CRL risk score model for PCa. The nomograms predicted DFS outcomes at 1, 3, and 5 years for PCa patients (Figure 4A). The calibration curve showed good consistency between actual DFS and the predicted 1-, 3-, and 5-year DFS values (Supplementary Figure S3). Survival analysis after grouping patients in high- and low-risk cohorts by clinicopathological parameters showed that for the age, GS, pathological T stage, and SM subgroups, DFS was significantly worse in the high-risk cohort compared with the low-risk cohort (Figures 4B–D,F). In the pathological N stage subgroup, only pathological N0 stage showed a significant difference between the high- and low-risk cohorts (Figure 4E). These results demonstrate that the risk score model maintains its predictive efficacy in most subgroups.

### GO and Kyoto Encyclopedia of Genes and Genomes enrichment analysis of CRLs

**FIGURE 5:**
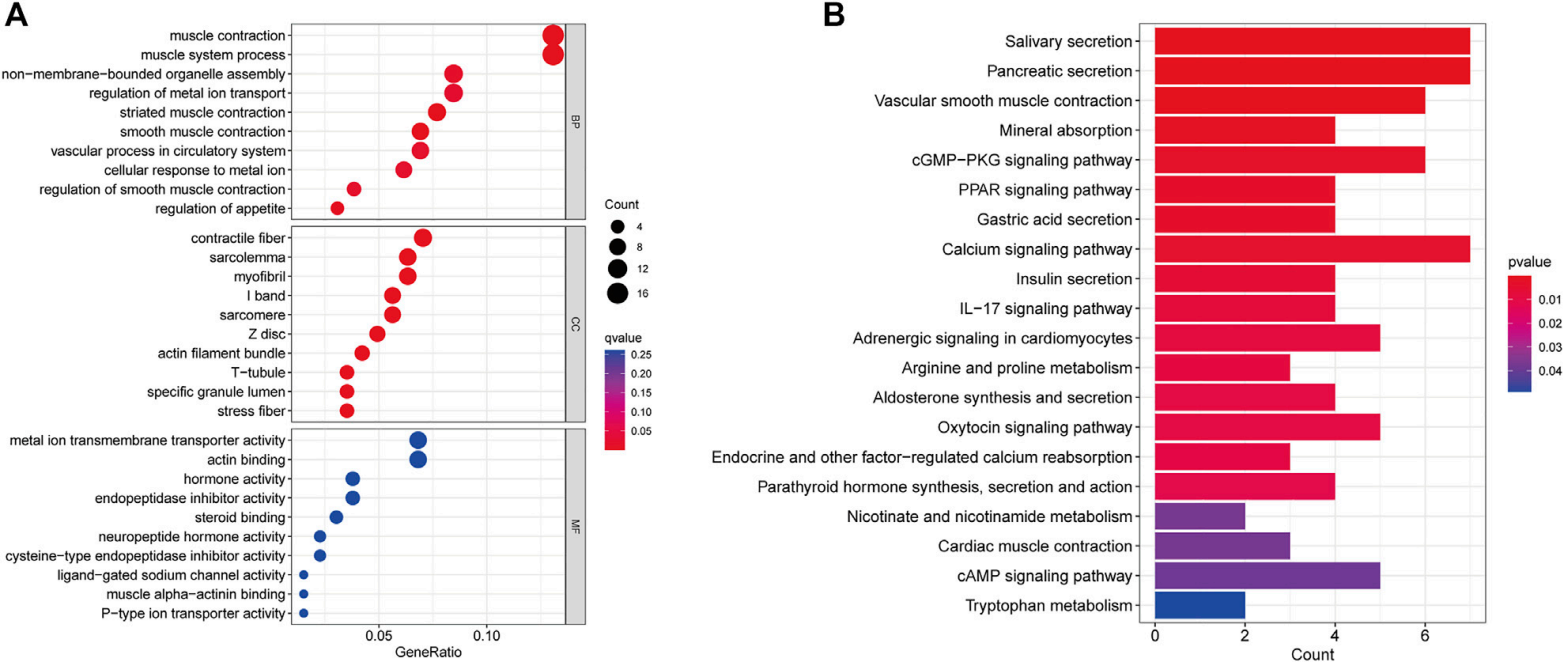
CRL functional enrichment analysis. **(A)** Bubble diagram showing results of GO enrichment analysis of prognostic CRLs. **(B)** Bar plot showing results of KEGG enrichment analysis of prognostic CRLs. BP: biological process, CC: cellular component, MF: molecular function.

Through GO and KEGG analyses, relevant pathways were identified to verify the biological functions of CRLs. First, GO analysis showed that the seven CRLs were mainly involved in the following biological processes: muscle contraction, muscle system process, and vascular process. The main enriched cellular components were contractile fiber and myofibrils. The main enriched molecular functions were metal ion transmembrane transporter activity, actin binding, and endopeptidase inhibitor activity (Figure 5A). In addition, the seven CRLs were associated with the calcium signaling pathway, vascular smooth muscle contraction, and the cGMP-PKG signaling pathway according to the KEGG enrichment analysis (Figure 5B).

### Immune-related functional correlation analysis

**FIGURE 6:**
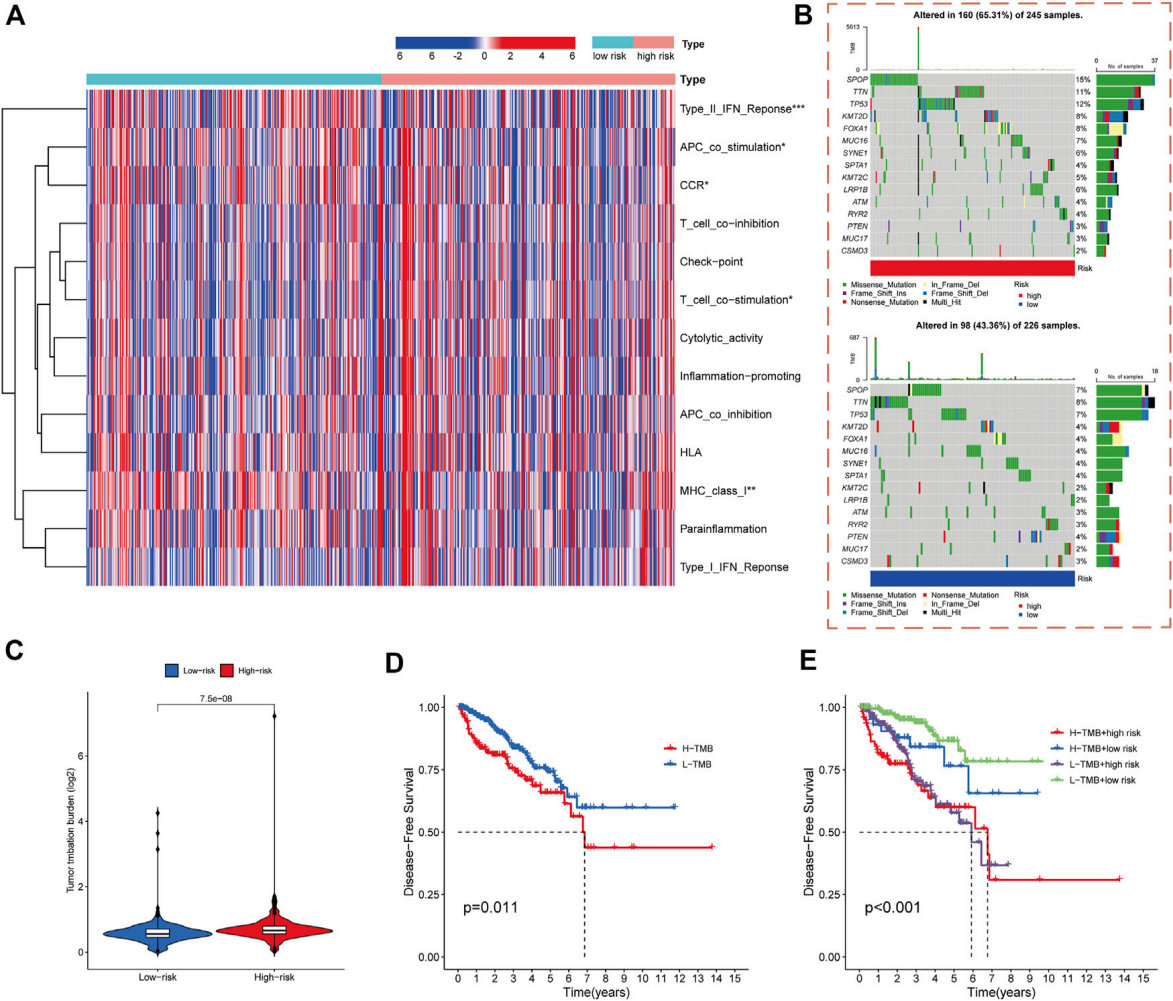
Immune functional and mutation landscape analysis of high- and low-risk cohorts. **(A)** Heatmap of immune function expression in high- and low-risk cohorts. **(B)** Waterfall plot of mutation rates in high- and low-risk cohorts. **(C)** TMB levels in high- and low-risk cohorts. **(D)** Kaplan-Meier analysis of DFS rates in high- and low-TMB cohorts. **(E)** Kaplan-Meier analysis of DFS rates in different TMB and risk score subgroups.

Accumulating evidence indicates a correlation between immunological features and survival in malignant tumors. Therefore, we analyzed the correlations between the cuproptosis-related risk score model and immune-related functions. The results showed that PCa-related lncRNAs were highly associated with the activation of type-II-IFN response, APC co-stimulation, T cell co-stimulation, MHC class I, and chemokine receptors (Figure 6A).

### Analysis of gene mutations in different risk cohorts

The gene mutations in the high- and low-risk cohorts of PCa patients were analyzed. The SPOP, TNN, TP53, KMT2D, FOXA1, MUC16, SYNE1, KMT2C, LRP1B, ATM, RYR2, and MUC17 genes had higher mutation frequencies in the high-risk cohort than in the low-risk cohort, whereas PTEN and CSMD3 had lower mutation frequencies in the high-risk cohort (Figure 6B).

Previous study had shown that high TMB is associated with good survival outcomes in cancer immunotherapy (Samstein et al., 2019). We therefore investigated the correlation between risk score model and TMB. The results indicated that TMB was significantly higher in the high-risk group of PCa patients than those in the low-risk group (*p* < 0.001) (Figure 6C). DFS was significantly worse in the high-TMB group than those in the low-TMB group (*p* = 0.011) (Figure 6D). The predictive ability of risk score model in the low- and high-TMB subgroups was also assessed, demonstrating that the prognostic model presented consistent predictive ability in the high- and low-TMB subgroups. The high- or low-TMB did not influence the predictive efficacy of the model (Figure 6E).

### Drug sensitivity analysis

**FIGURE 7:**
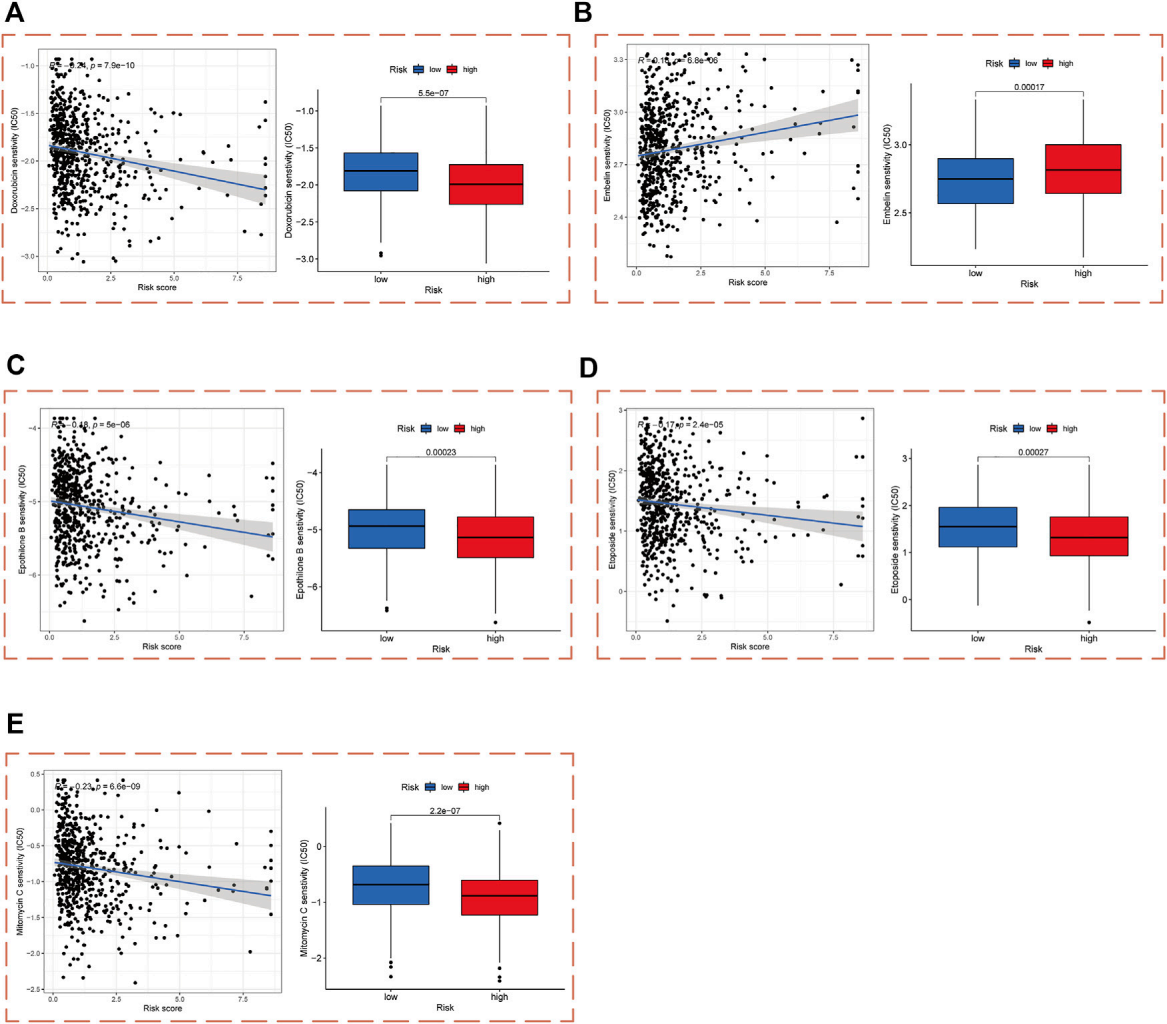
Drug sensitivity analysis in high- and low-risk cohorts. IC50 values of **(A)** Doxorubicin, **(B)** Embelin, **(C)** Epothilone B, **(D)** Etoposide, and **(E)** Mitomycin C in high- and low-risk cohorts.

In order to enable practical application of our PCa risk score model in the treatment of PCa, we performed a drug sensitivity analysis of patients in the high- and low-risk cohorts with chemotherapy and targeted drugs commonly used in PCa. The results demonstrated that patients in the high-risk cohort were significantly more sensitive to doxorubicin, epothilone B, etoposide, and mitomycin C than those in the low-risk cohort, and significantly less sensitive to embelin (Figures 7A–E).

## Discussion

Cuproptosis is a mode of controlled cell death that is different from apoptosis, pyroptosis, necrotic apoptosis, and ferroptosis. It mainly induces cell death by blocking the TCA cycle. Cuproptosis can be considered to be a very promising tumor therapeutic target (Tang et al., 2022; Tsvetkov et al., 2022). Liu et al. showed that PCa cells mainly depend on the TCA cycle in the G1 phase of cell cycle to produce energy; however, in S phase, PCa cells mainly regulate the TCA cycle and glycolysis via the Skp2- IDH1 axis, which provides energy for development of PCa (Liu et al., 2021b). The TCA cycle is not only linked to the initiation of cuproptosis but also supports the growth of PCa (Bader and McGuire, 2020). Hence, we speculated that there might be an association between cuproptosis and PCa. Recent research has shown that cuproptosis-related genes are potential prognostic factors in patients with renal clear cell carcinoma (Bian et al., 2022). However, no link between cuproptosis-related biomarkers and the prognosis of PCa has yet been reported.

In this study, we first explored CRLs and established a risk score model associated with DFS in PCa. First, Cox regression analysis and LASSO regression analysis were conducted to screen CRLs related to the prognosis of PCa. The selected lncRNAs were used to construct a risk score model for predicting the prognosis of PCa patients. According to our risk score model, patients were divided into high-and low-risk cohorts. The DFS of the high-risk cohort in PCa was significantly lower than that of the low-risk cohort (*p* = 0.012). Compared with other clinicopathological variables, such as SM and pathological T and N stage, the risk score showed a better ability to predict the prognosis of PCa patients. The ROC curve also reflected the superior predictive ability of the risk score. To improve the clinical applicability of the model, we also constructed a novel nomogram combining the risk score with clinicopathological indices (age, GS, T stage, N stage, and SM). This nomogram provides an intuitive and quantitative evaluation method for predicting 1-, 3-, and 5-year DFS of PCa patients. In addition, we confirmed the feasibility of our model in a test dataset. Finally, we determined the prognostic value of seven CRLs in PCa (C1orf229, C9orf139, LIPE-AS1, MCPH1-AS1, PRR26, SGMS1-AS1, and SNHG1).

Previous studies have found that lncRNA LIPE-AS1 is overexpressed in a variety of tumor types, including cervical cancer and breast cancer, compared with normal tissues. Several studies have also shown that high expression of LIPE-AS1 is associated with better prognosis (Liu et al., 2021a; Xu et al., 2021; Dai et al., 2022), consistent with our results. Xu et al. showed that the expression of LIPE-AS1 was significantly correlated with that of the PD-L1 gene (Xu et al., 2021). PD-1/PD-L1 immunoblocking therapy is a very promising treatment for advanced PCa (Xu et al., 2021). Therefore, further study of lncRNA LIPE-AS1 may lead to the discovery of new targets for immunotherapy of advanced PCa. Liu et al. reported that lncRNA SGMS1-AS1 could inhibit the proliferation, migration, and invasion of lung adenocarcinoma and could thus represent a therapeutic target (Liu et al., 2021). LncRNA C9orf139 could promote the growth of pancreatic cancer cells by targeting the miR-663a/Sox12 axis, which is a risk factor for prognosis of pancreatic cancer (Ge et al., 2020). Our results also indicated that higher expression of C9orf139 was associated with worse prognosis of PCa patients. No study has yet revealed the mechanisms of C1orf229, MCPH1-AS1, PRR26, and SNHG1 in PCa or other malignant tumors. Therefore, the associations between these lncRNAs and PCa require further exploration. We performed GO and KEGG enrichment analyses to explore possible mechanisms in the high and low-risk cohorts; however, the specific mechanism of CRLs in PCa is still not clear. This will be our main research direction in the future.

At present, the main challenge of PCa therapy is not the initial treatment but the choice of therapeutic method for CRPC. CRPC is often caused by drug resistance after long-term endocrine therapy. Emerging immunotherapies may represent a new strategy to optimize the treatment of PCa patients with CRPC (Lu et al., 2017; Cha et al., 2020). Our results indicated significant differences in the activated levels of Type-II-IFN response, APC co-stimulation, T cell co-stimulation, chemokine receptor, and MHC class I between the high- and low-risk cohorts. Thus, these immune functions might have important roles in the occurrence and development of PCa. Previous studies have shown that PCa has low sensitivity to immune checkpoint blockade treatment owing to a lack of T cell infiltration and Type-I/II IFN characteristics (Annels et al., 2021). Studies have also shown that OX40-specific agonists can improve immunotherapeutic response rates in PCa patients by using T cell co-stimulation to induce T cell activation and anti-tumor immunity (Sturgill et al., 2021). Oncolytic viruses could also guide the local expression of Type-II-IFN to induce PD-L1 and PD-L2 to act on tumor cells, thereby making PCa sensitive to immune checkpoint blockade (Samson et al., 2018). In addition to activation of immune functions, the high-risk cohort showed higher TMB. Previous studies have shown that higher TMB is associated with higher survival rates in patients with various tumor types who received immune checkpoint inhibitors (Samstein et al., 2019). Our results demonstrate that high-risk patients are likely to be sensitive to doxorubicin, epothilone B, etoposide and mitomycin C but resistant to embelin. Doxorubicin is among the most widely used anti-tumor drugs for PCa treatment, but it has clinical non-selectivity and noticeable adverse effects. In recent years, abundant research has shown that nano-drugs can be used as doxorubicin carriers (SreeHarsha et al., 2019; Sun et al., 2021). Such targeted drug delivery could improve the efficacy of drugs. Combined with our results, this indicates that patients in the high-risk cohort might have more drug options and provides a basis for individualized drug therapy for PCa patients.

To the best of our knowledge, this was the first study to construct a prognostic model based on CRLs in PCa. Our PCa risk score model was based on complete RNA-seq data of the TCGA PCa cohort and 136 cases of PCa from the Changhai Hospital; thus, it could provide reliable and robust prognosis prediction for Chinese PCa patients. Our research had some limitations. First, the details of the mechanisms involving cuproptosis and CRLs in PCa are still not clear and need to be further elucidated by a large number of experiments. Second, we only used TCGA data and the Changhai Hospital data for internal validation. External validation is needed to confirm the applicability of our model.

In conclusion, we constructed a PCa risk score model consisting of seven CRLs. The model was independent of clinicopathological features and could accurately predict the prognosis of PCa patients. In addition, GO and KEGG analyses revealed potential pathways involving cuproptosis in PCa. Immune function analysis, TMB analysis, and drug sensitivity analysis provided a basis for further exploration of chemotherapy and targeted therapies for PCa. Our model may serve as a potential prognostic indicator, and our findings provide potential new directions for studying CRLs in PCa.

## Data Availability

The original contributions presented in the study are included in the article/Supplementary Material, further inquiries can be directed to the corresponding authors.
