# Supplementary figures and images for: Construction and validation of a novel cuproptosis-related long noncoding RNA signature for predicting the outcome of prostate cancer
Source: Front Genet. 2022 Dec 6;13:976850. doi: 10.3389/fgene.2022.976850 (PMC9763621; doi:10.3389/fgene.2022.976850)

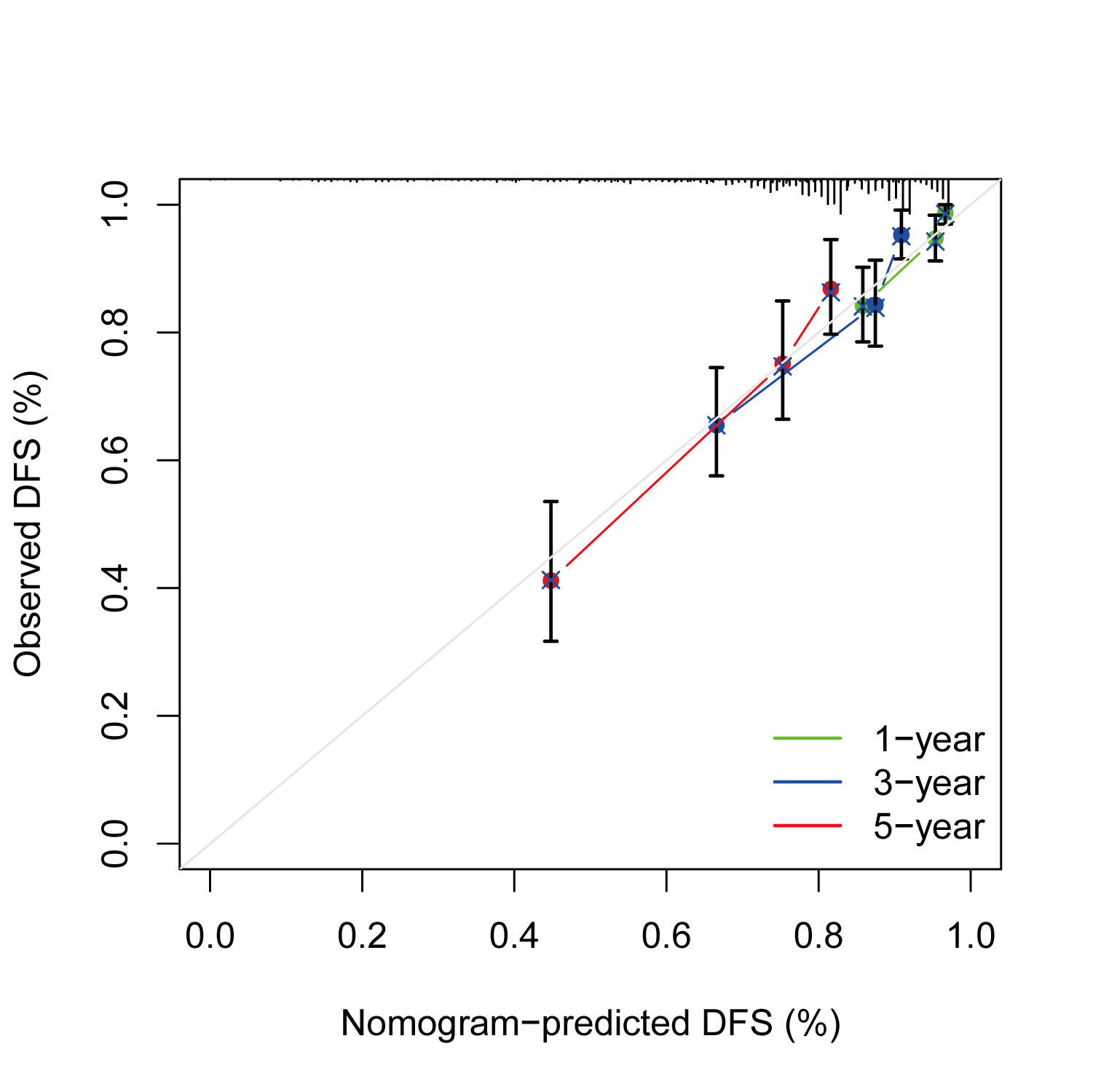

Supplement: Supplementary file 1 [file Image3.TIF]

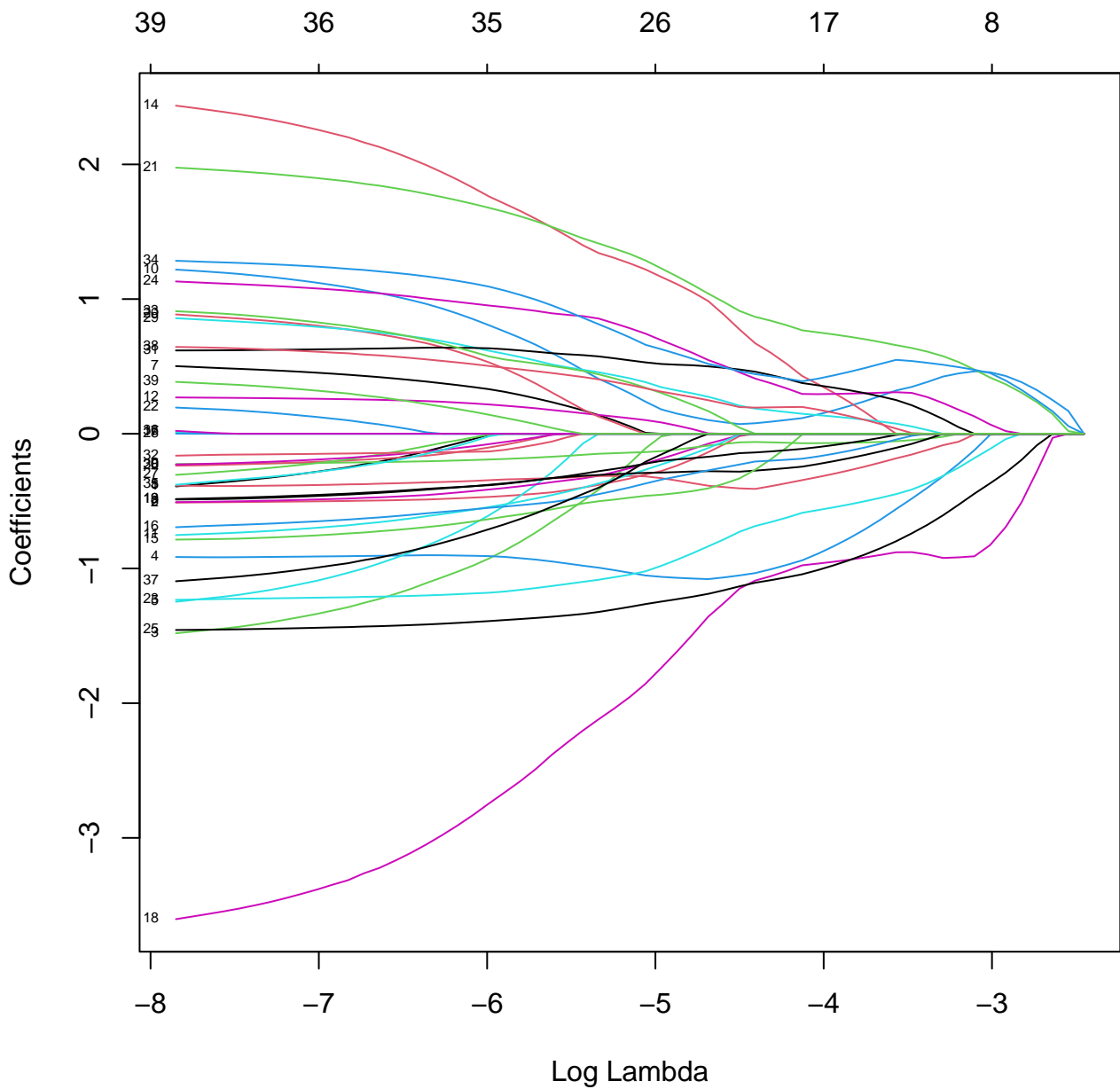

Supplement: Supplementary file 2 [file DataSheet1.ZIP › Source data for review purpose only/Source data/06.model/lasso.lambda.pdf]

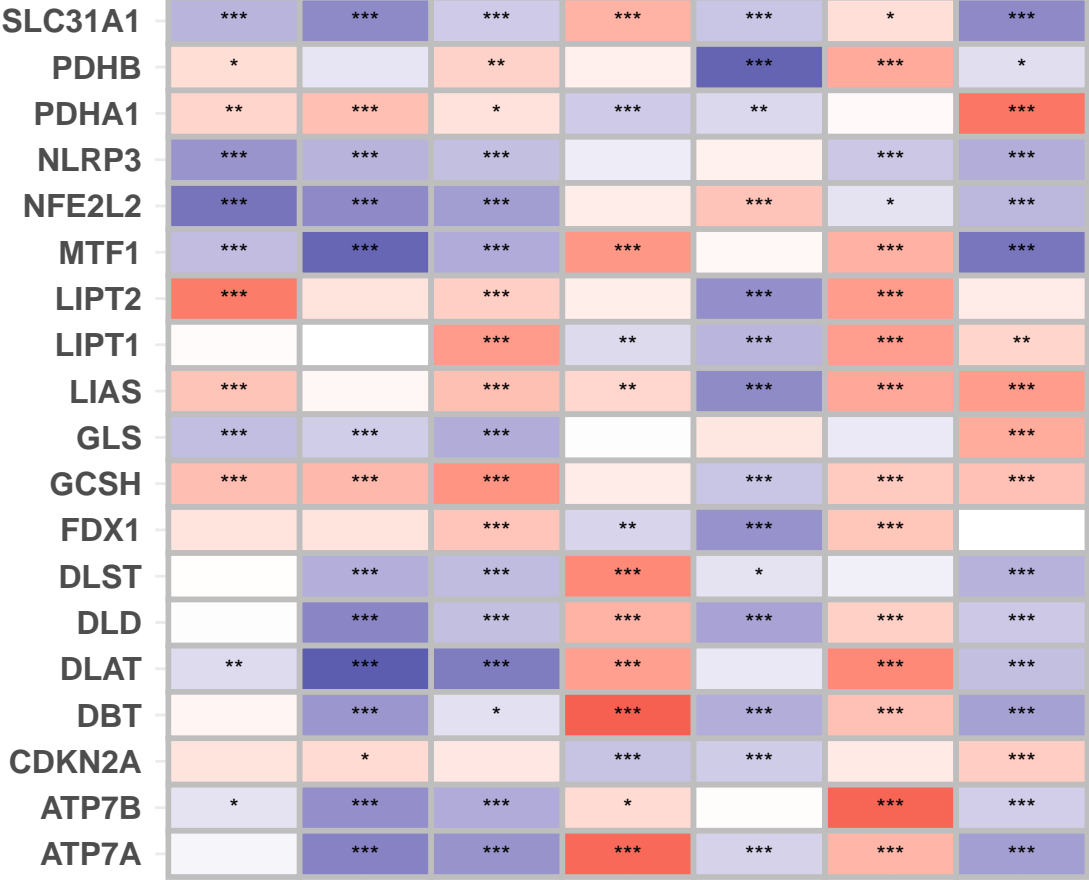

\*\*\* p<0.001  
\*\* p<0.01  
\* p<0.05

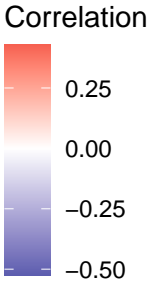

Supplement: Supplementary file 2 [file DataSheet1.ZIP › Source data for review purpose only/Source data/08.corplot/cor.pdf]

Risk    + High risk    + Low risk

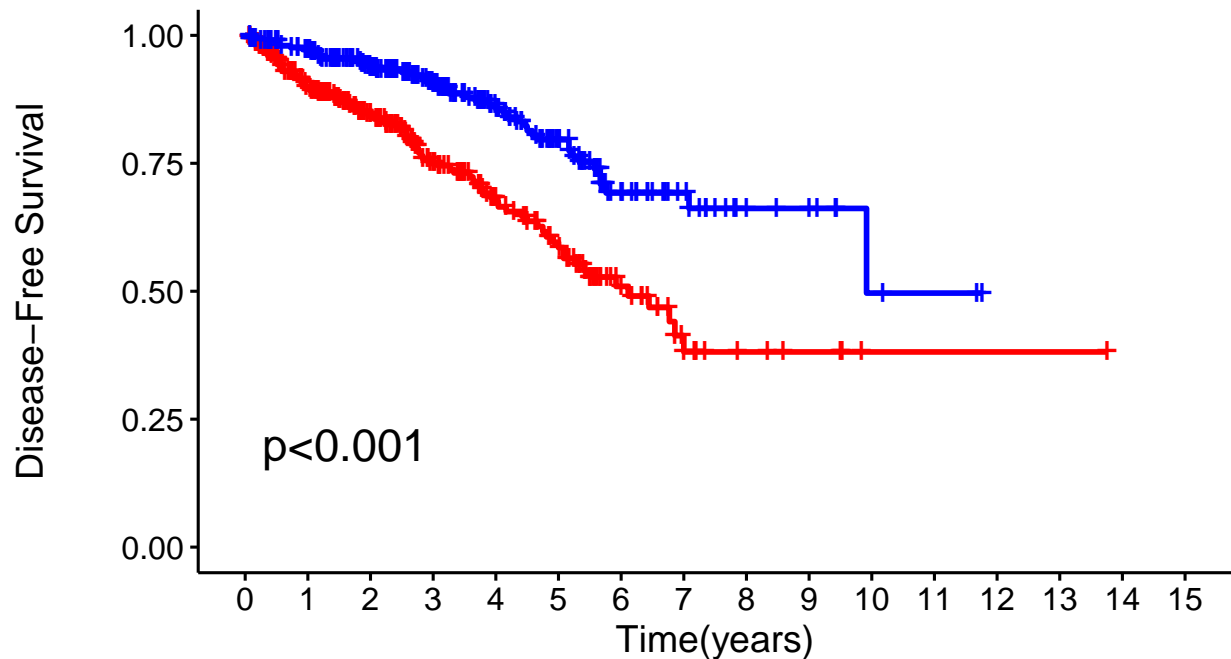

| Risk      | Time(years) |     |     |     |     |    |    |    |    |   |    |    |    |    |    |    |
|-----------|-------------|-----|-----|-----|-----|----|----|----|----|---|----|----|----|----|----|----|
|           | 0           | 1   | 2   | 3   | 4   | 5  | 6  | 7  | 8  | 9 | 10 | 11 | 12 | 13 | 14 | 15 |
| High risk | 306         | 232 | 167 | 114 | 77  | 57 | 28 | 13 | 6  | 4 | 1  | 1  | 1  | 1  | 0  | 0  |
| Low risk  | 307         | 270 | 216 | 156 | 110 | 79 | 37 | 24 | 10 | 8 | 3  | 2  | 0  | 0  | 0  | 0  |

Supplement: Supplementary file 2 [file DataSheet1.ZIP › Source data for review purpose only/Source data/09.survival/surv.all.pdf]

Disease-Free Survival

Risk + High risk + Low risk

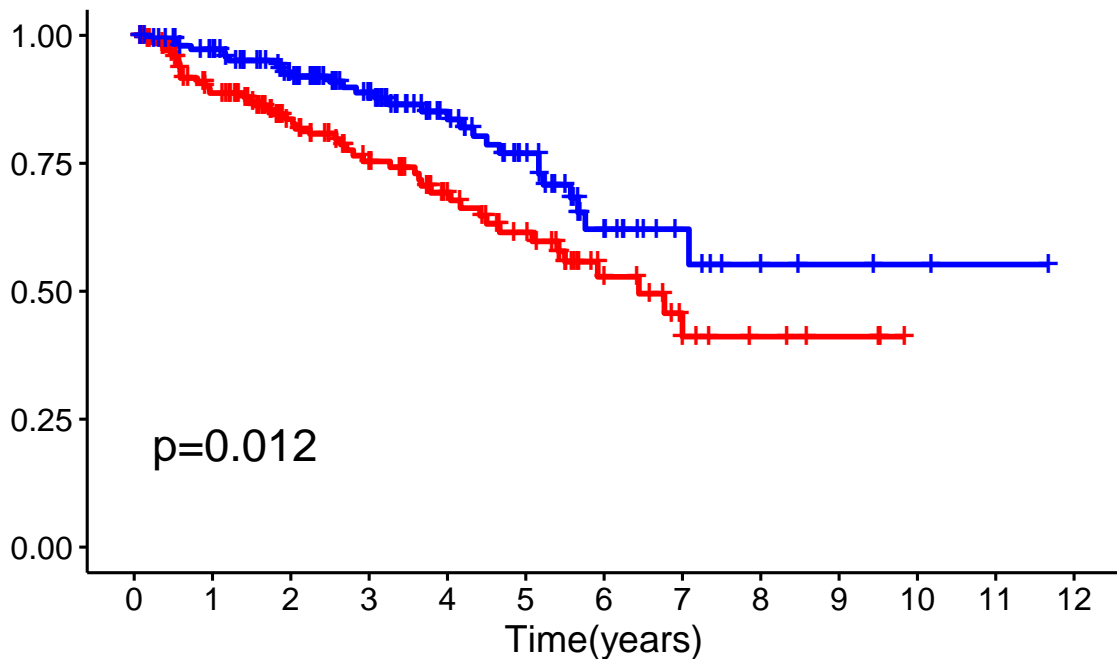

$p=0.012$

Risk

High risk

153 120 88 68 49 36 18 10 5 3 0 0 0

Low risk

153 137 111 83 56 40 19 9 5 3 2 1 0

Time(years)

Supplement: Supplementary file 2 [file DataSheet1.ZIP › Source data for review purpose only/Source data/09.survival/surv.test.pdf]

Risk    + High risk    + Low risk

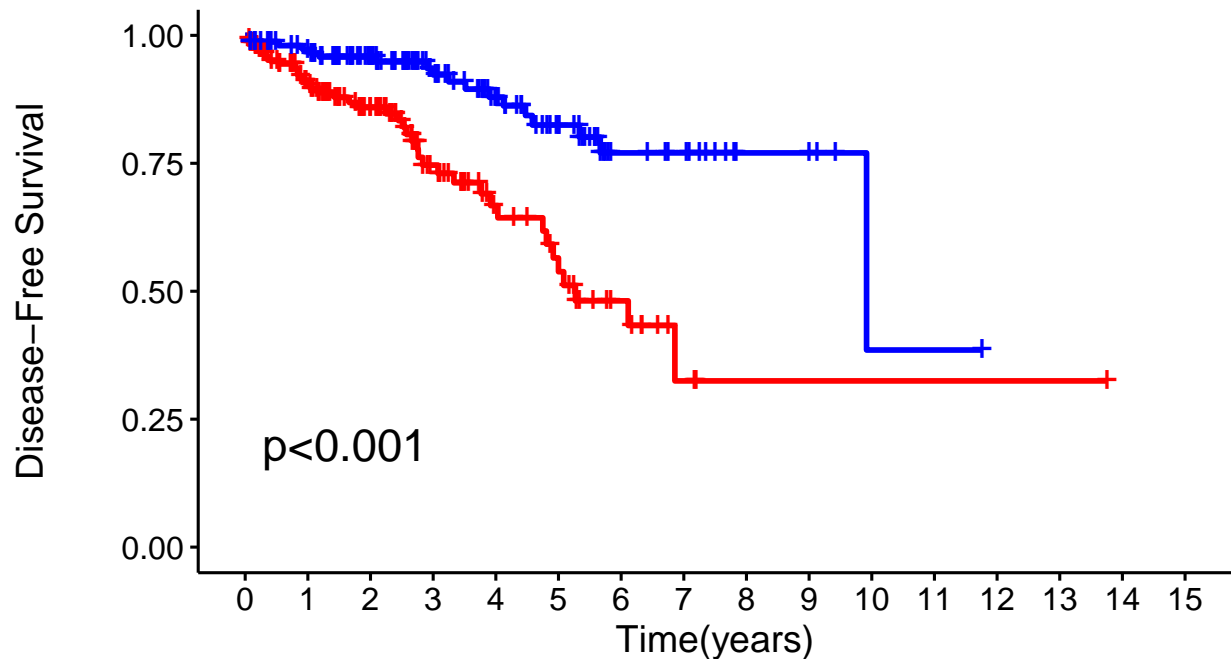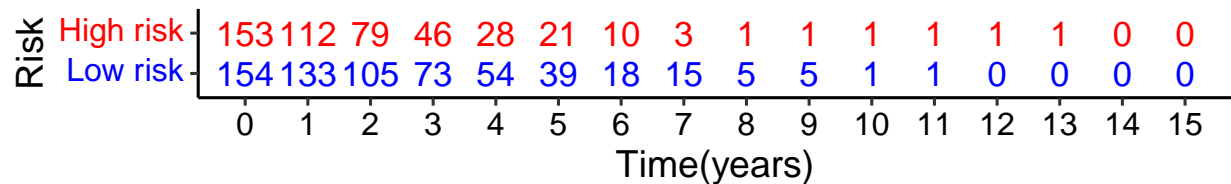

Supplement: Supplementary file 2 [file DataSheet1.ZIP › Source data for review purpose only/Source data/09.survival/surv.train.pdf]

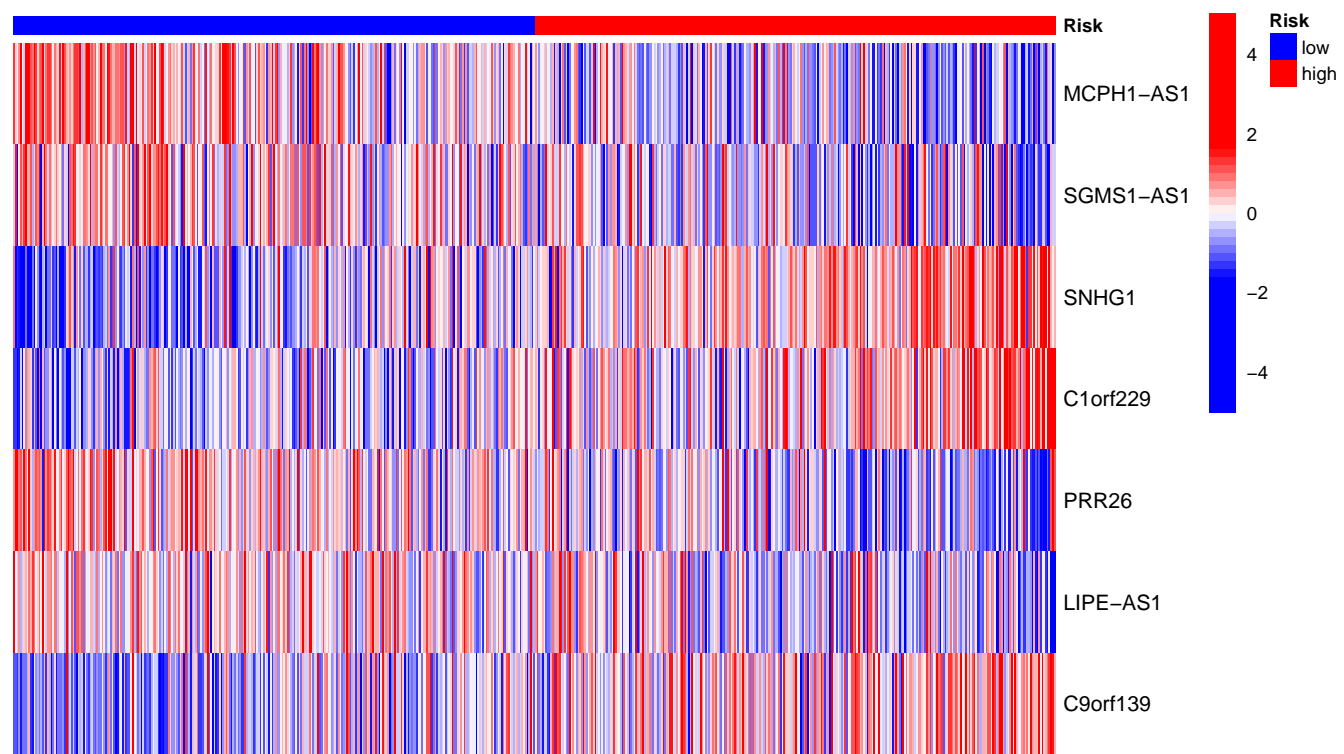

Supplement: Supplementary file 2 [file DataSheet1.ZIP › Source data for review purpose only/Source data/10.riskPlot/all.heatmap.pdf]

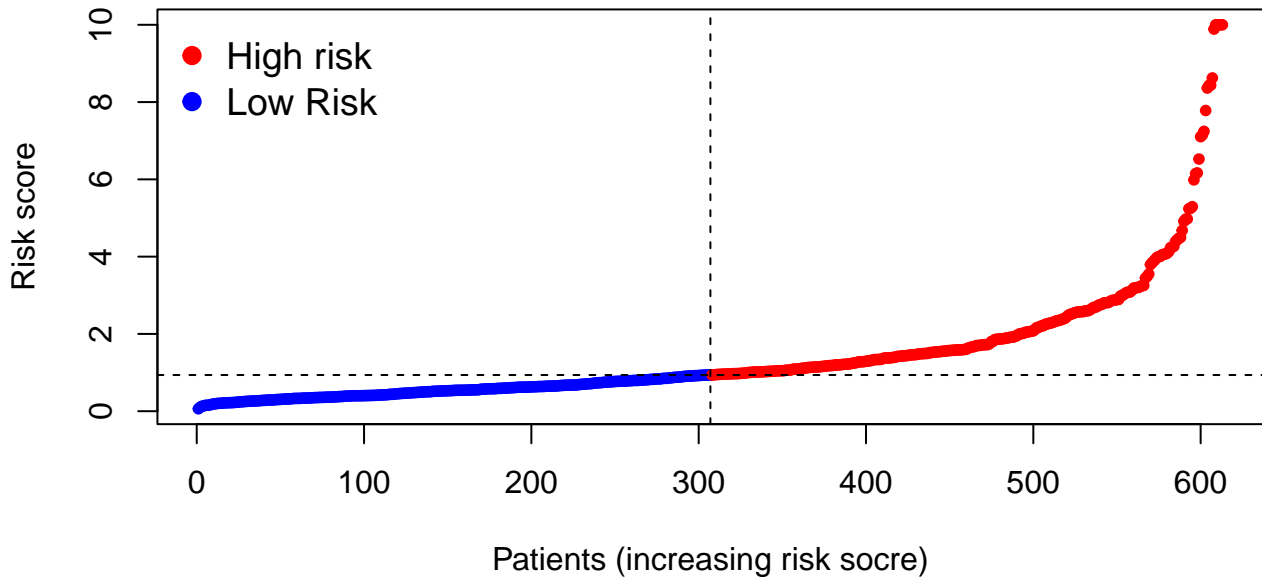

Supplement: Supplementary file 2 [file DataSheet1.ZIP › Source data for review purpose only/Source data/10.riskPlot/all.riskScore.pdf]

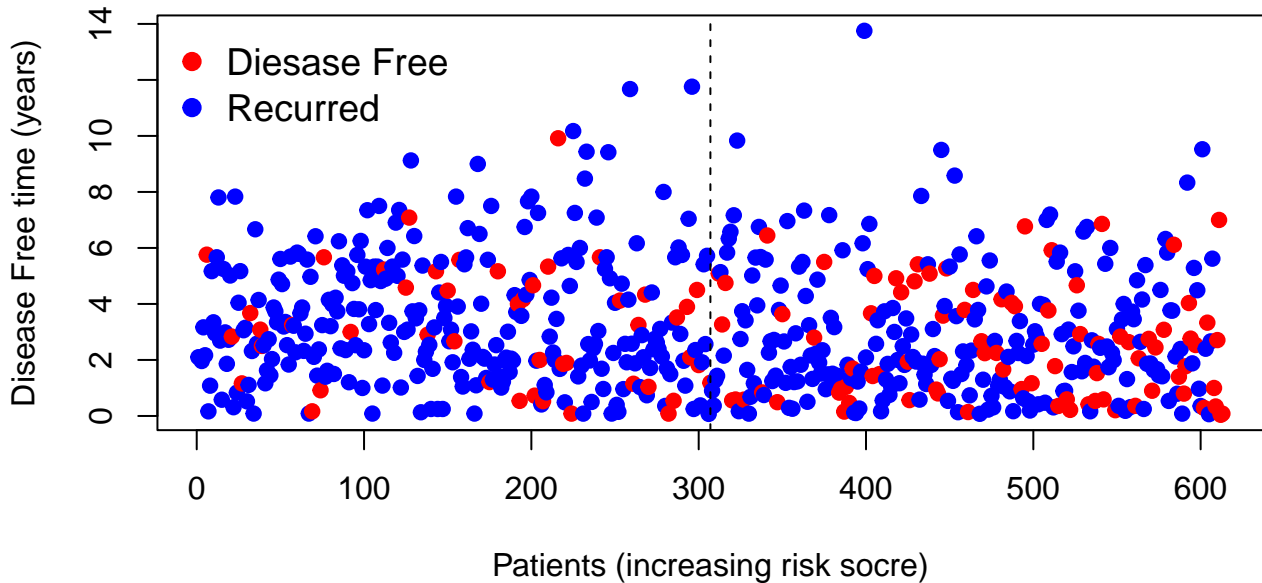

Supplement: Supplementary file 2 [file DataSheet1.ZIP › Source data for review purpose only/Source data/10.riskPlot/all.survStat.pdf]

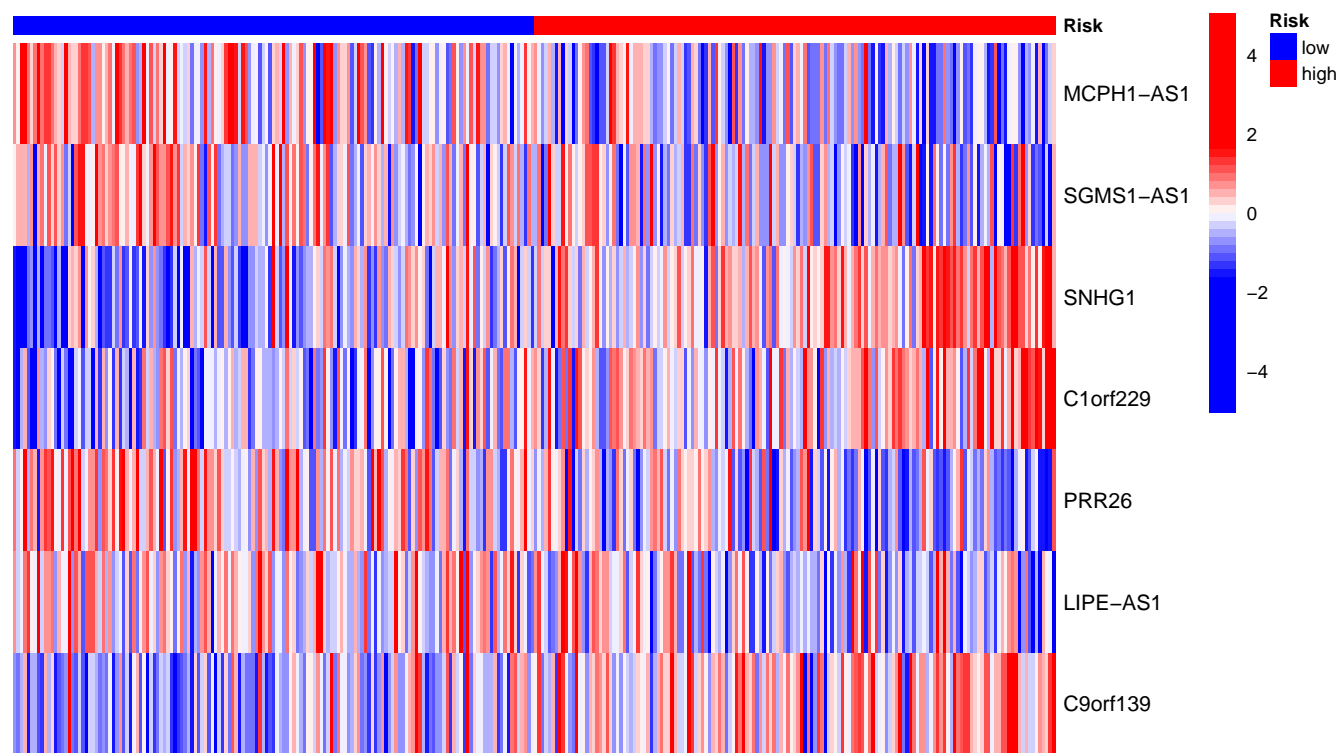

Supplement: Supplementary file 2 [file DataSheet1.ZIP › Source data for review purpose only/Source data/10.riskPlot/test.heatmap.pdf]

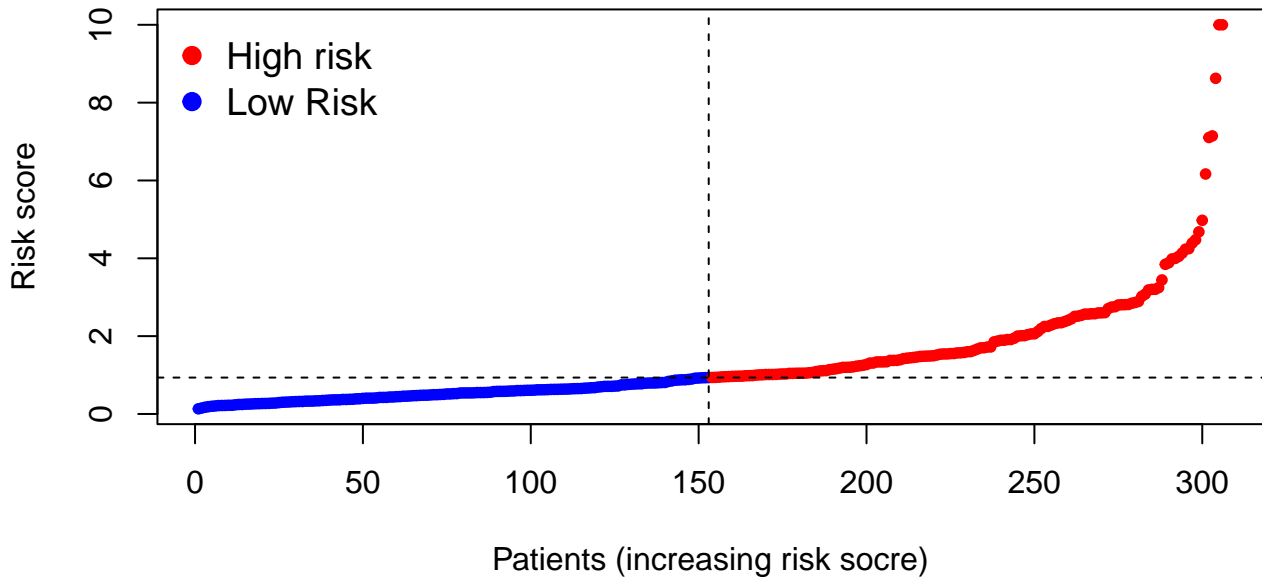

Supplement: Supplementary file 2 [file DataSheet1.ZIP › Source data for review purpose only/Source data/10.riskPlot/test.riskScore.pdf]

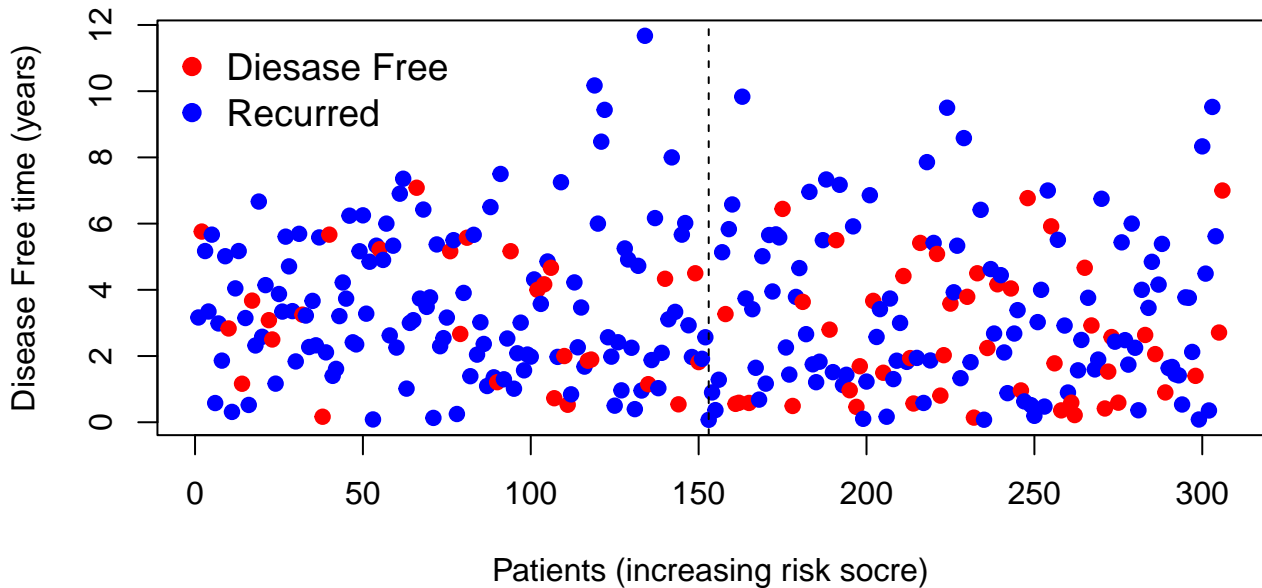

Supplement: Supplementary file 2 [file DataSheet1.ZIP › Source data for review purpose only/Source data/10.riskPlot/test.survStat.pdf]

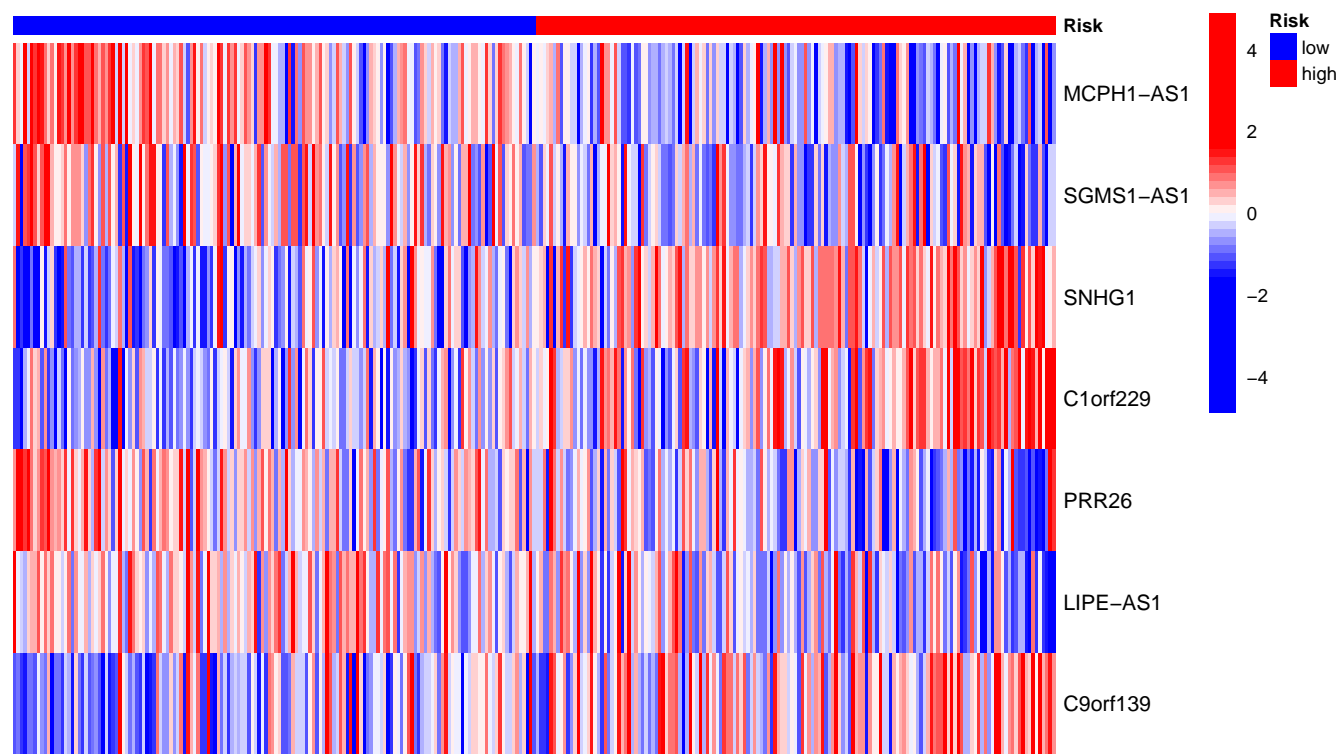

Supplement: Supplementary file 2 [file DataSheet1.ZIP › Source data for review purpose only/Source data/10.riskPlot/train.heatmap.pdf]

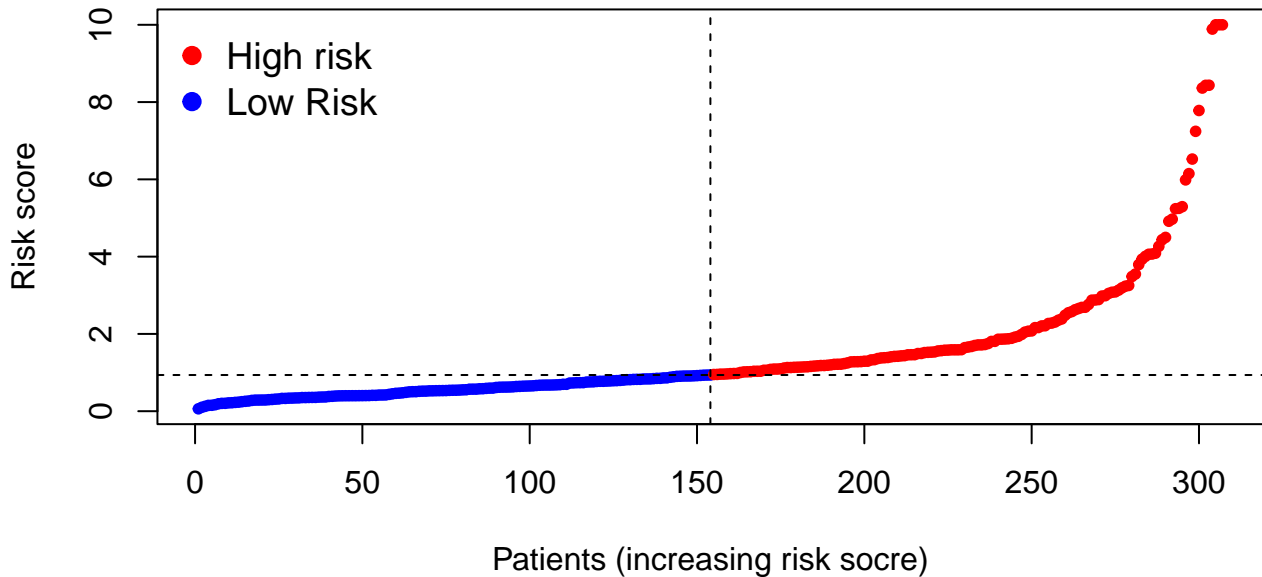

Supplement: Supplementary file 2 [file DataSheet1.ZIP › Source data for review purpose only/Source data/10.riskPlot/train.riskScore.pdf]

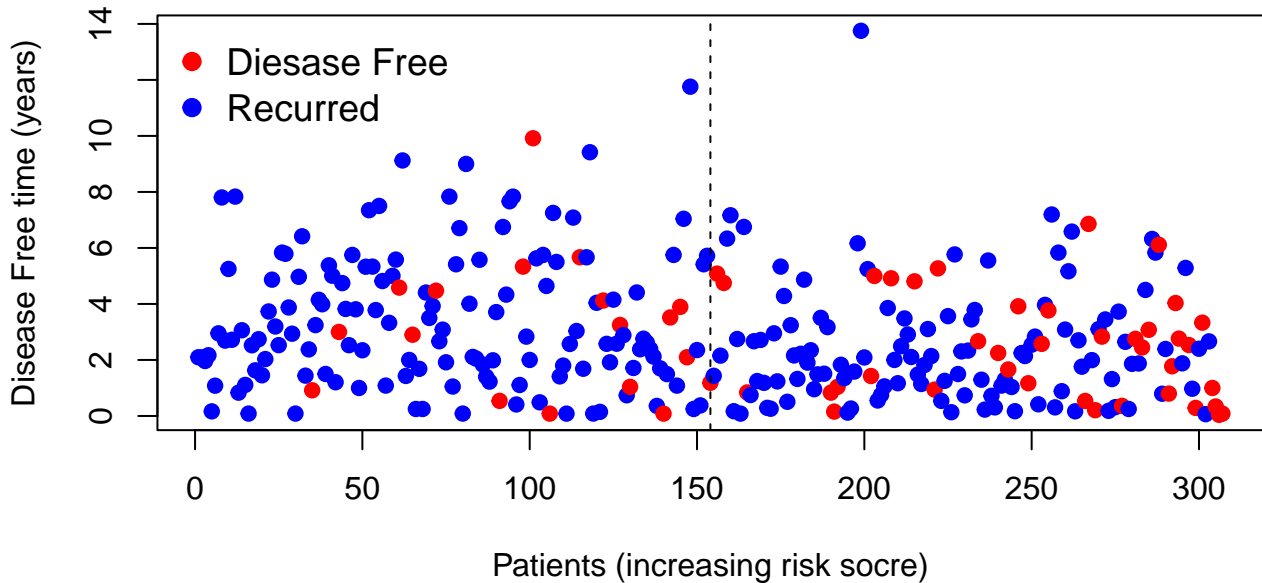

Supplement: Supplementary file 2 [file DataSheet1.ZIP › Source data for review purpose only/Source data/10.riskPlot/train.survStat.pdf]

|                   | pvalue | Hazard ratio       |
|-------------------|--------|--------------------|
| Age               | 0.199  | 1.017(0.991–1.043) |
| `Gleason score`   | <0.001 | 1.679(1.349–2.088) |
| `Surgical Margin` | 0.320  | 1.224(0.822–1.825) |
| T                 | 0.026  | 1.526(1.052–2.213) |
| N                 | 0.298  | 0.775(0.480–1.251) |
| riskScore         | 0.008  | 1.057(1.015–1.101) |

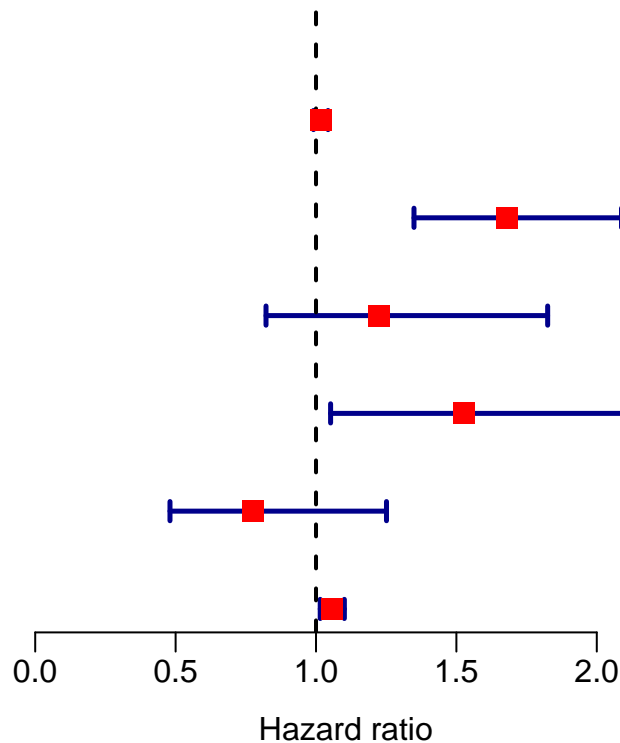

Supplement: Supplementary file 2 [file DataSheet1.ZIP › Source data for review purpose only/Source data/11.indep/all.multiCox.pdf]

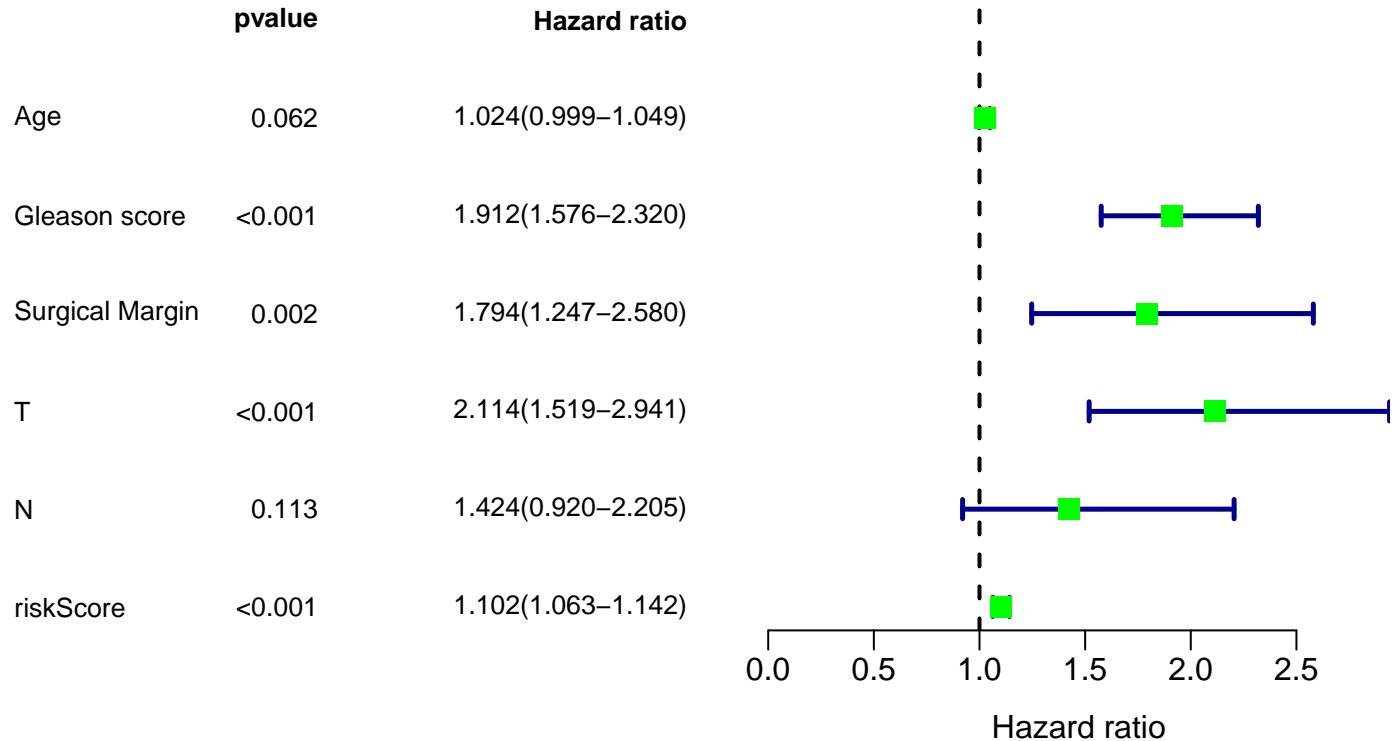

Supplement: Supplementary file 2 [file DataSheet1.ZIP › Source data for review purpose only/Source data/11.indep/all.uniCox.pdf]

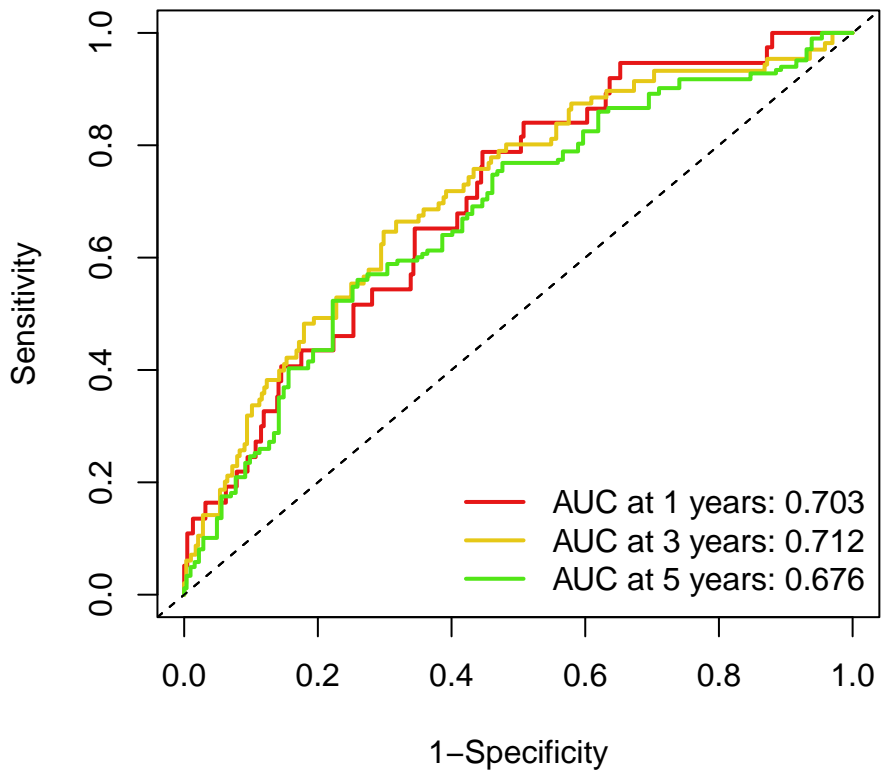

Supplement: Supplementary file 2 [file DataSheet1.ZIP › Source data for review purpose only/Source data/12.ROC/ROC.pdf]

Sensitivity

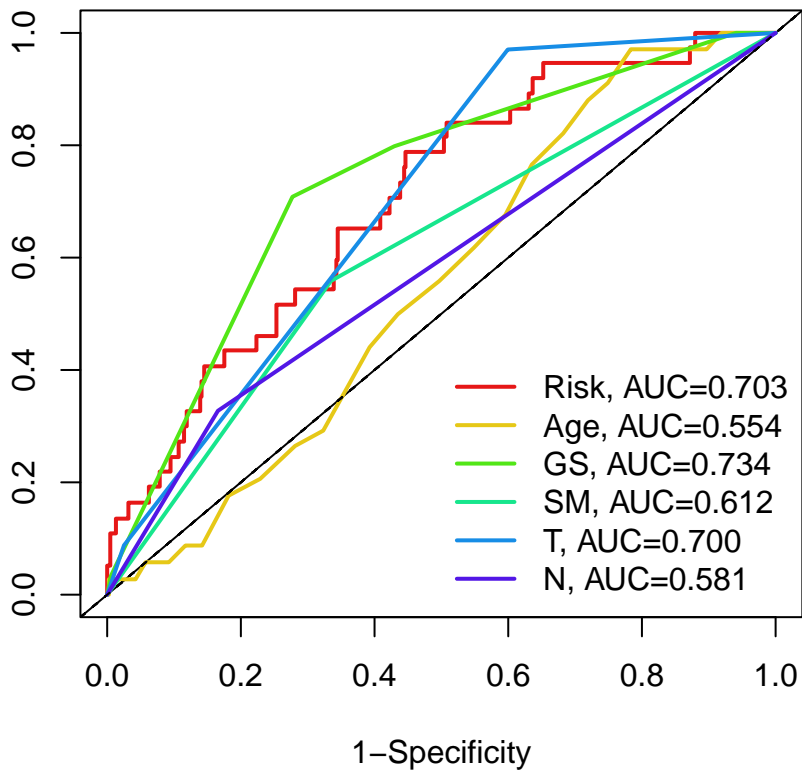

Supplement: Supplementary file 2 [file DataSheet1.ZIP › Source data for review purpose only/Source data/12.ROC/cliROC.pdf]

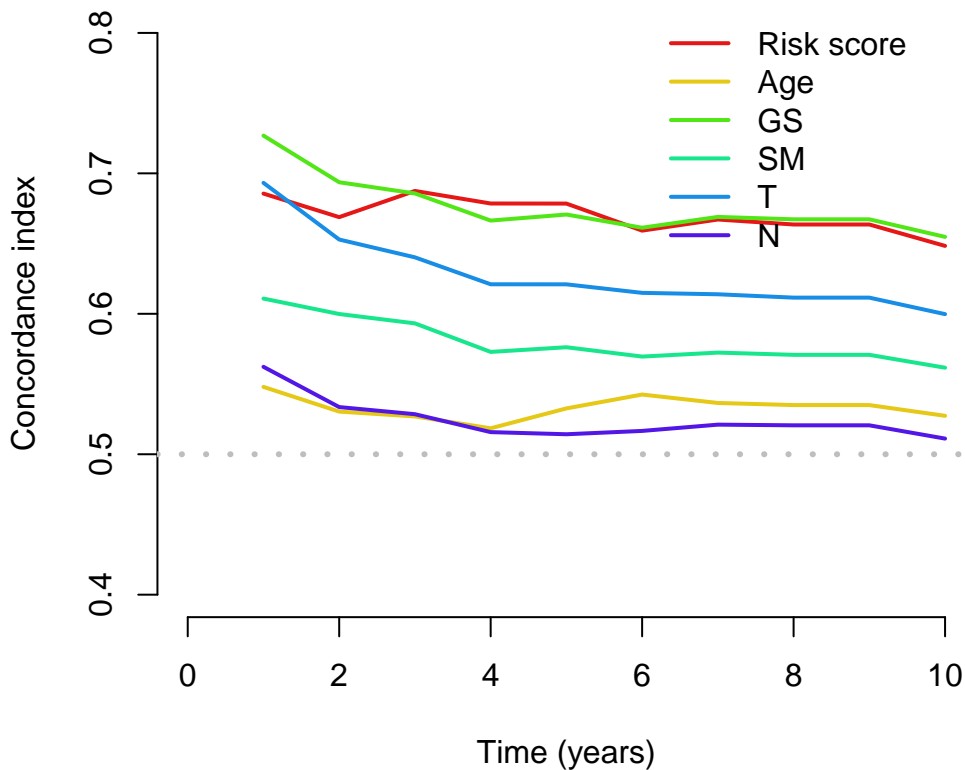

Supplement: Supplementary file 2 [file DataSheet1.ZIP › Source data for review purpose only/Source data/13.C-index/C-index.pdf]

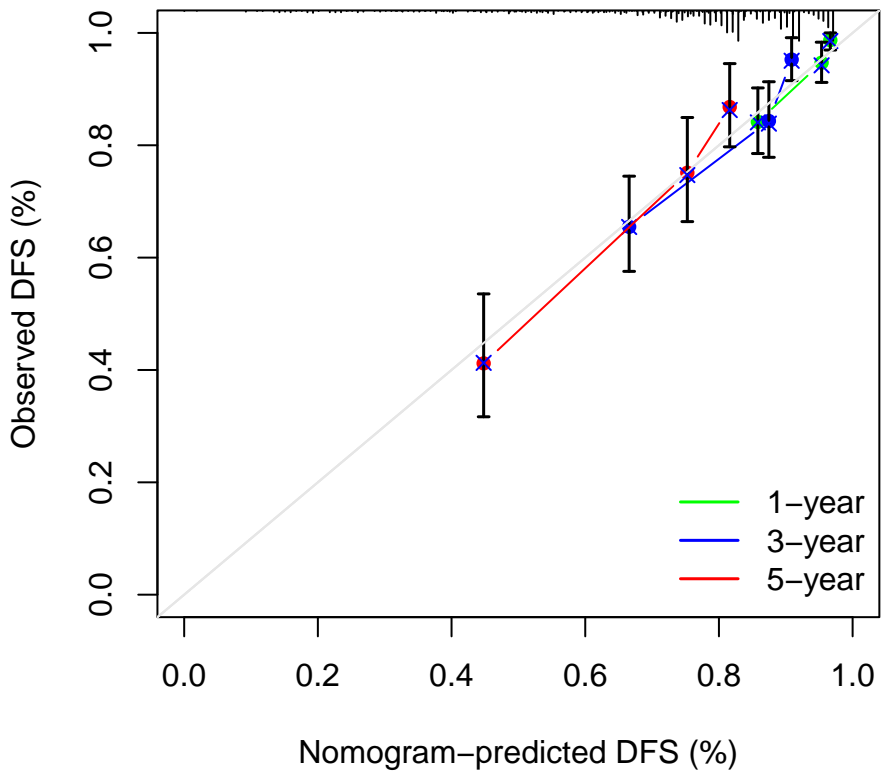

Supplement: Supplementary file 2 [file DataSheet1.ZIP › Source data for review purpose only/Source data/14.Nomo/calibration.pdf]

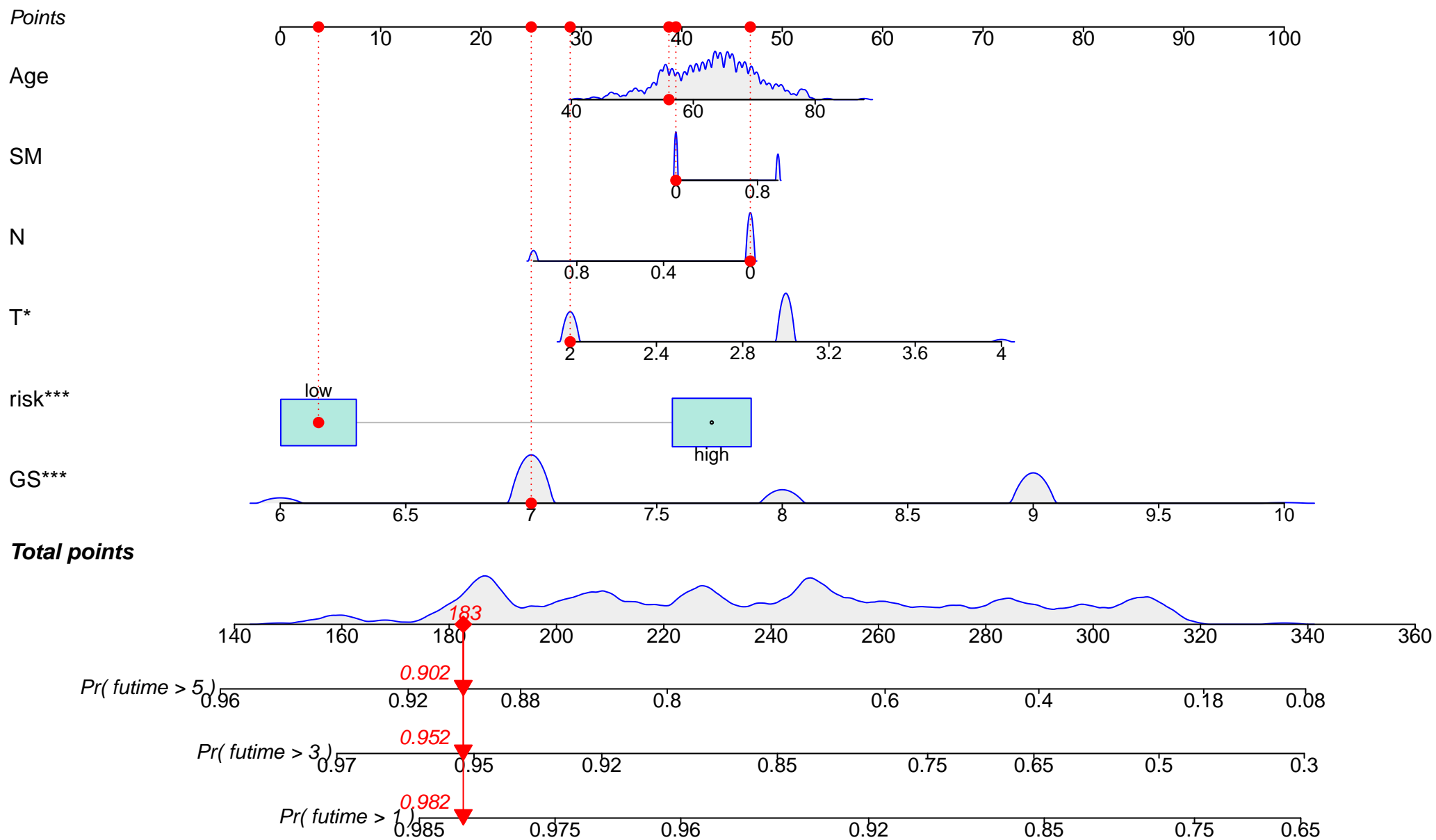

Supplement: Supplementary file 2 [file DataSheet1.ZIP › Source data for review purpose only/Source data/14.Nomo/nomogram.pdf]

# Patients with <63

Risk + high + low

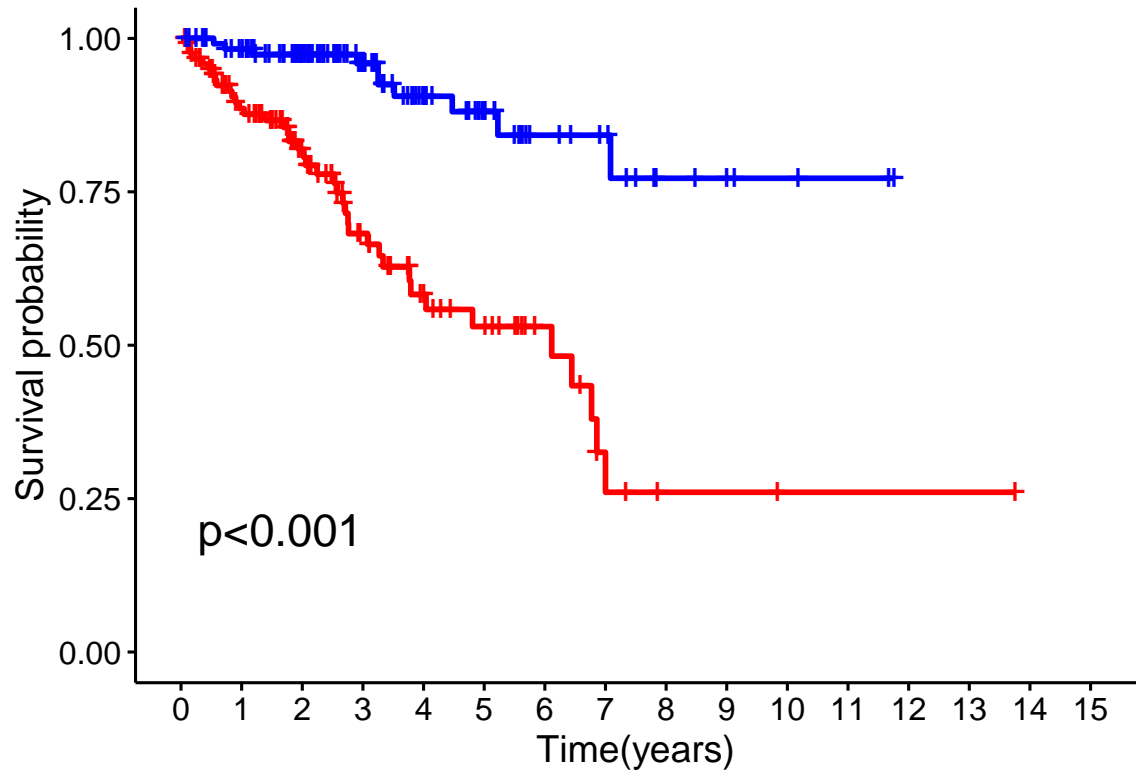

Supplement: Supplementary file 2 [file DataSheet1.ZIP › Source data for review purpose only/Source data/15.cliGroupSur/survival.Ageí▄63.pdf]

# Patients with >63

Risk + high + low

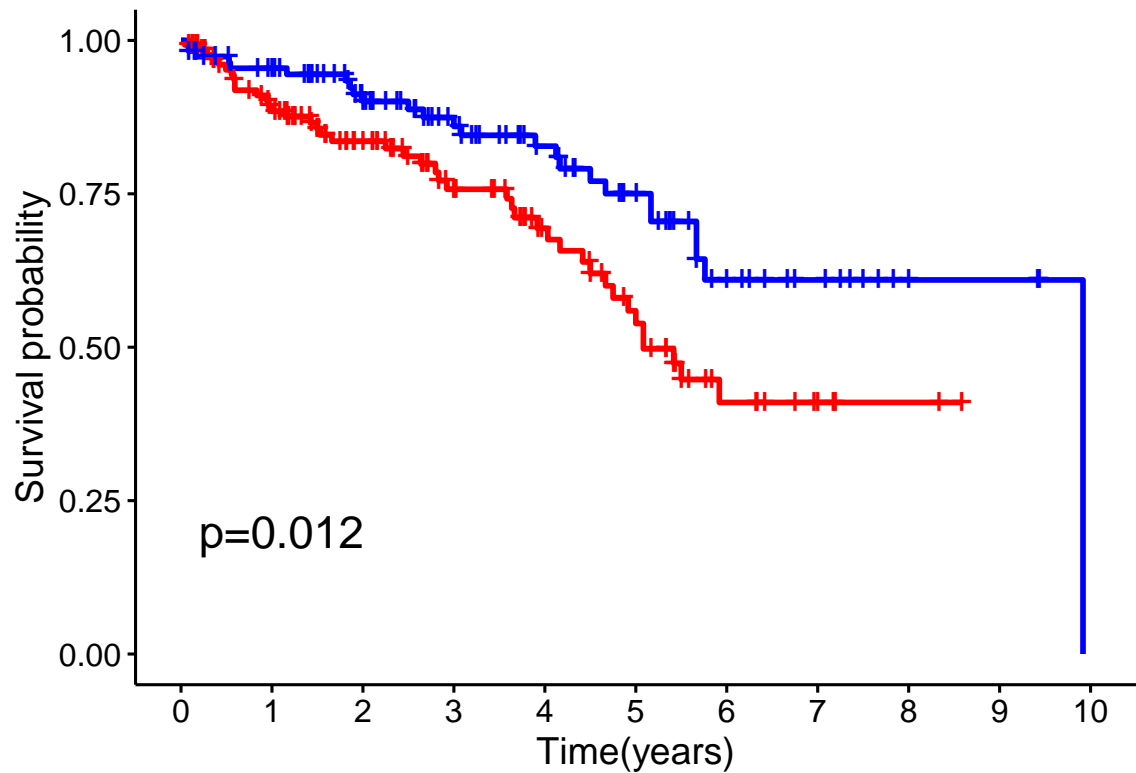

Supplement: Supplementary file 2 [file DataSheet1.ZIP › Source data for review purpose only/Source data/15.cliGroupSur/survival.Ageú╛63.pdf]

# Patients with 6-7

Risk + high + low

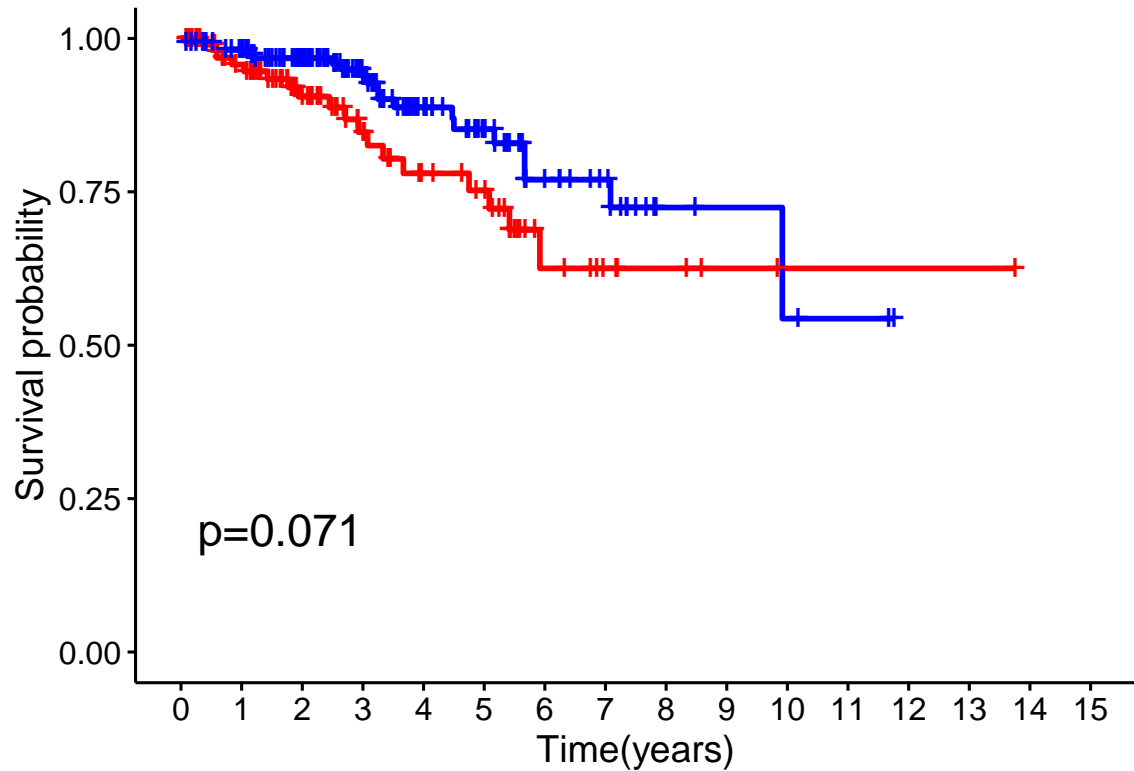

Supplement: Supplementary file 2 [file DataSheet1.ZIP › Source data for review purpose only/Source data/15.cliGroupSur/survival.GS_6-7.pdf]

# Patients with 8–10

Risk + high + low

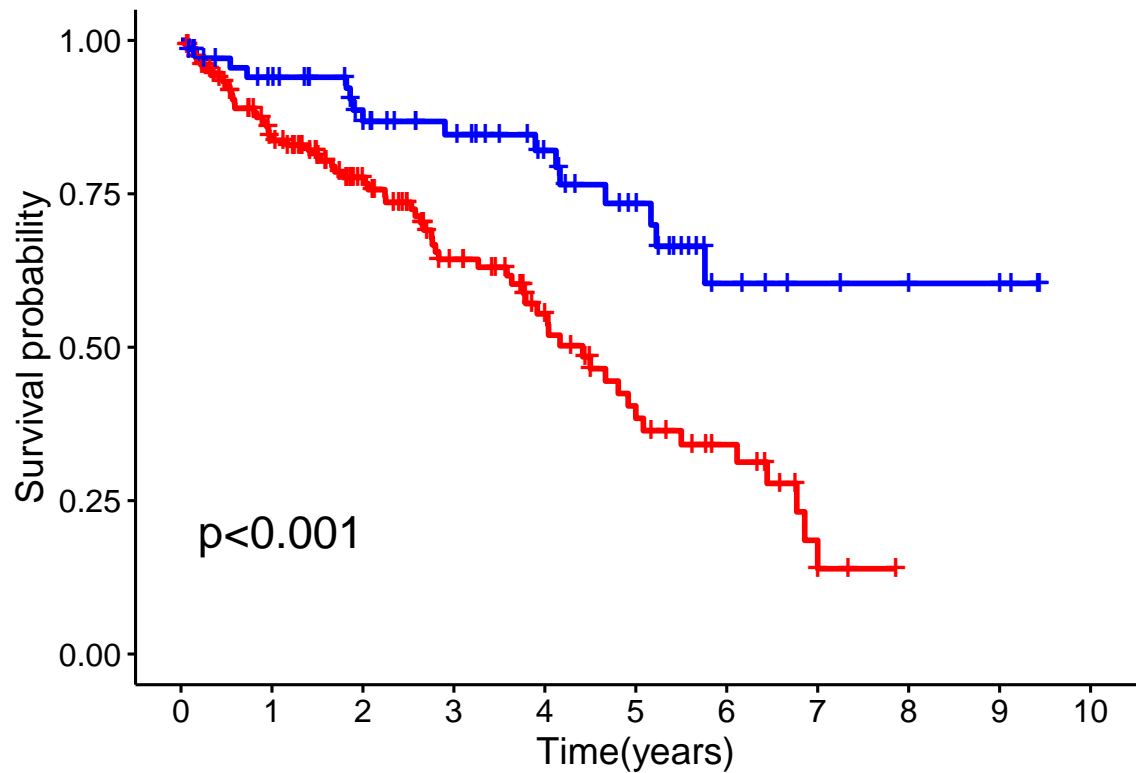

Supplement: Supplementary file 2 [file DataSheet1.ZIP › Source data for review purpose only/Source data/15.cliGroupSur/survival.GS_8-10.pdf]

# Patients with N0

Risk + high + low

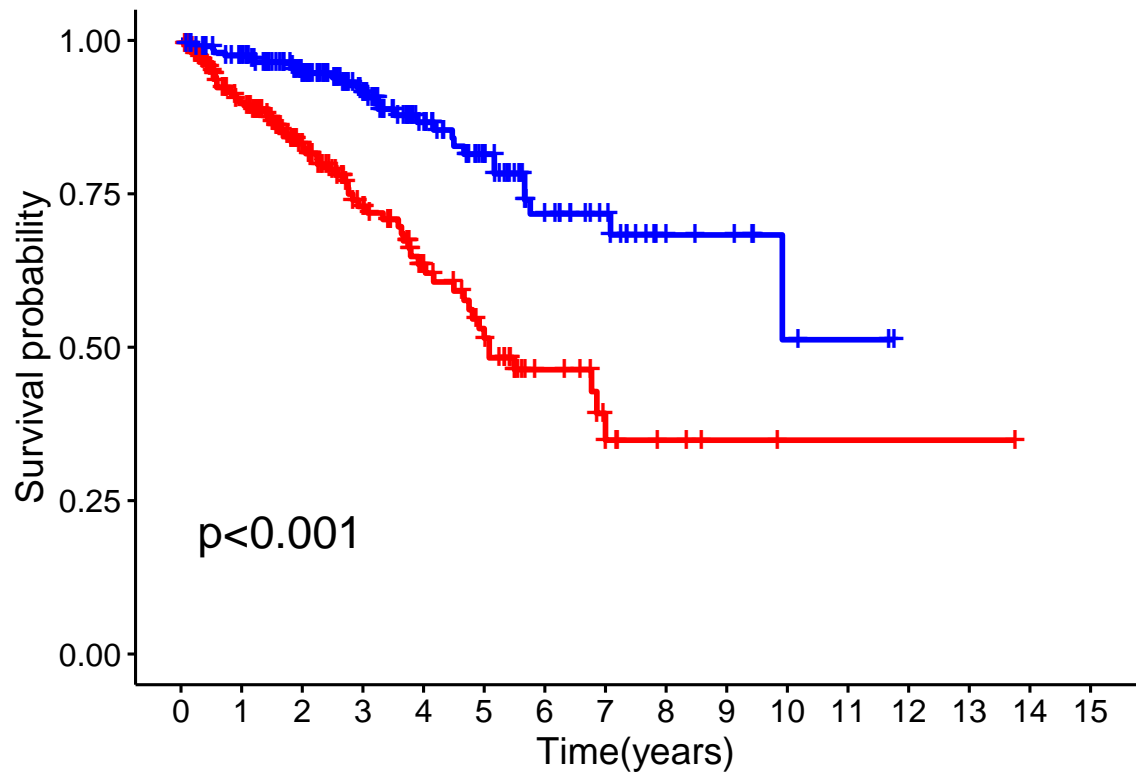

Supplement: Supplementary file 2 [file DataSheet1.ZIP › Source data for review purpose only/Source data/15.cliGroupSur/survival.N_N0.pdf]

# Patients with N1

Risk + high + low

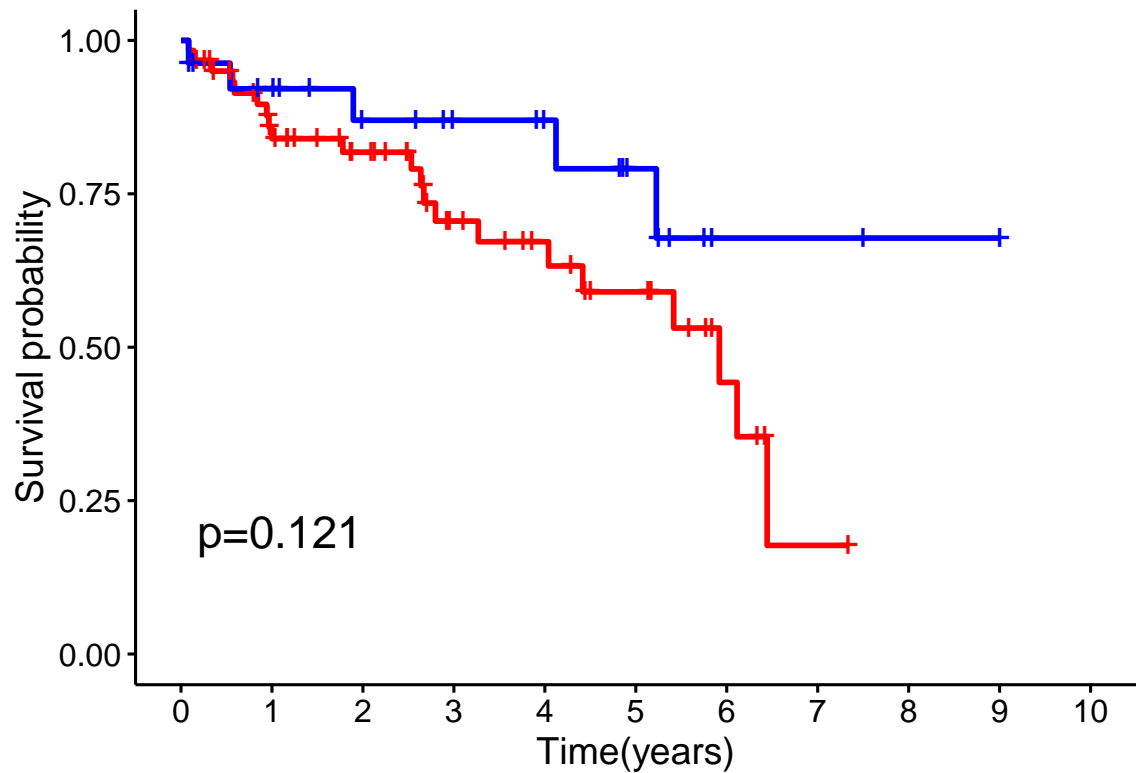

Supplement: Supplementary file 2 [file DataSheet1.ZIP › Source data for review purpose only/Source data/15.cliGroupSur/survival.N_N1.pdf]

# Patients with NO

Risk + high + low

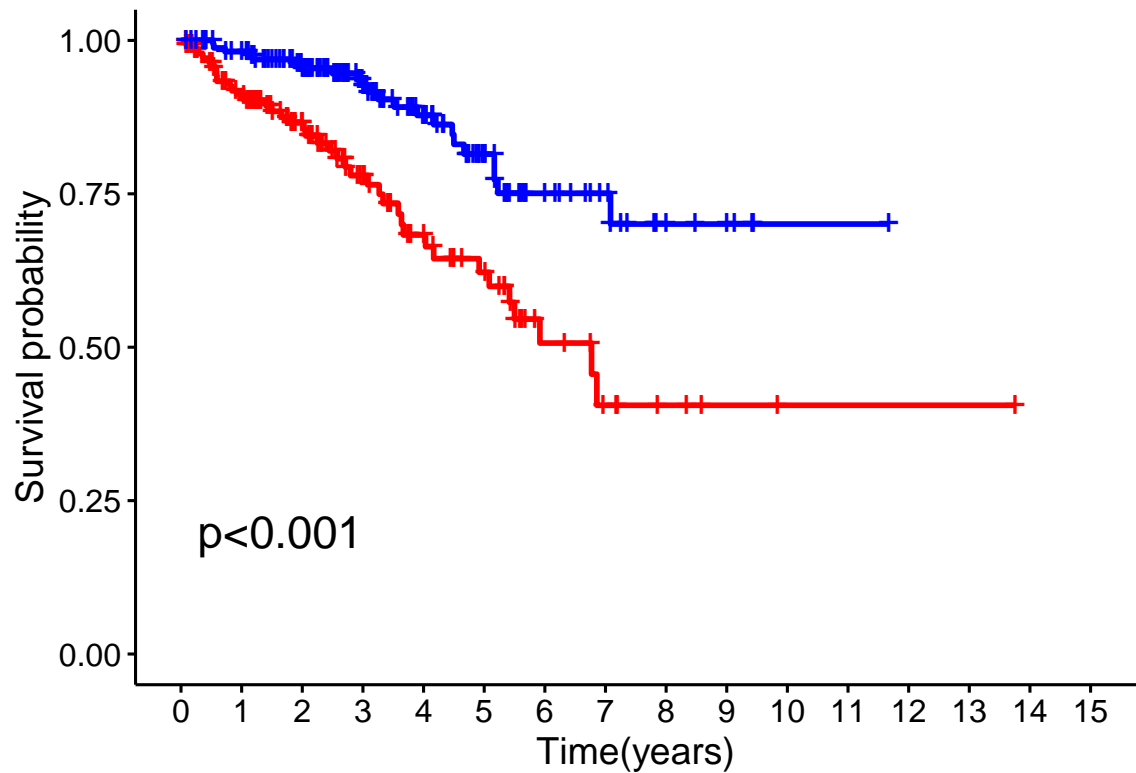

Supplement: Supplementary file 2 [file DataSheet1.ZIP › Source data for review purpose only/Source data/15.cliGroupSur/survival.SM_NO.pdf]

# Patients with YES

Risk + high + low

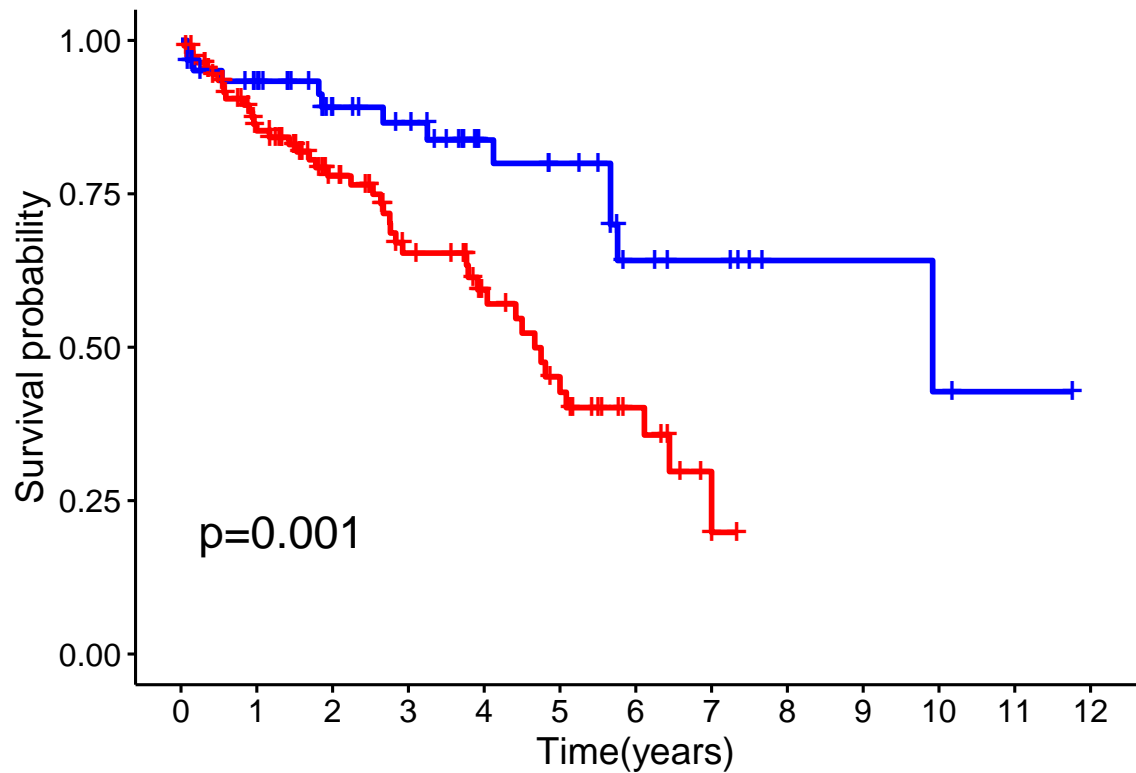

Supplement: Supplementary file 2 [file DataSheet1.ZIP › Source data for review purpose only/Source data/15.cliGroupSur/survival.SM_YES.pdf]

# Patients with T2

Risk + high + low

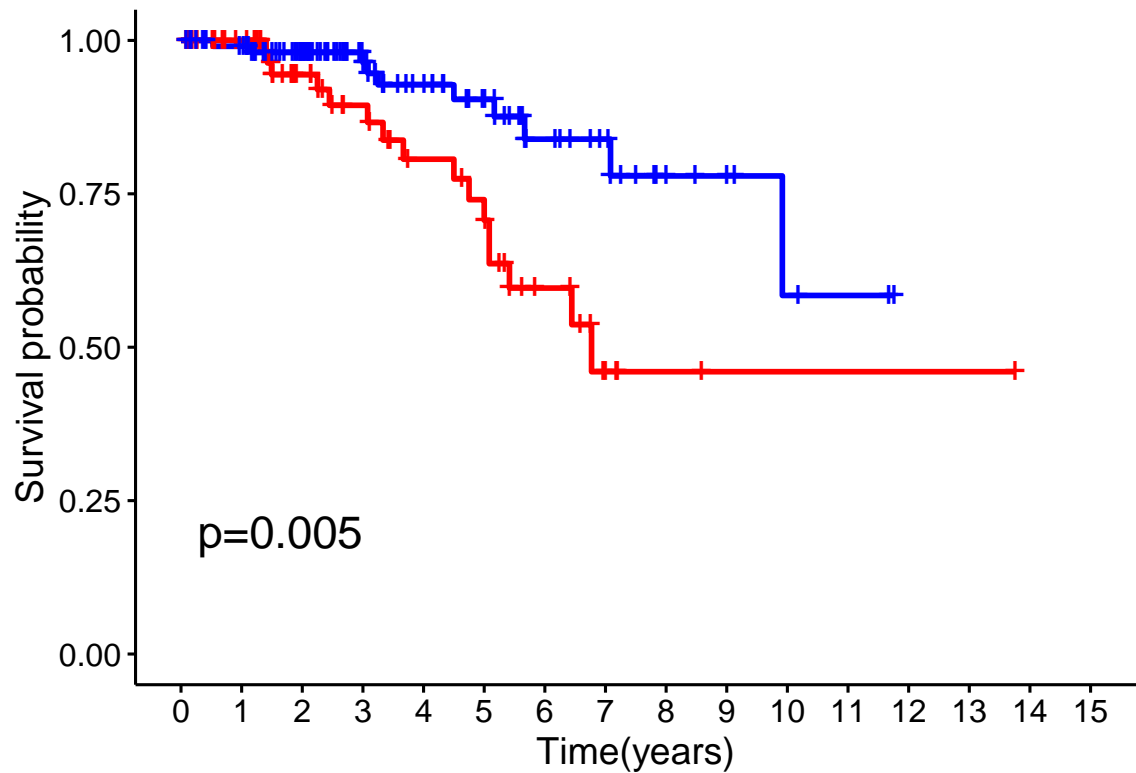

Supplement: Supplementary file 2 [file DataSheet1.ZIP › Source data for review purpose only/Source data/15.cliGroupSur/survival.T_T2.pdf]

# Patients with T3-T4

Risk + high + low

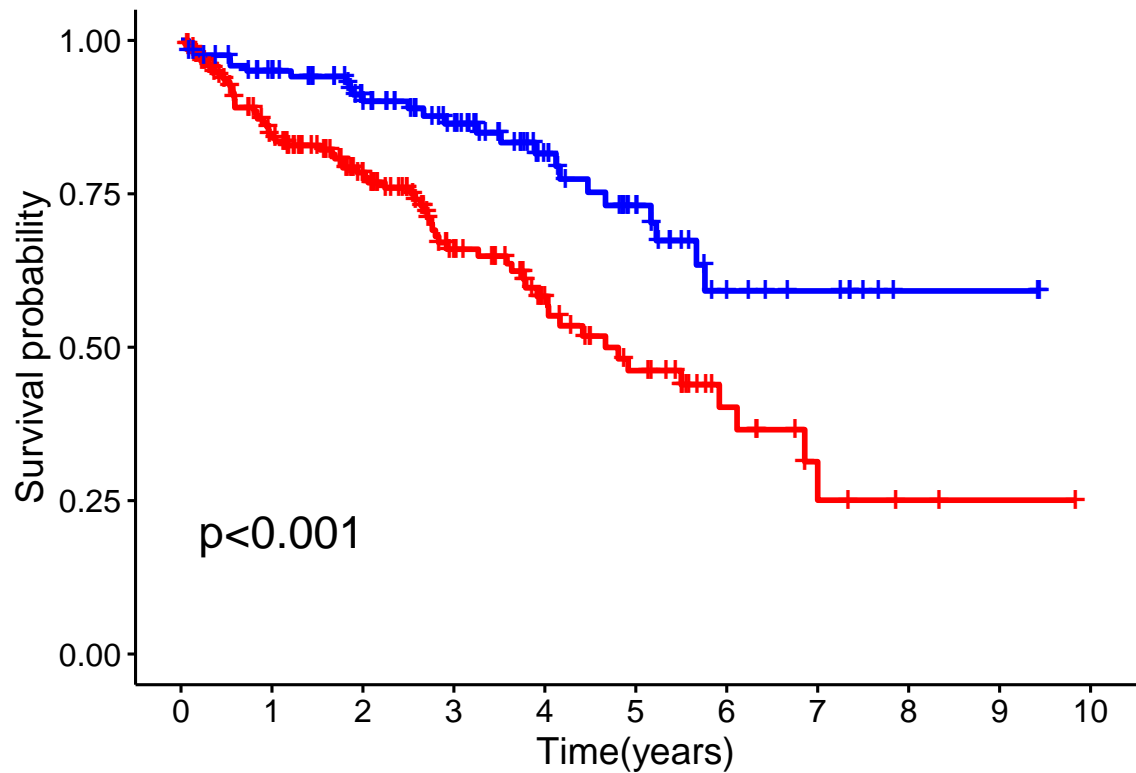

Supplement: Supplementary file 2 [file DataSheet1.ZIP › Source data for review purpose only/Source data/15.cliGroupSur/survival.T_T3-T4.pdf]

● Low risk ● High risk

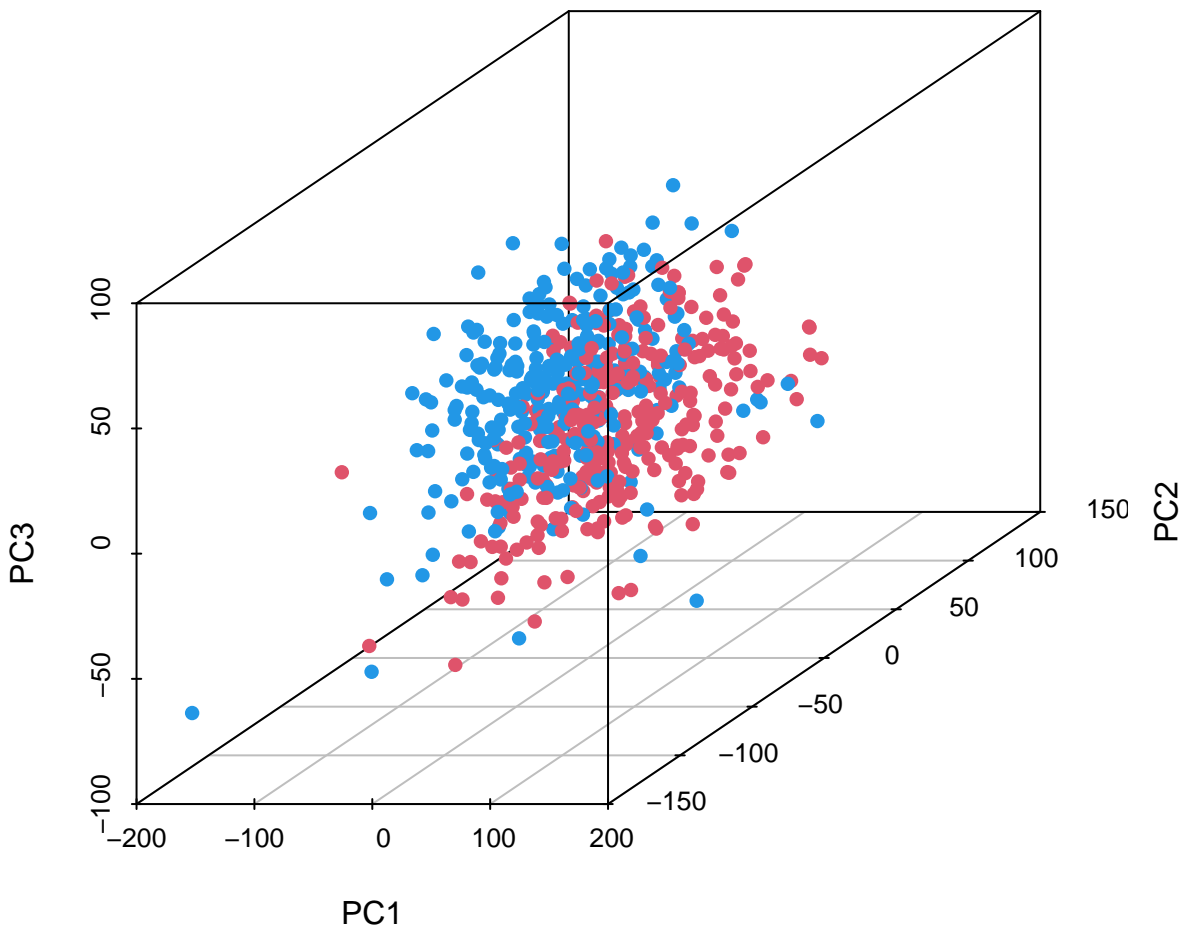

Supplement: Supplementary file 2 [file DataSheet1.ZIP › Source data for review purpose only/Source data/16.PCA/PCA.allGene.pdf]

● Low risk    ● High risk

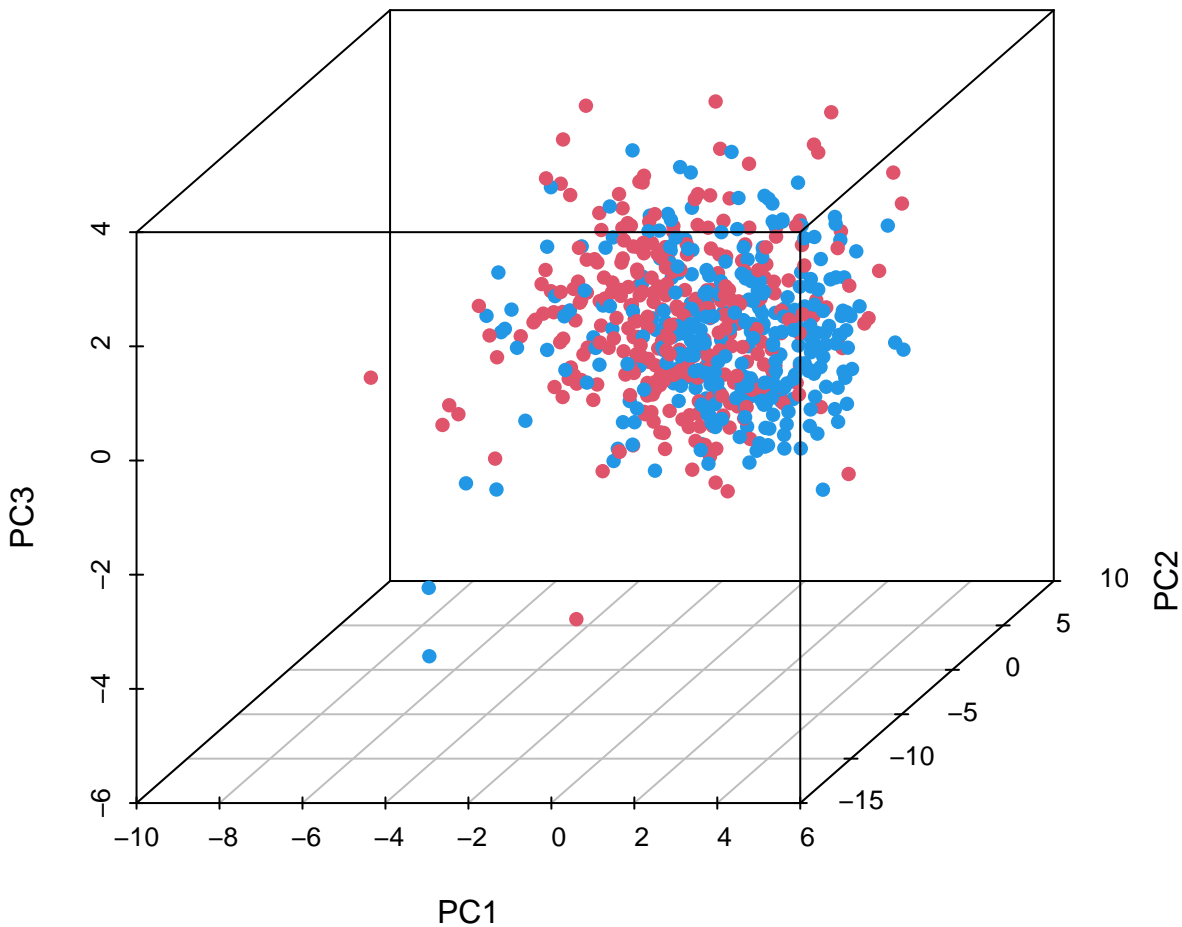

Supplement: Supplementary file 2 [file DataSheet1.ZIP › Source data for review purpose only/Source data/16.PCA/PCA.cuproptosisGene.pdf]

● Low risk ● High risk

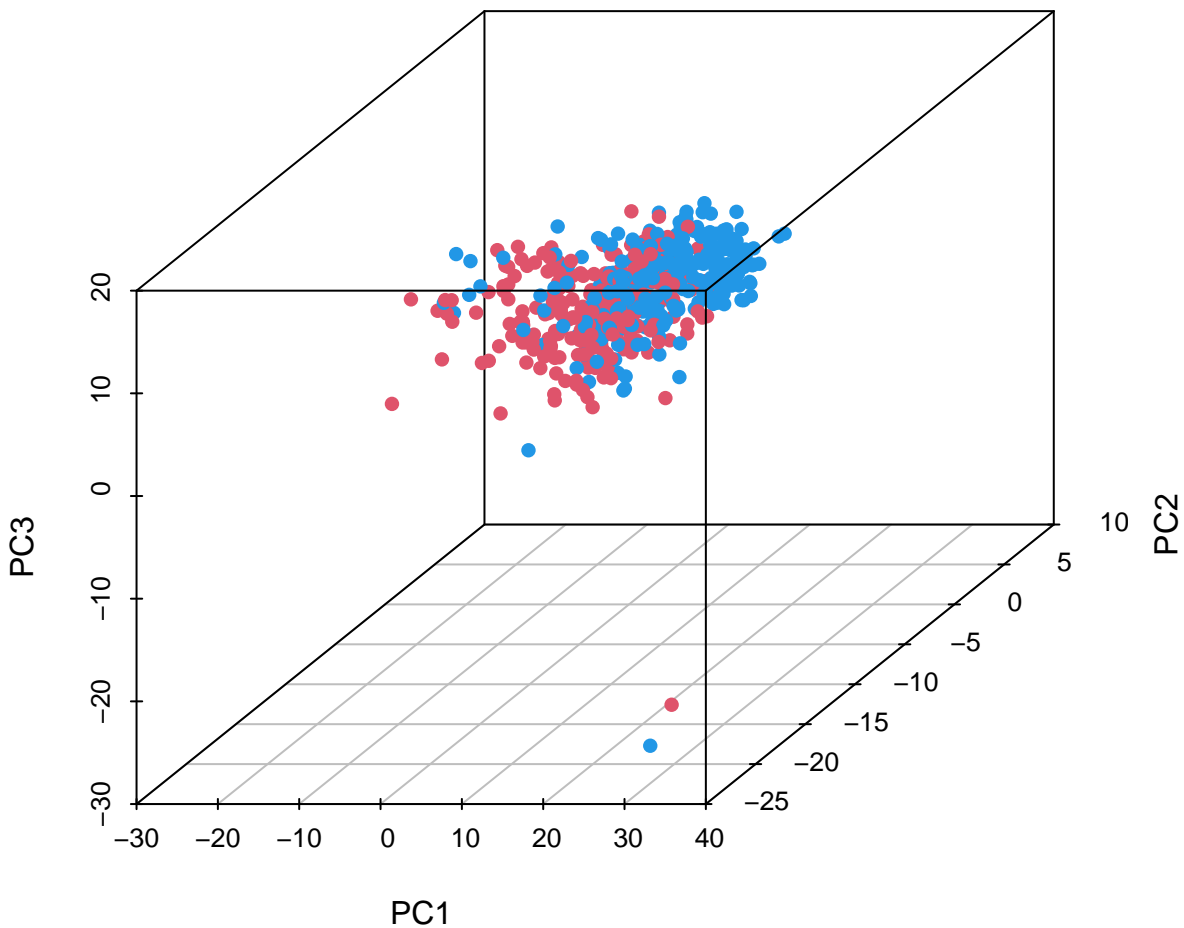

Supplement: Supplementary file 2 [file DataSheet1.ZIP › Source data for review purpose only/Source data/16.PCA/PCA.cuproptosisLncRNA.pdf]

● Low risk ● High risk

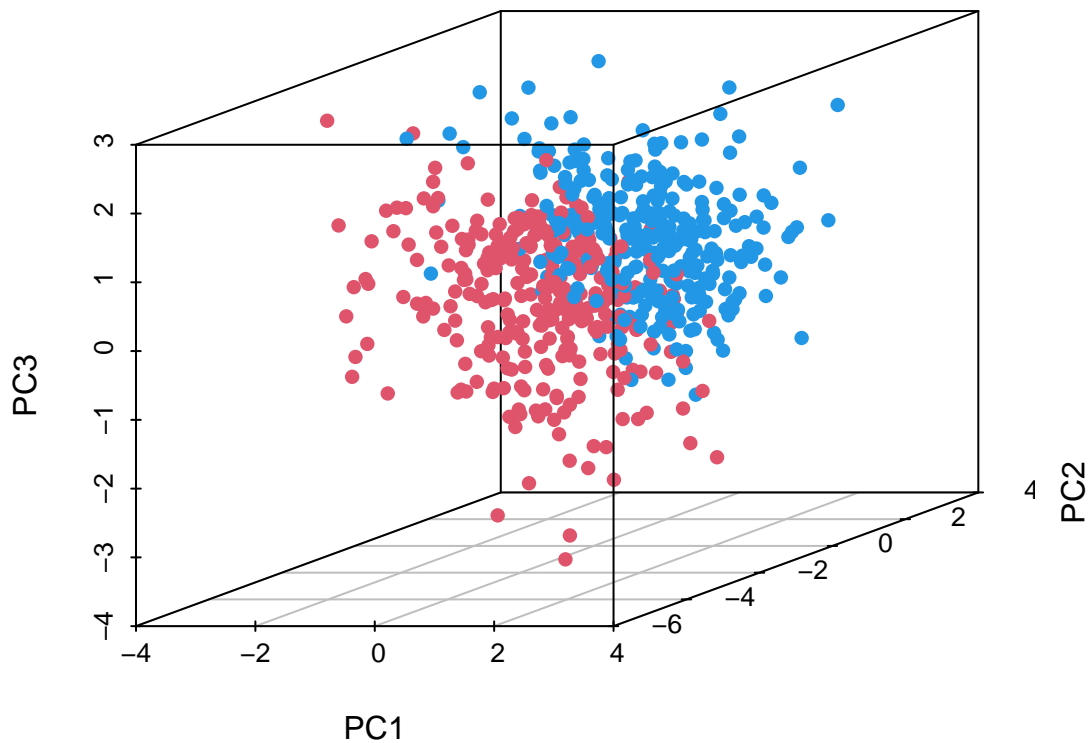

Supplement: Supplementary file 2 [file DataSheet1.ZIP › Source data for review purpose only/Source data/16.PCA/PCA.riskLnc.pdf]

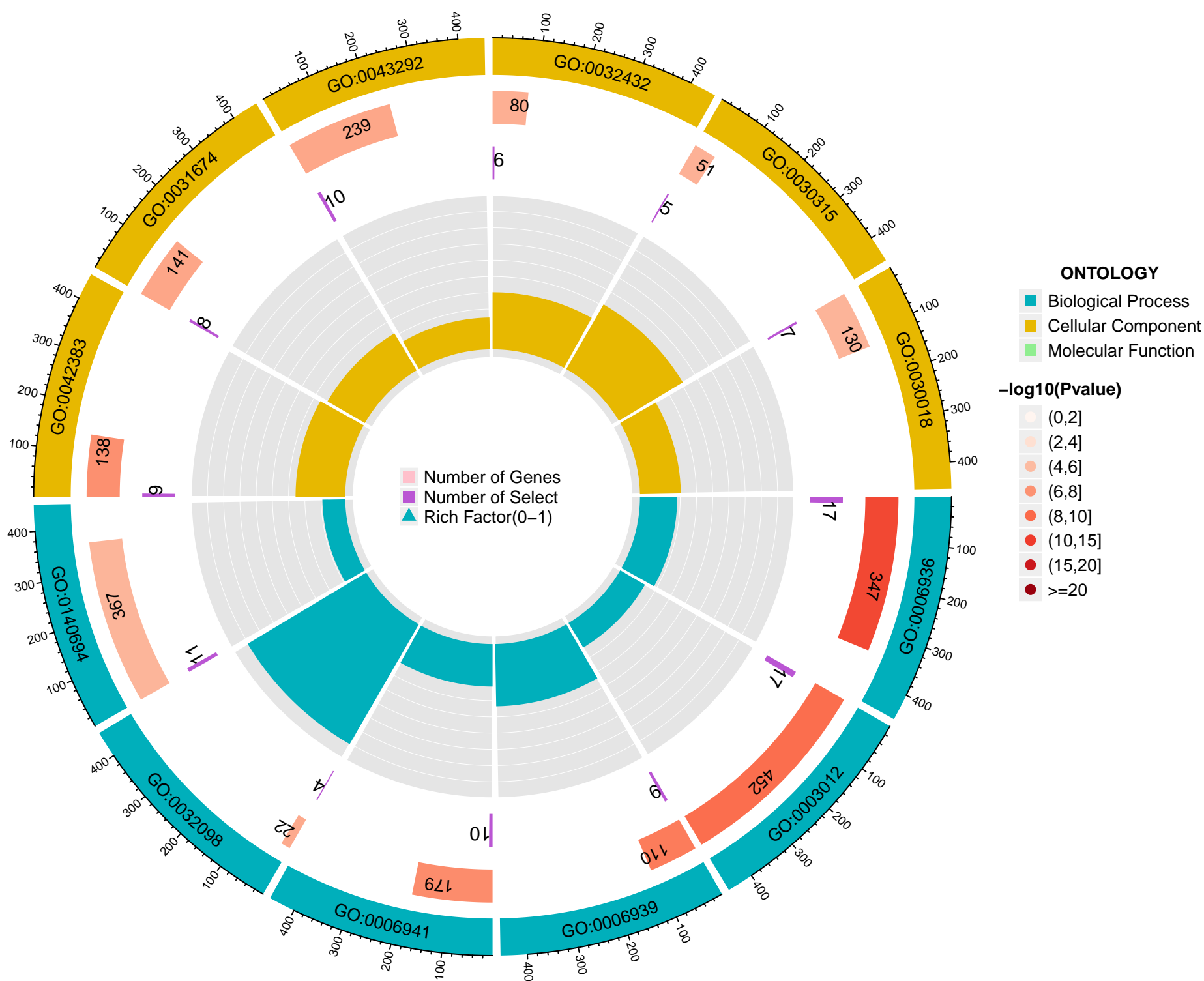

Supplement: Supplementary file 2 [file DataSheet1.ZIP › Source data for review purpose only/Source data/18.GO/GO.circlize.pdf]

Term

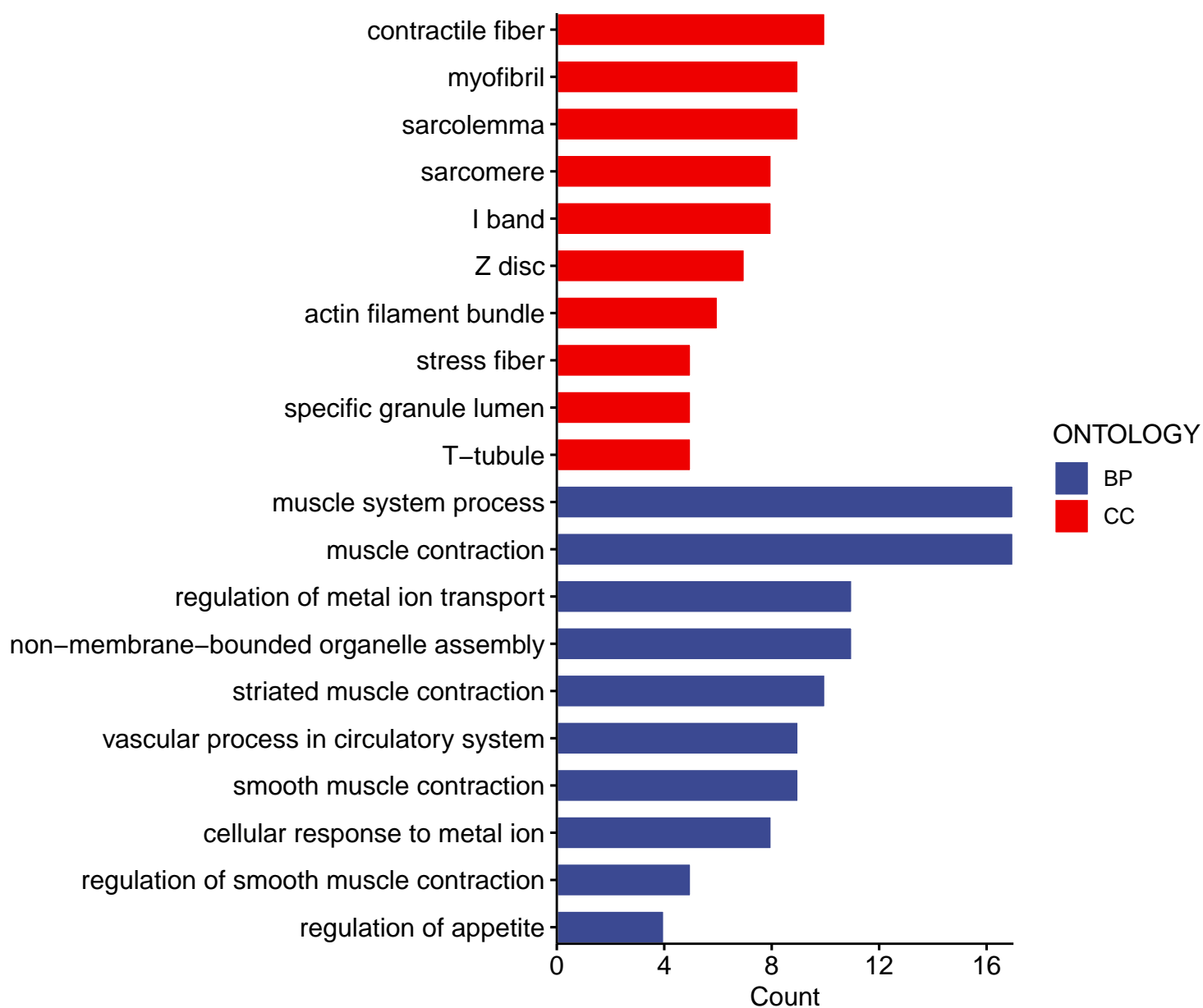

Supplement: Supplementary file 2 [file DataSheet1.ZIP › Source data for review purpose only/Source data/18.GO/barplot.color.pdf]

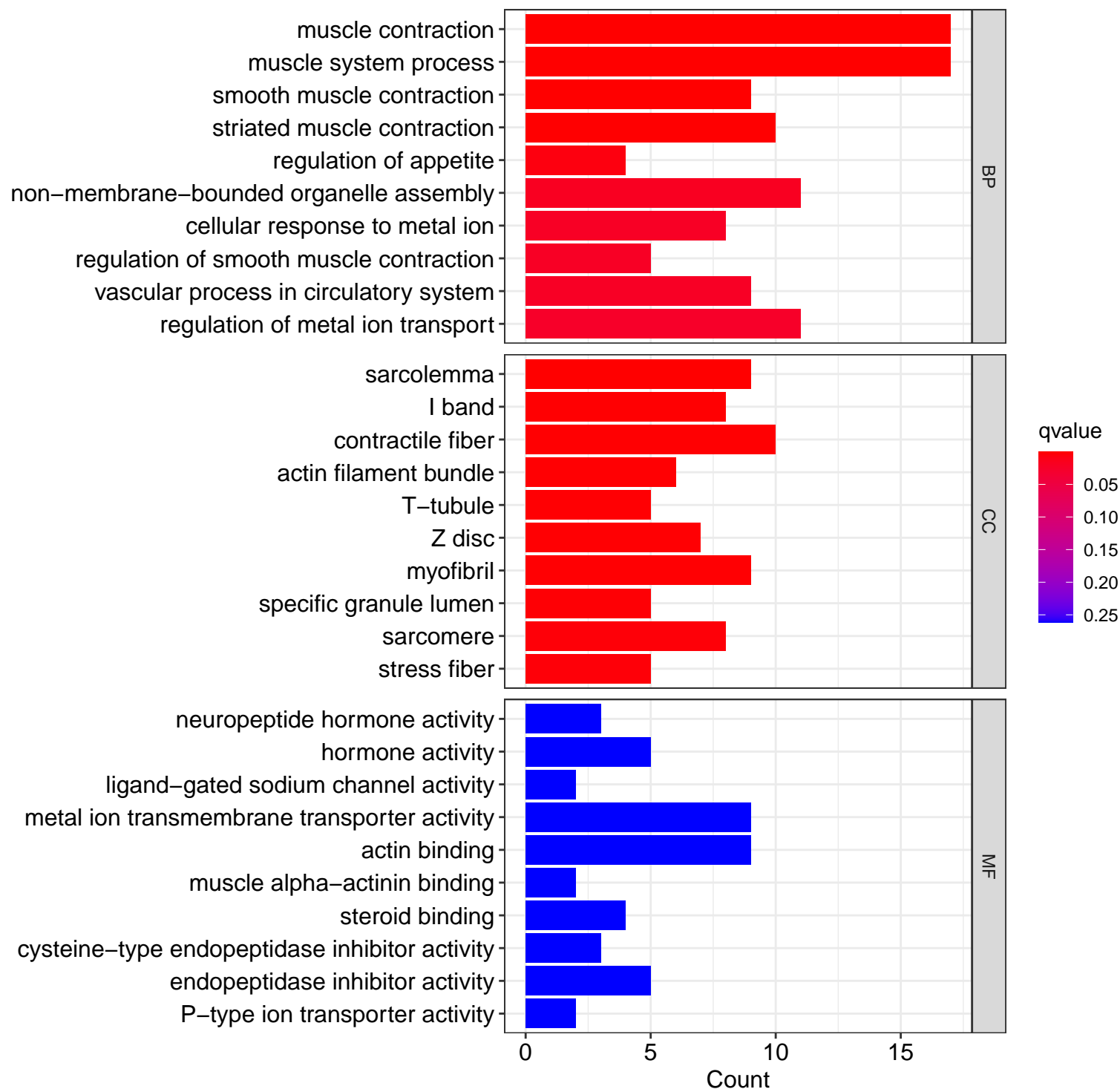

Supplement: Supplementary file 2 [file DataSheet1.ZIP › Source data for review purpose only/Source data/18.GO/barplot.pdf]

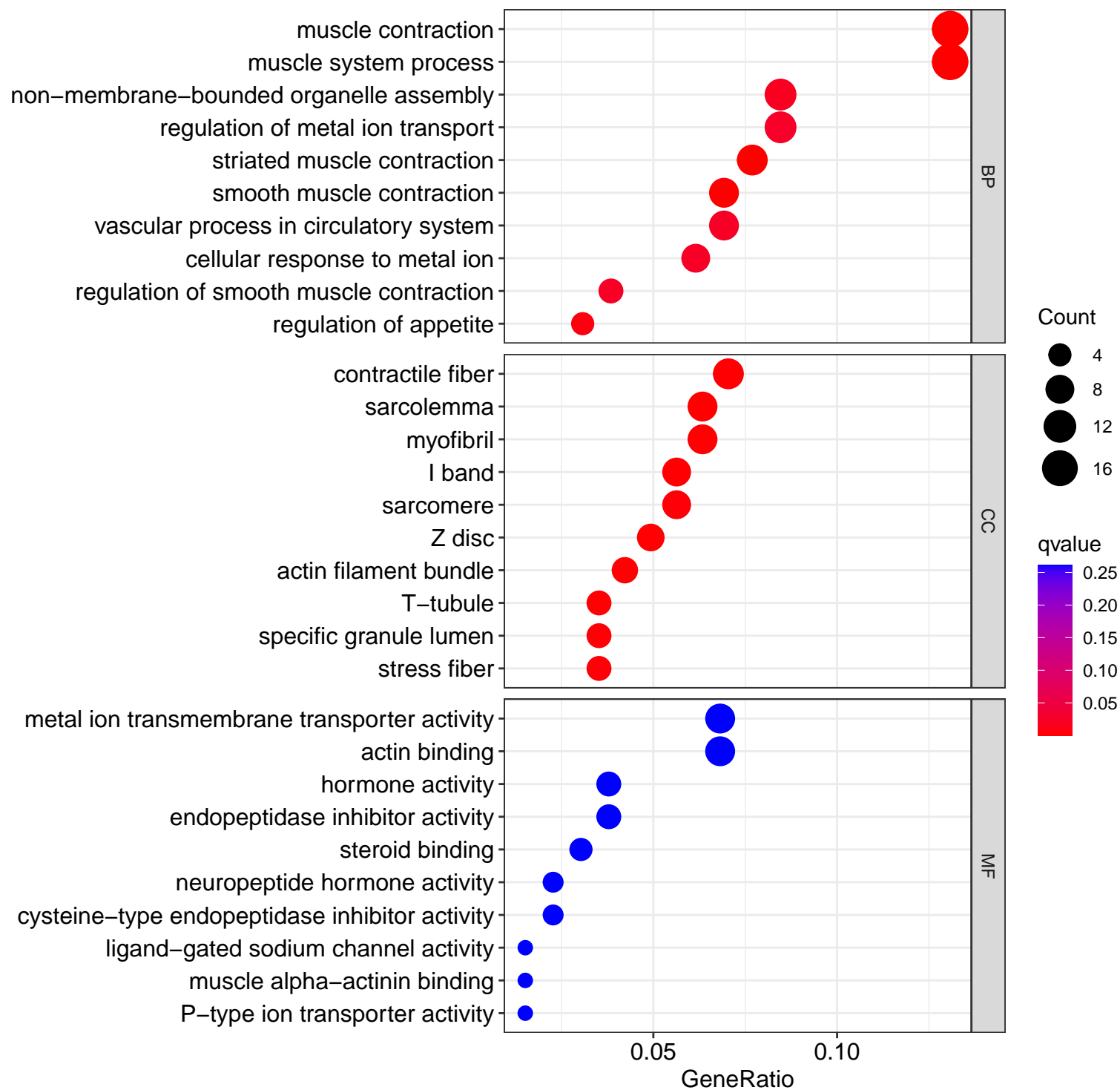

Supplement: Supplementary file 2 [file DataSheet1.ZIP › Source data for review purpose only/Source data/18.GO/bubble.pdf]

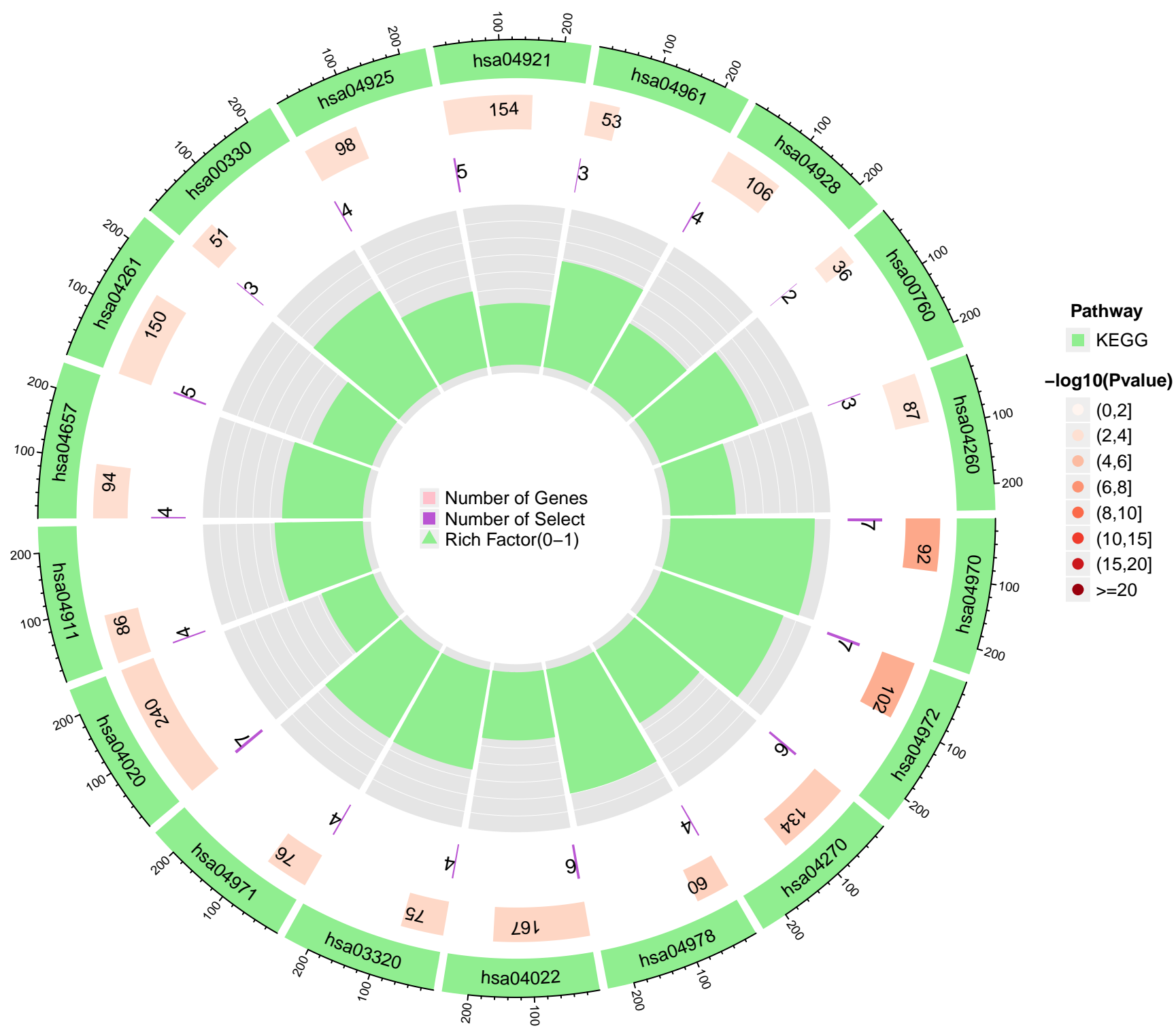

Supplement: Supplementary file 2 [file DataSheet1.ZIP › Source data for review purpose only/Source data/19.KEGG/KEGG.circlize.pdf]

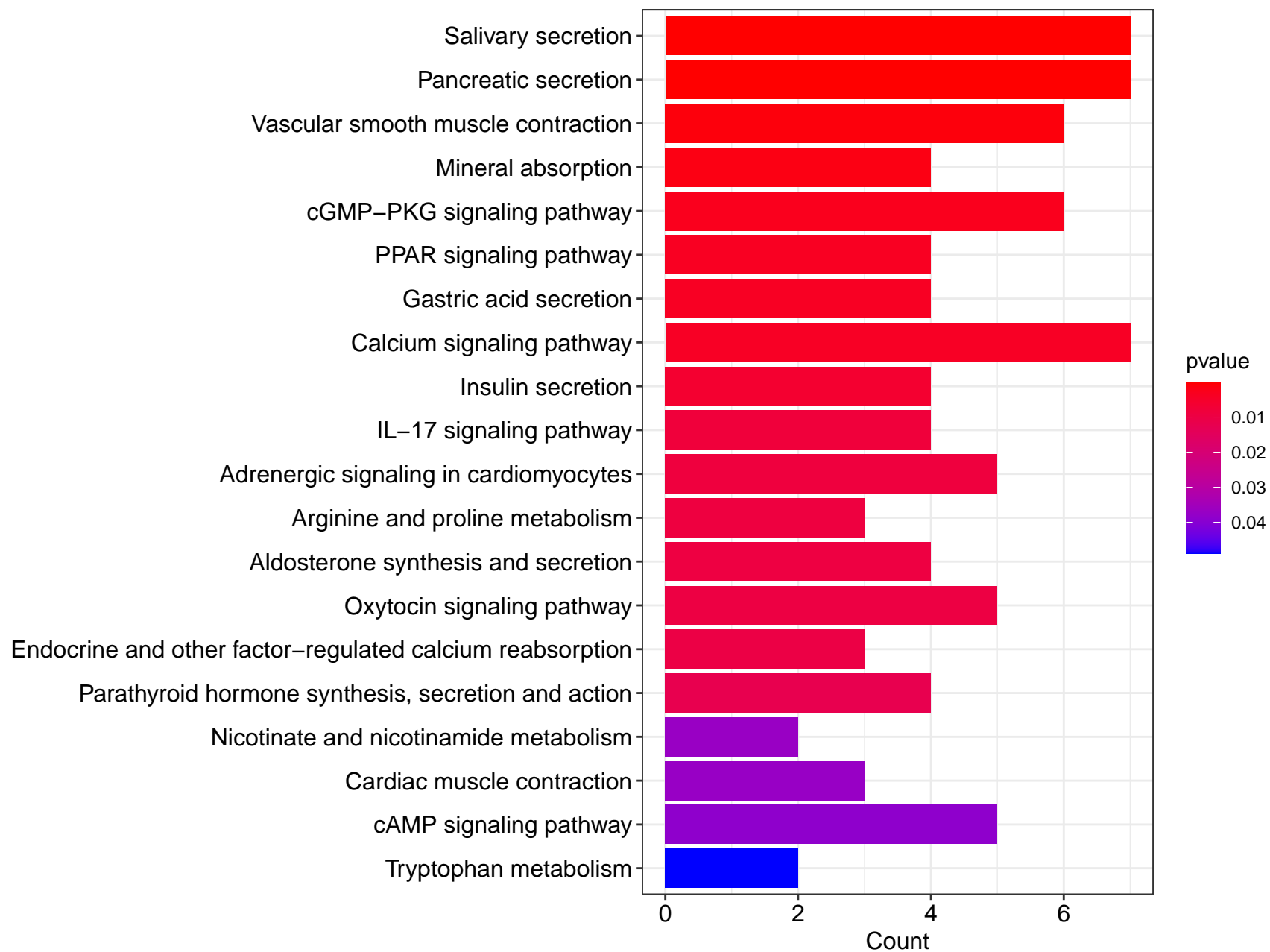

Supplement: Supplementary file 2 [file DataSheet1.ZIP › Source data for review purpose only/Source data/19.KEGG/barplot.pdf]

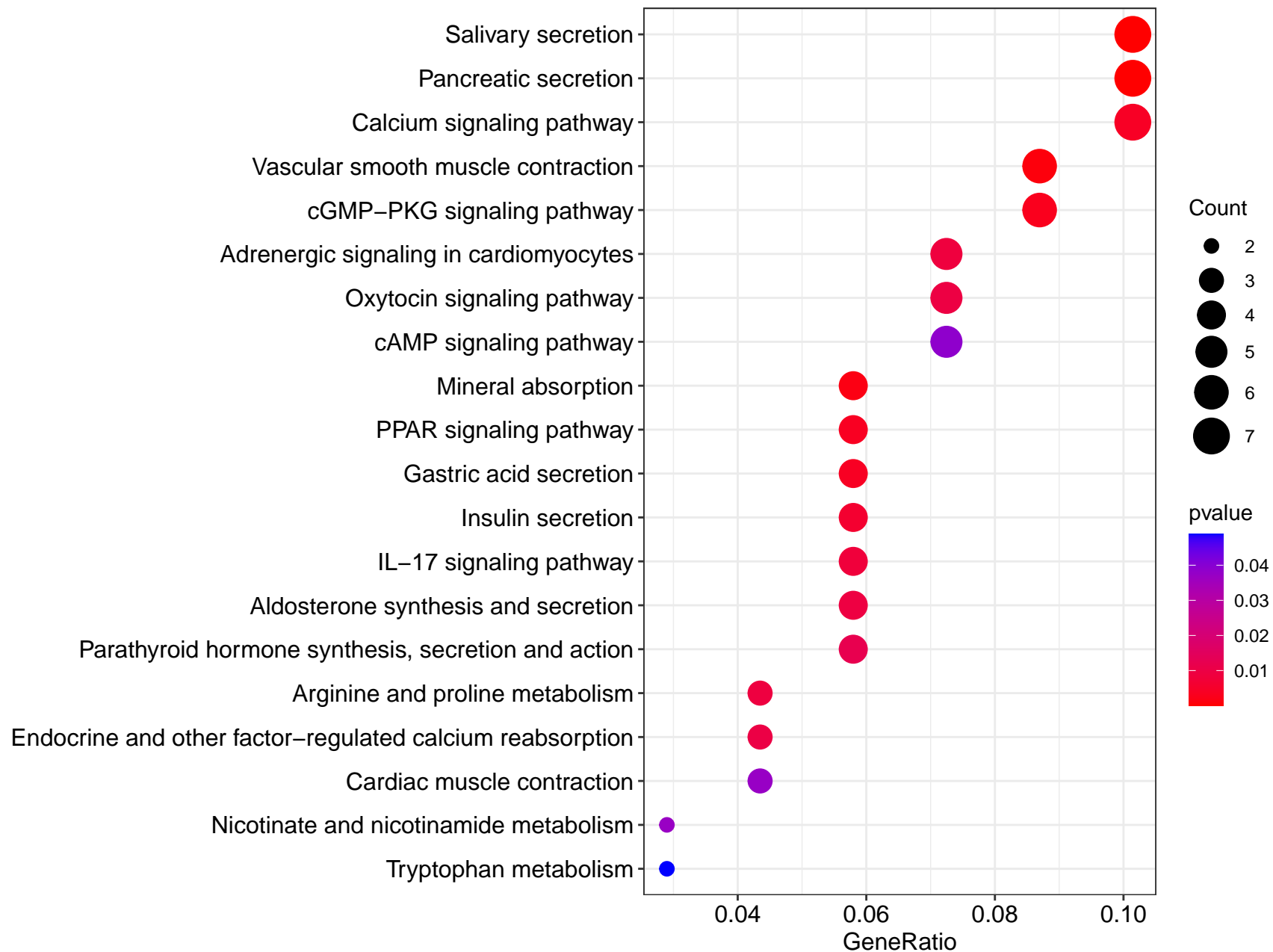

Supplement: Supplementary file 2 [file DataSheet1.ZIP › Source data for review purpose only/Source data/19.KEGG/bubble.pdf]

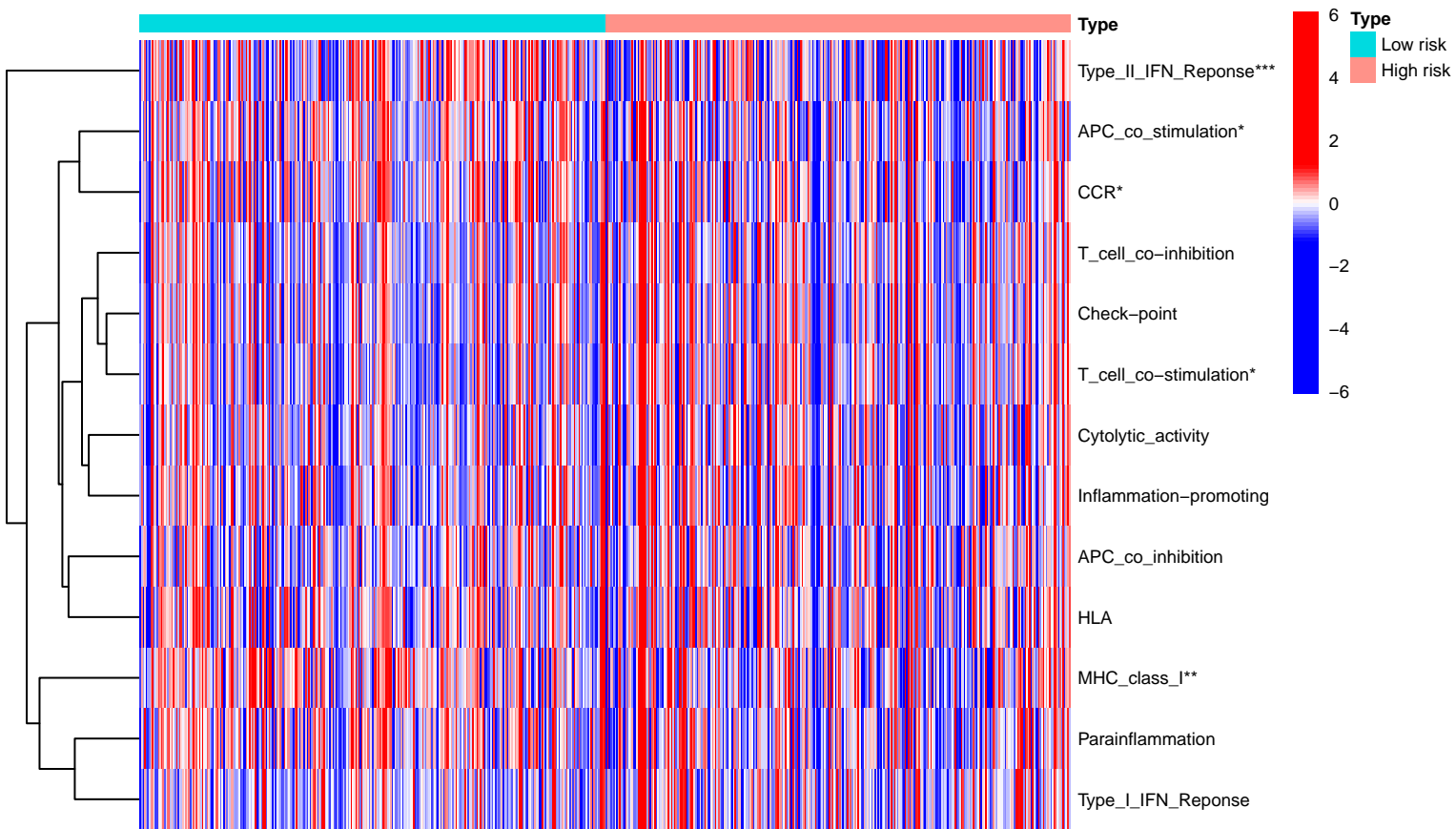

Supplement: Supplementary file 2 [file DataSheet1.ZIP › Source data for review purpose only/Source data/20.immFunction/heatmap.pdf]

Altered in 160 (65.31%) of 245 samples.

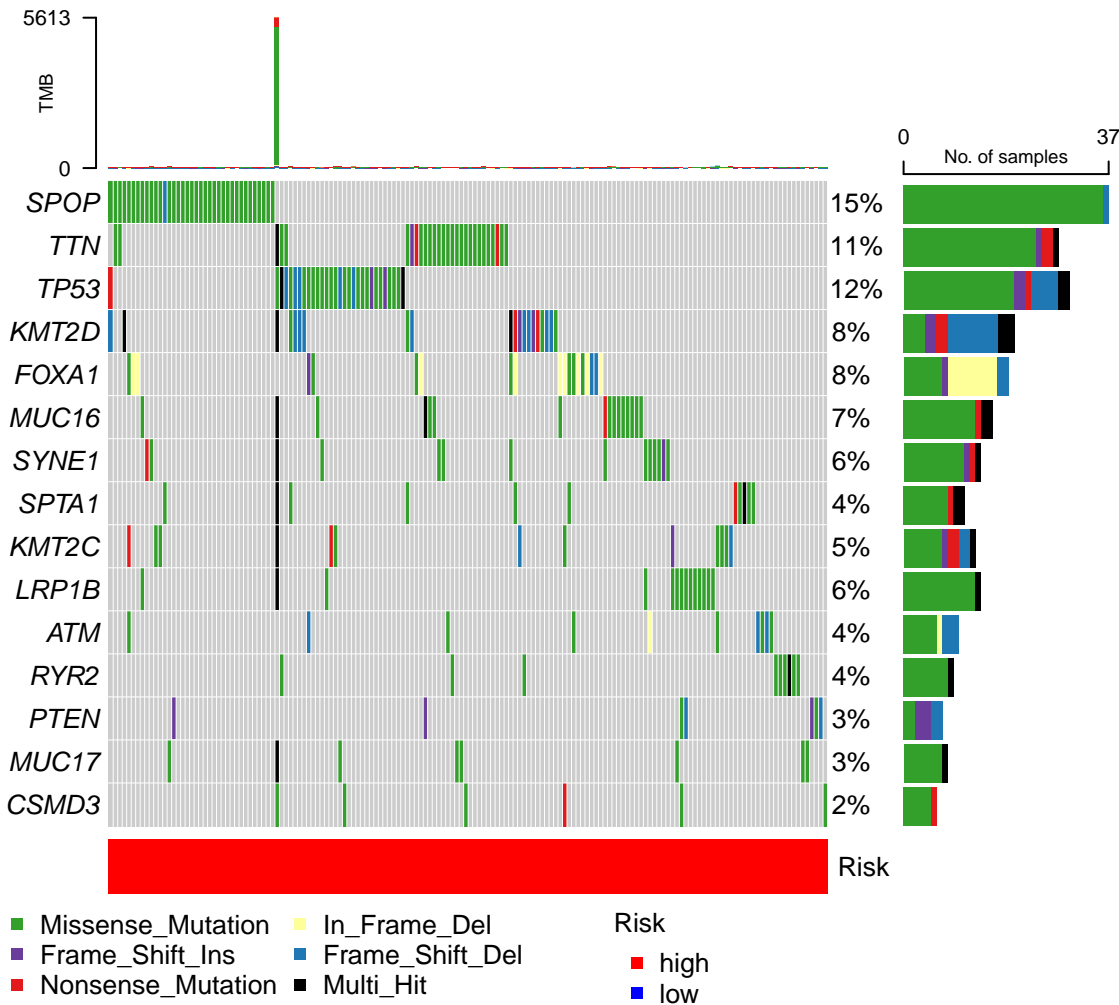

Supplement: Supplementary file 2 [file DataSheet1.ZIP › Source data for review purpose only/Source data/21.maftools/high.pdf]

Altered in 98 (43.36%) of 226 samples.

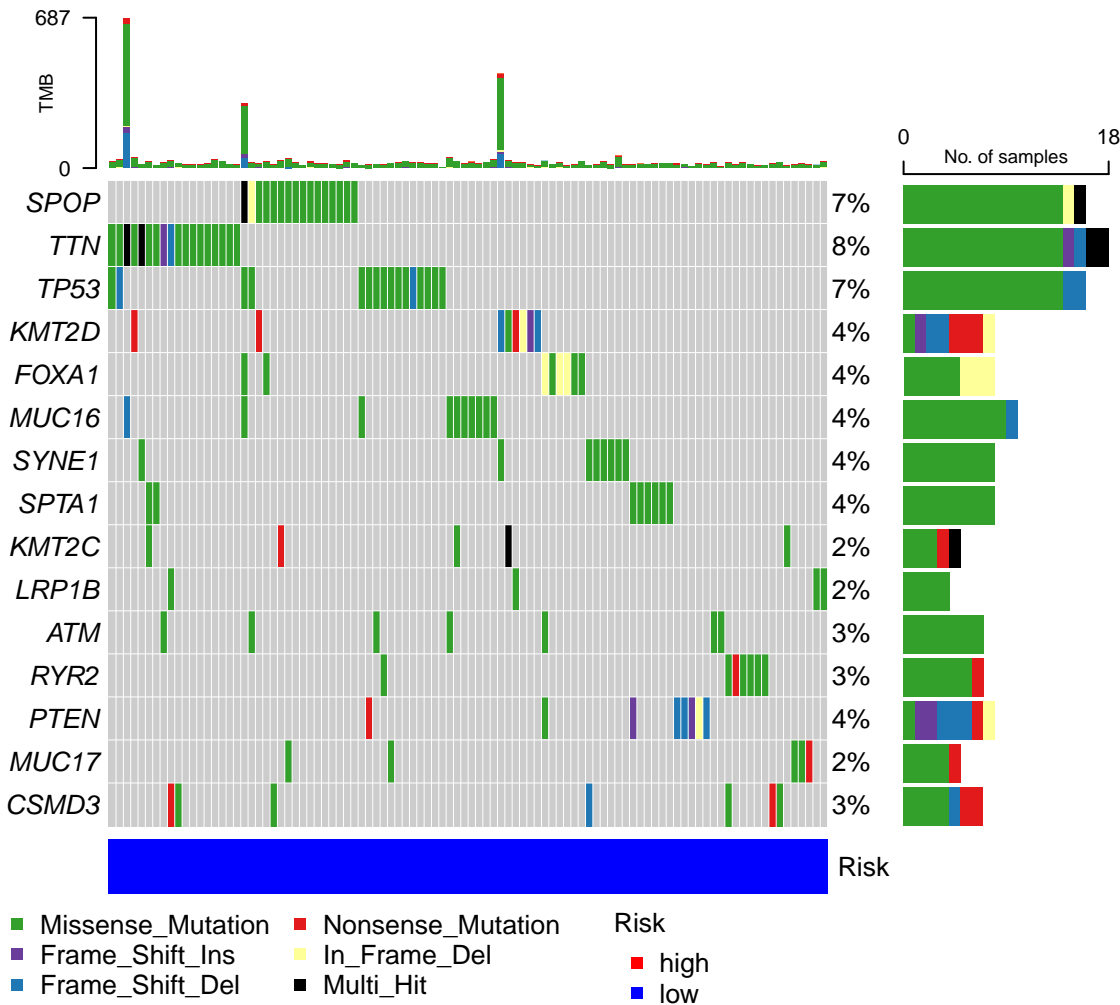

Supplement: Supplementary file 2 [file DataSheet1.ZIP › Source data for review purpose only/Source data/21.maftools/low.pdf]

Low-risk High-risk

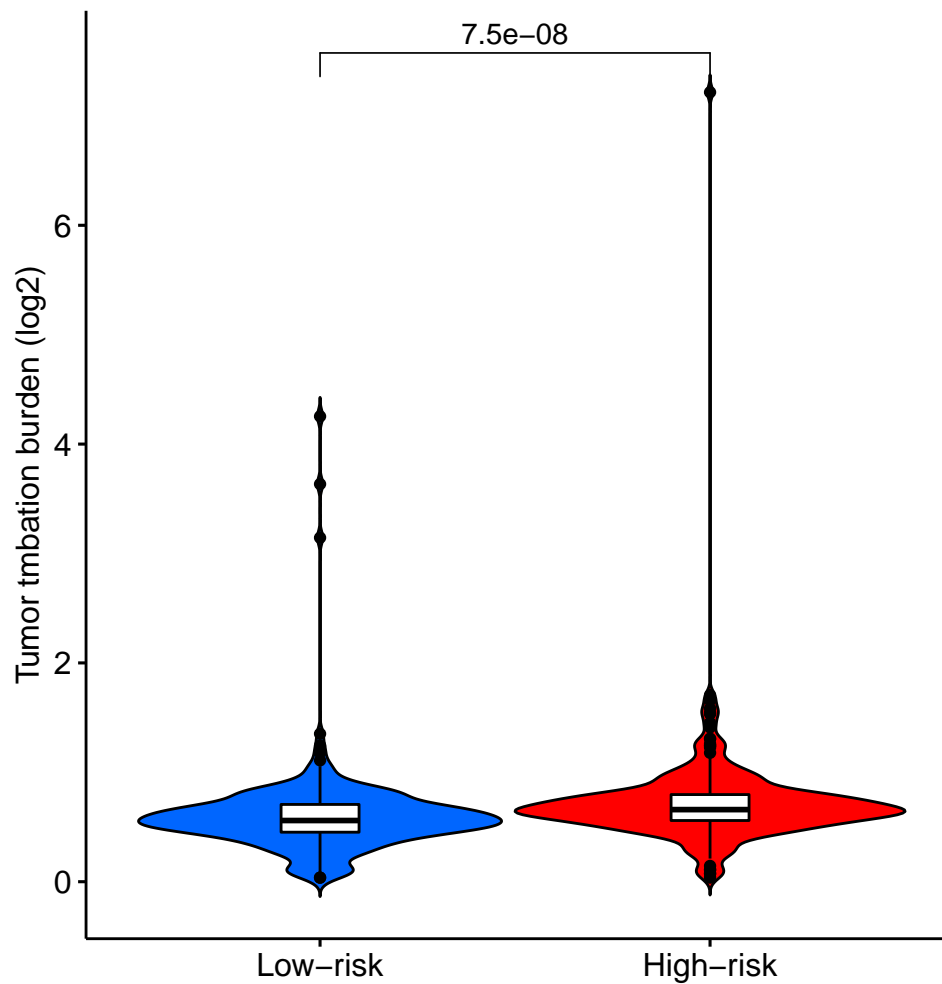

Supplement: Supplementary file 2 [file DataSheet1.ZIP › Source data for review purpose only/Source data/22.riskTMB/riskTMB.pdf]

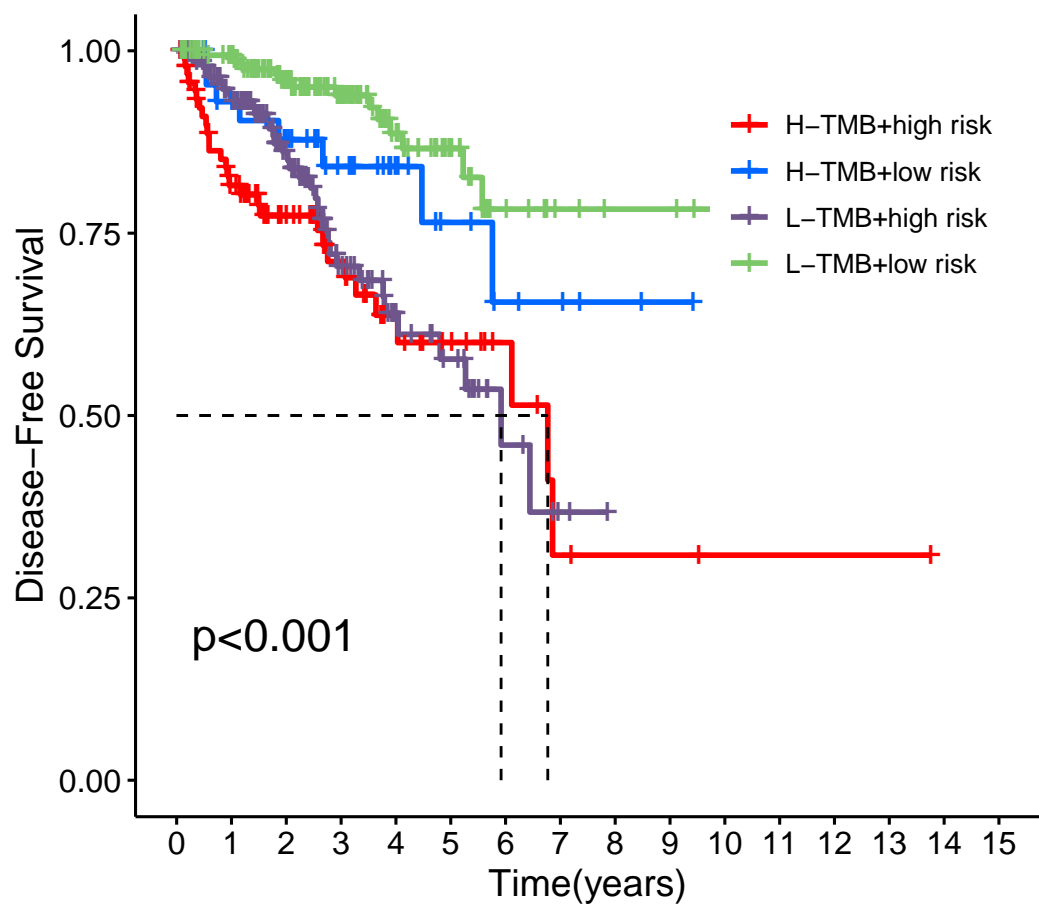

Supplement: Supplementary file 2 [file DataSheet1.ZIP › Source data for review purpose only/Source data/23.tmbSur/TMB-risk.survival.pdf]

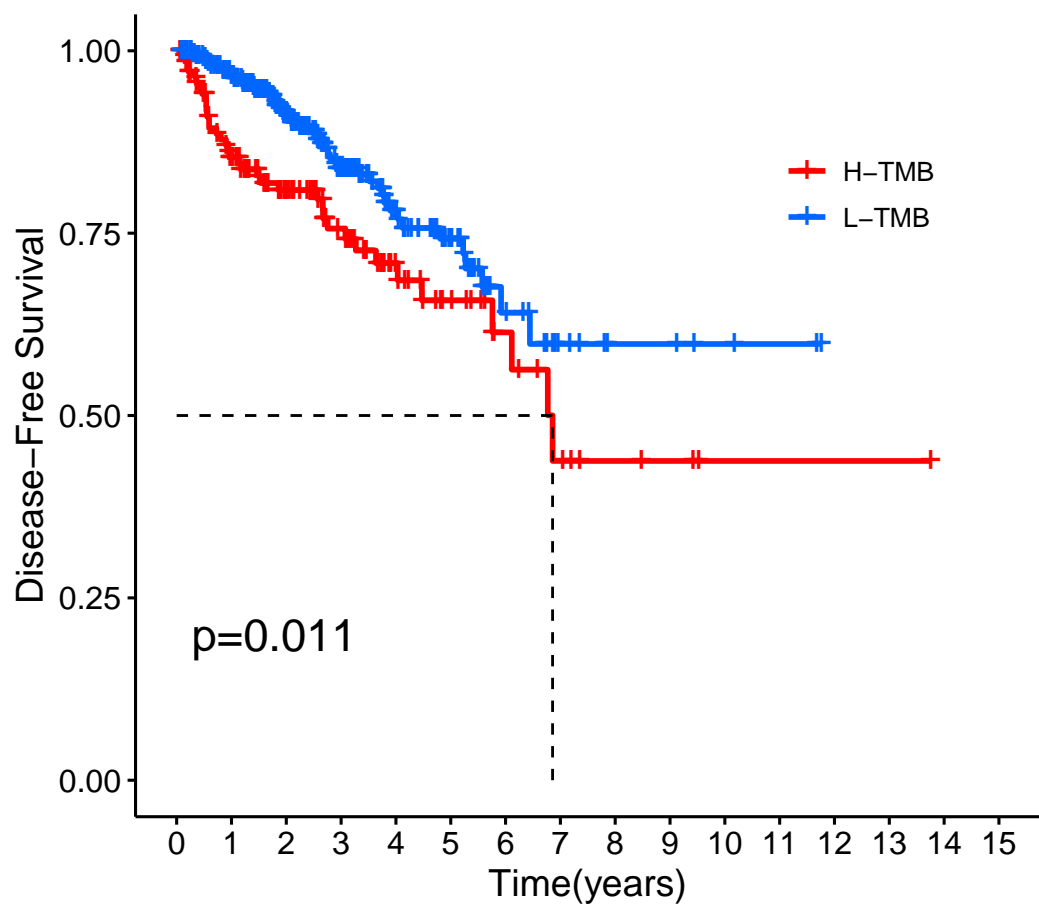

Supplement: Supplementary file 2 [file DataSheet1.ZIP › Source data for review purpose only/Source data/23.tmbSur/TMB.survival.pdf]

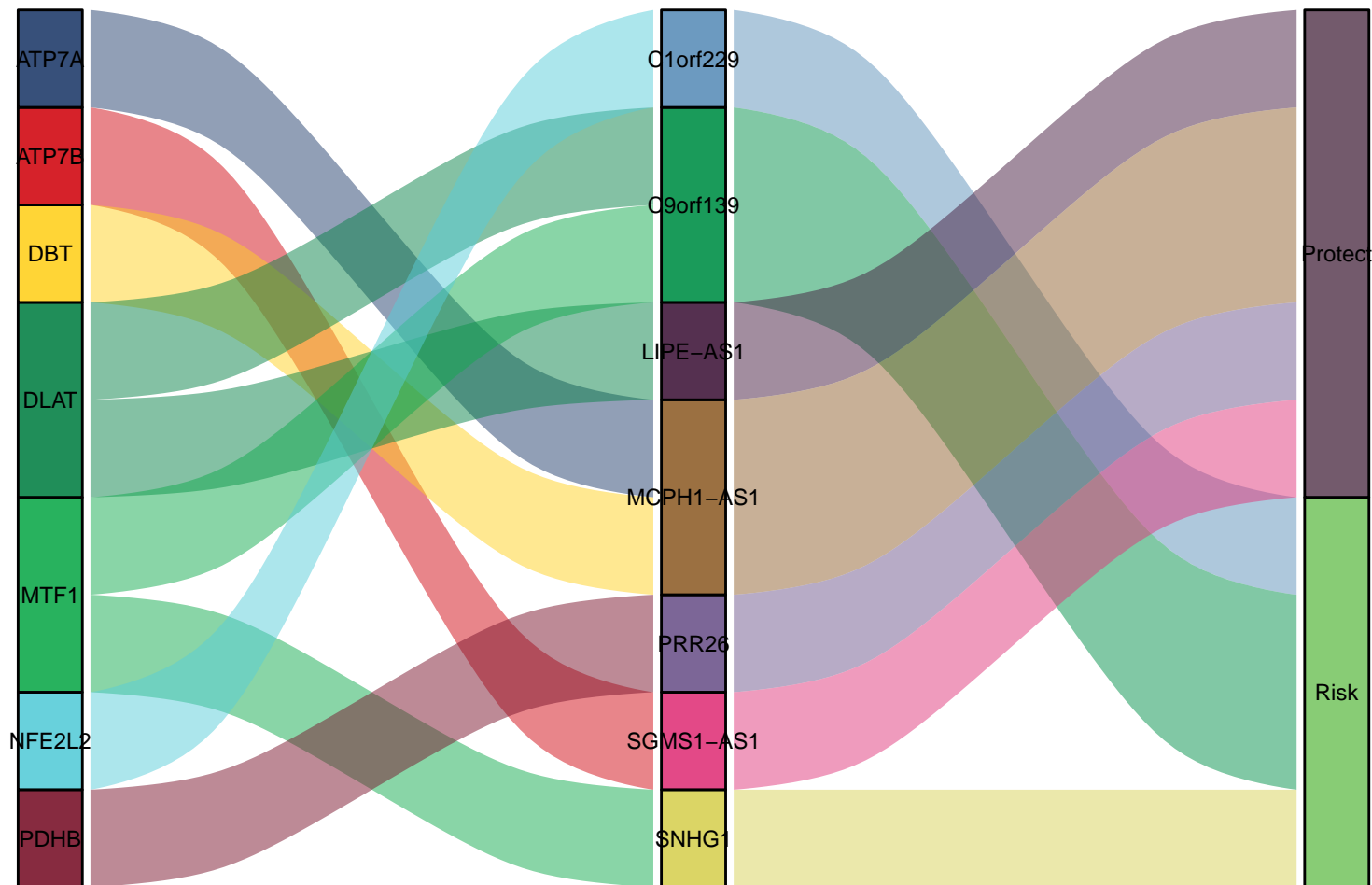

Supplement: Supplementary file 2 [file DataSheet1.ZIP › Source data for review purpose only/Source data/24.Sankey/Sankey.pdf]

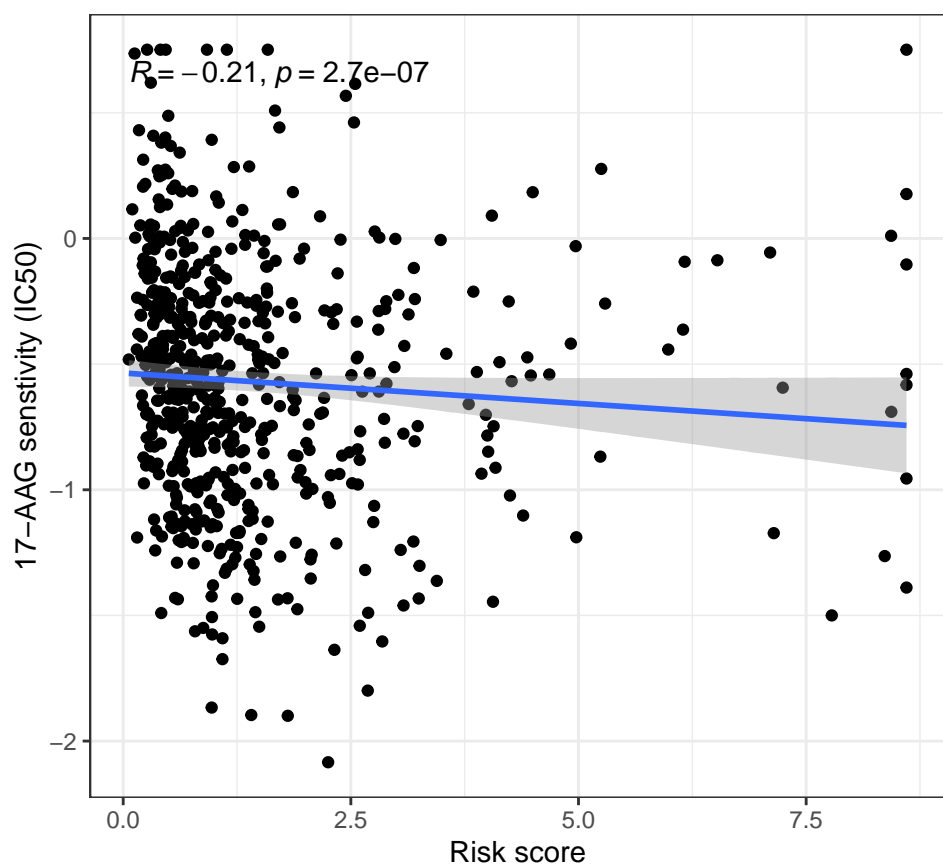

Supplement: Supplementary file 2 [file DataSheet1.ZIP › Source data for review purpose only/Source data/25.pRRophetic/Cor.17-AAG.pdf]

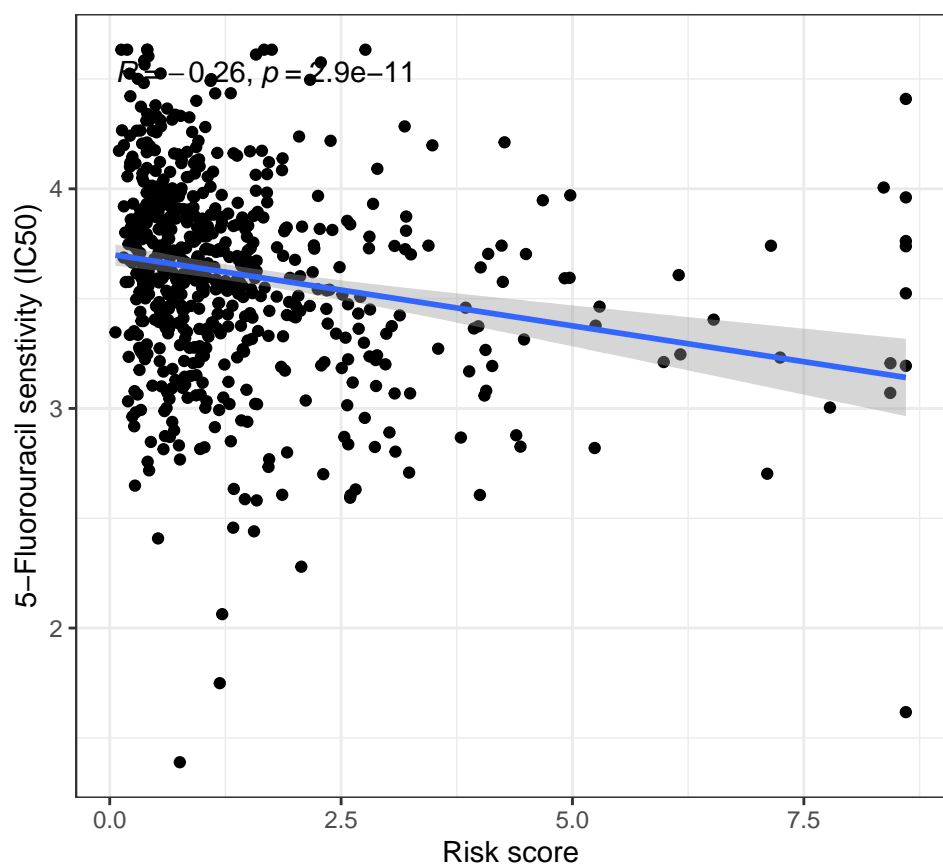

Supplement: Supplementary file 2 [file DataSheet1.ZIP › Source data for review purpose only/Source data/25.pRRophetic/Cor.5-Fluorouracil.pdf]

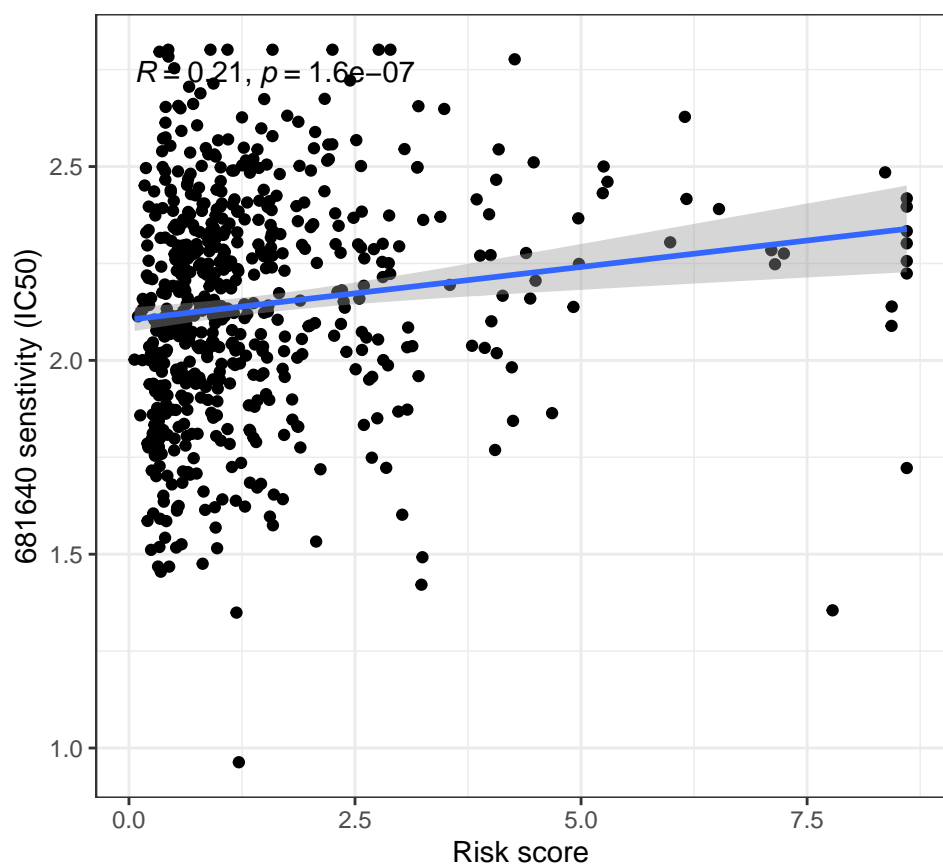

Supplement: Supplementary file 2 [file DataSheet1.ZIP › Source data for review purpose only/Source data/25.pRRophetic/Cor.681640.pdf]

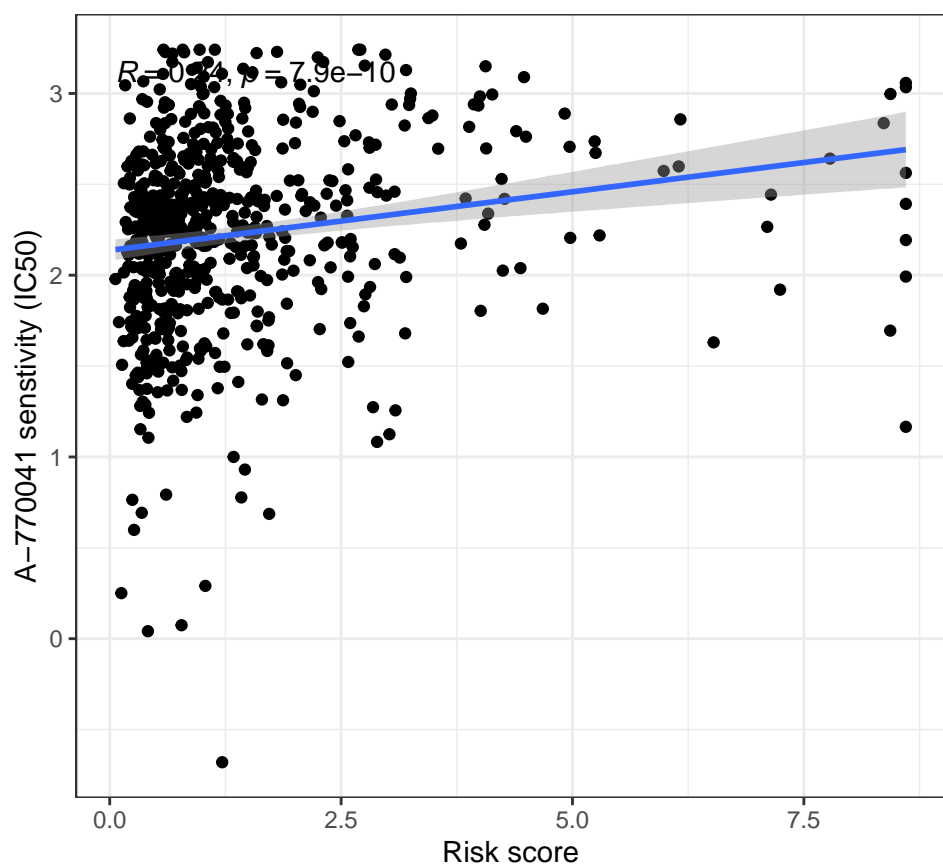

Supplement: Supplementary file 2 [file DataSheet1.ZIP › Source data for review purpose only/Source data/25.pRRophetic/Cor.A-770041.pdf]

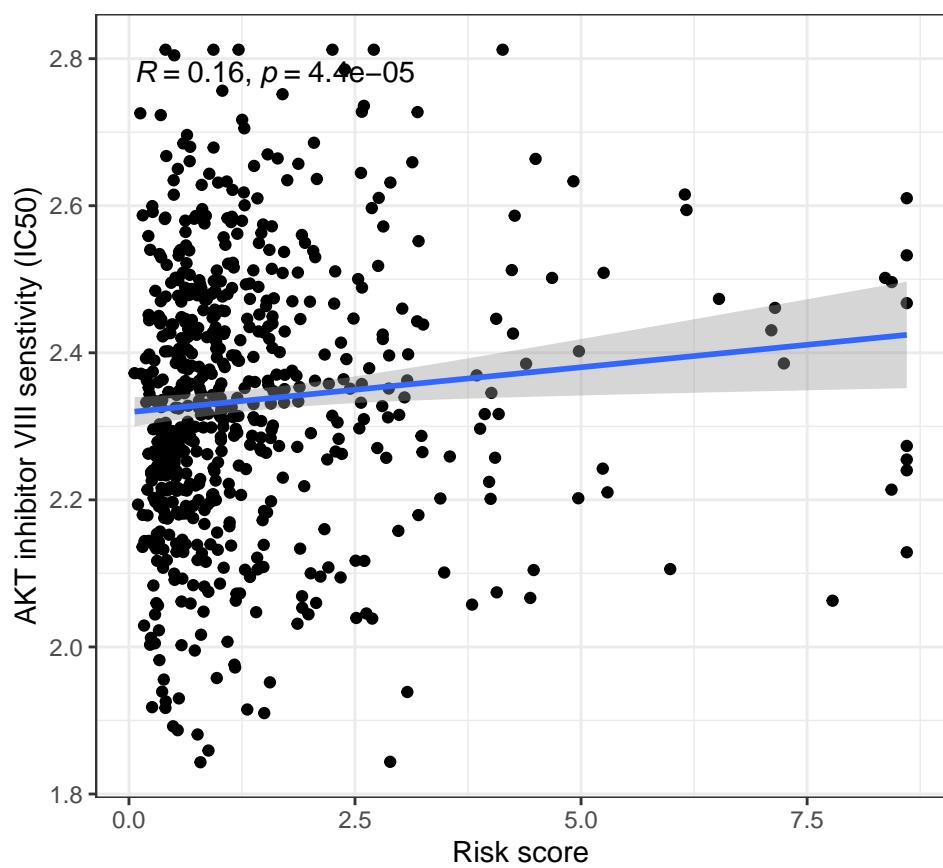

Supplement: Supplementary file 2 [file DataSheet1.ZIP › Source data for review purpose only/Source data/25.pRRophetic/Cor.AKT inhibitor VIII.pdf]

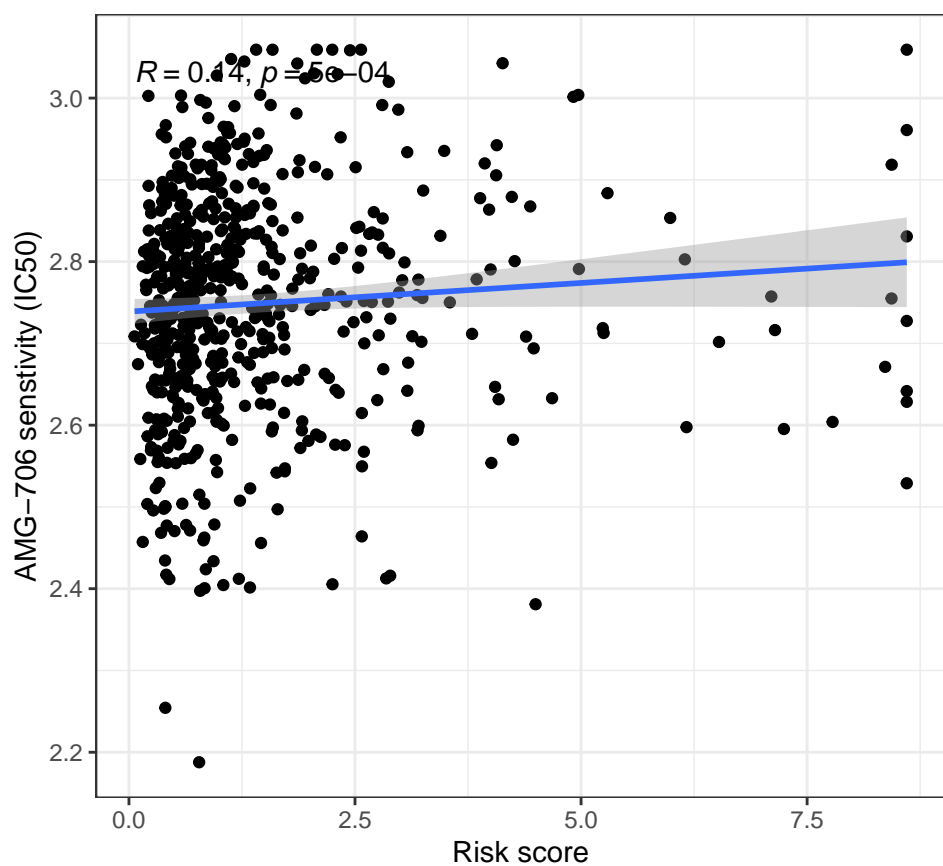

Supplement: Supplementary file 2 [file DataSheet1.ZIP › Source data for review purpose only/Source data/25.pRRophetic/Cor.AMG-706.pdf]

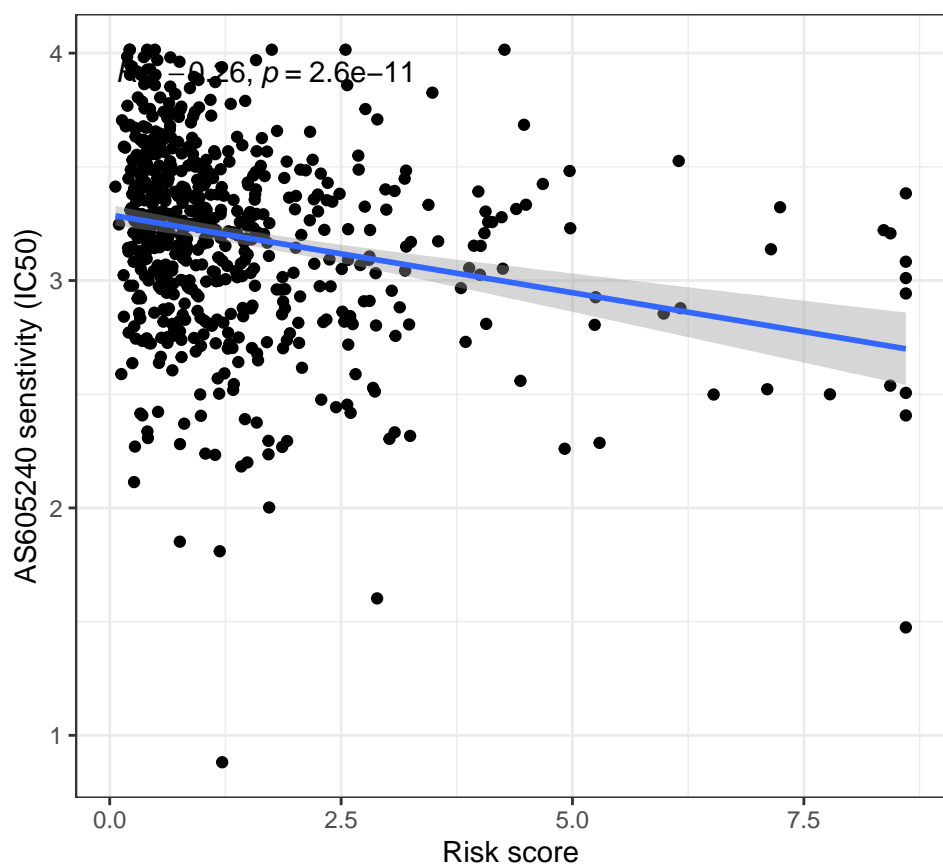

Supplement: Supplementary file 2 [file DataSheet1.ZIP › Source data for review purpose only/Source data/25.pRRophetic/Cor.AS605240.pdf]

AU922 sensitivity (IC50)

$R^2 = -0.21, p = 1.3e-07$

0.0 2.5 5.0 7.5

Risk score

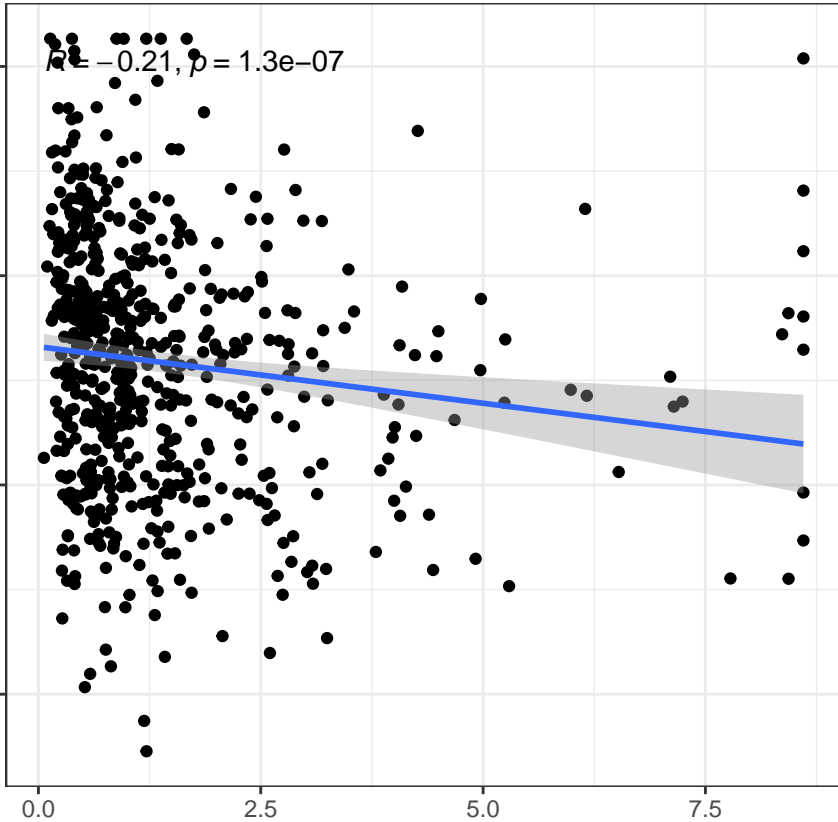

Supplement: Supplementary file 2 [file DataSheet1.ZIP › Source data for review purpose only/Source data/25.pRRophetic/Cor.AUY922.pdf]

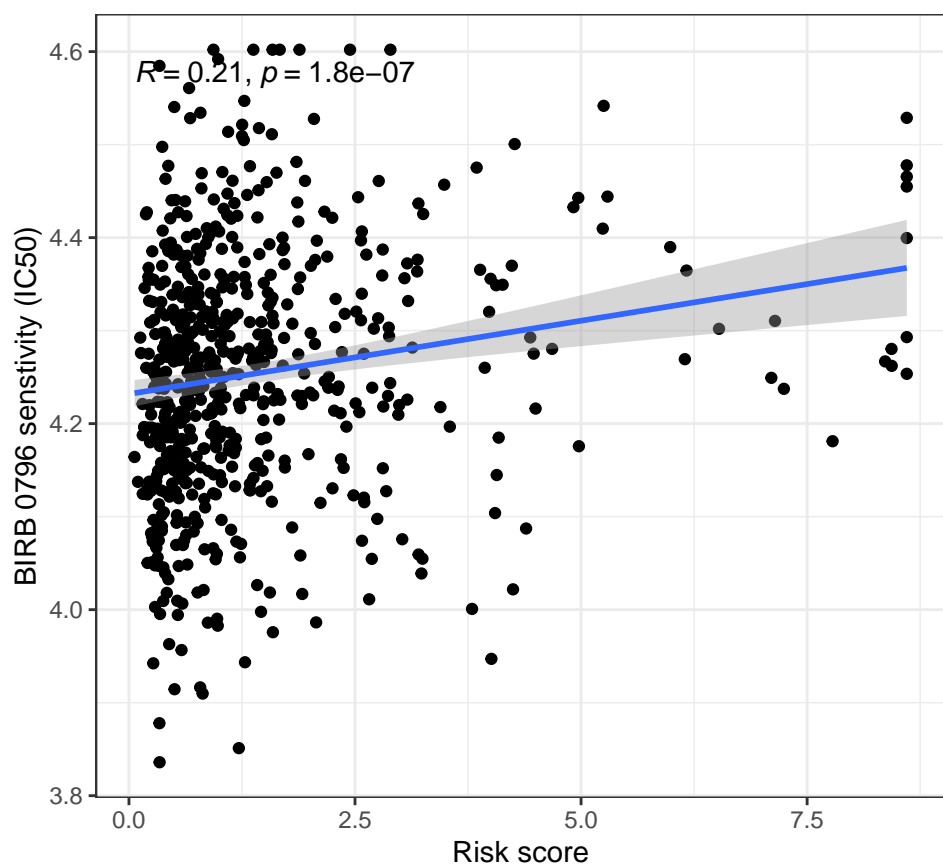

Supplement: Supplementary file 2 [file DataSheet1.ZIP › Source data for review purpose only/Source data/25.pRRophetic/Cor.BIRB 0796.pdf]

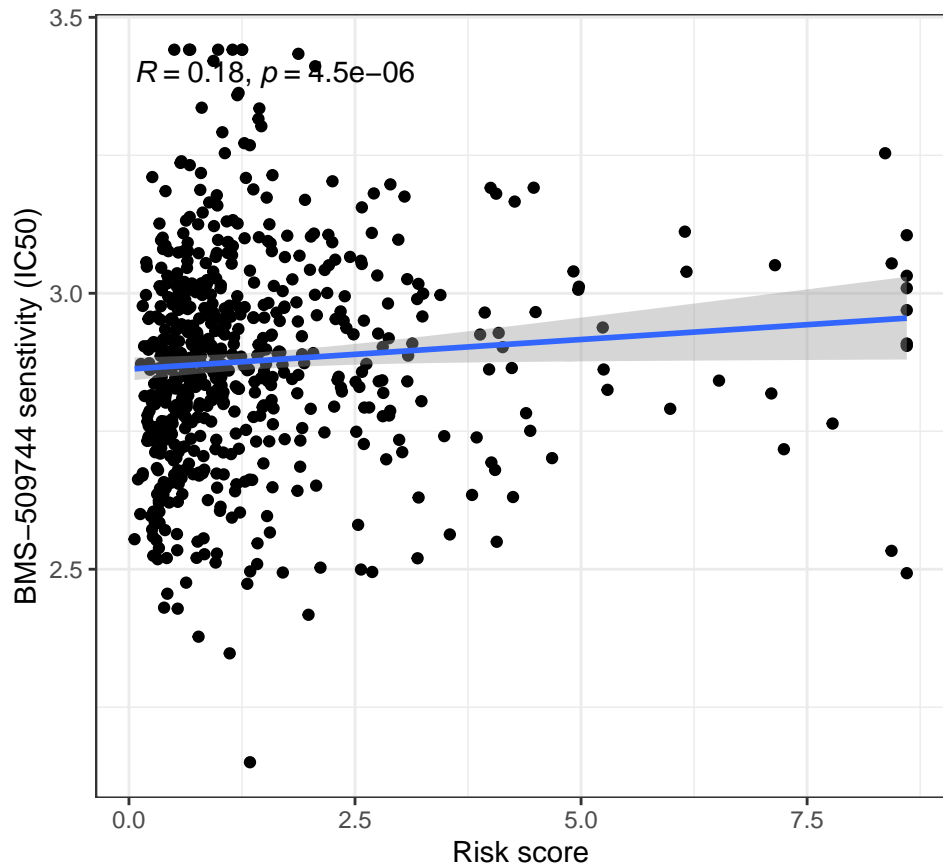

Supplement: Supplementary file 2 [file DataSheet1.ZIP › Source data for review purpose only/Source data/25.pRRophetic/Cor.BMS-509744.pdf]

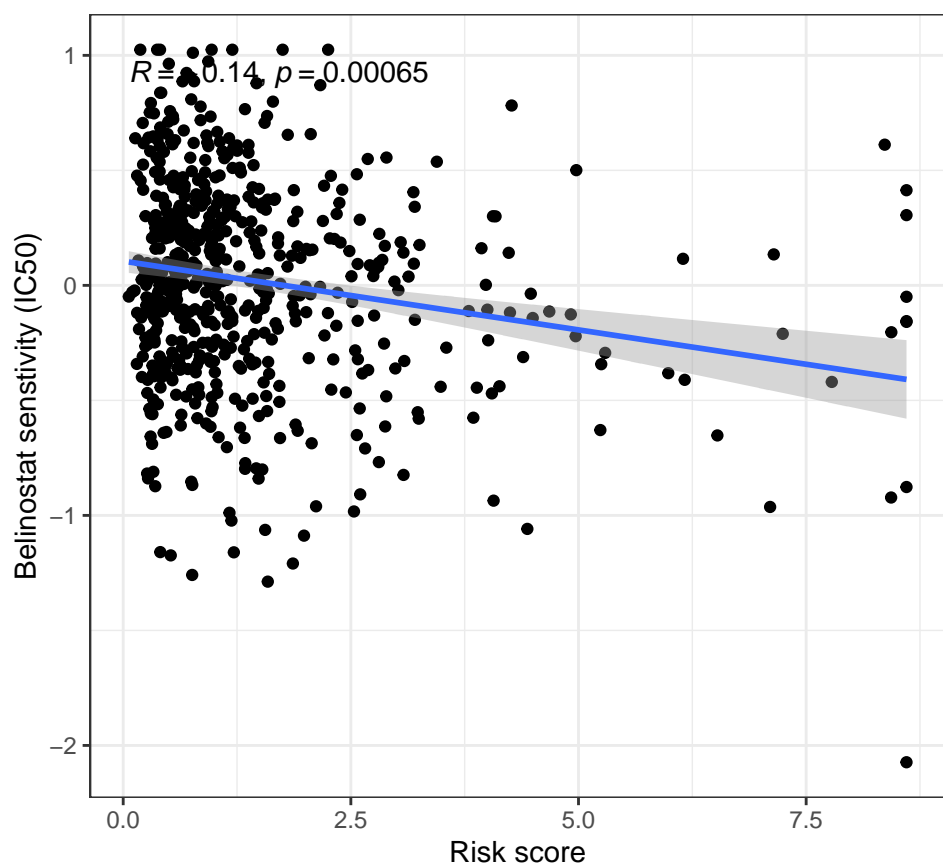

Supplement: Supplementary file 2 [file DataSheet1.ZIP › Source data for review purpose only/Source data/25.pRRophetic/Cor.Belinostat.pdf]

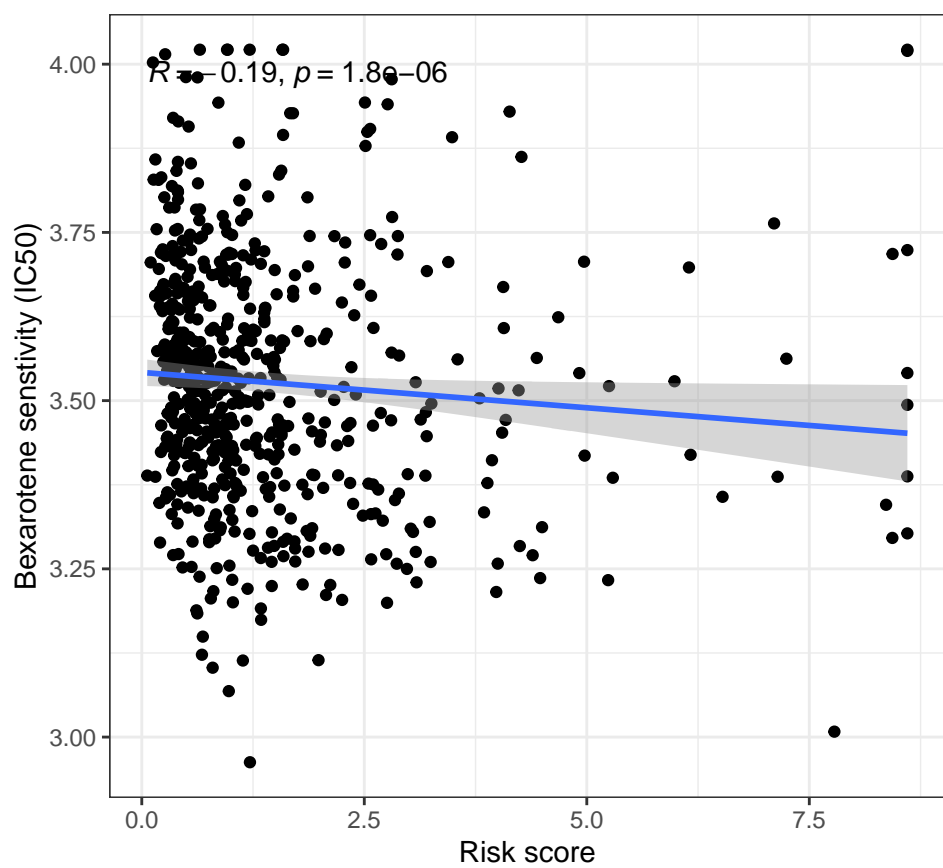

Supplement: Supplementary file 2 [file DataSheet1.ZIP › Source data for review purpose only/Source data/25.pRRophetic/Cor.Bexarotene.pdf]

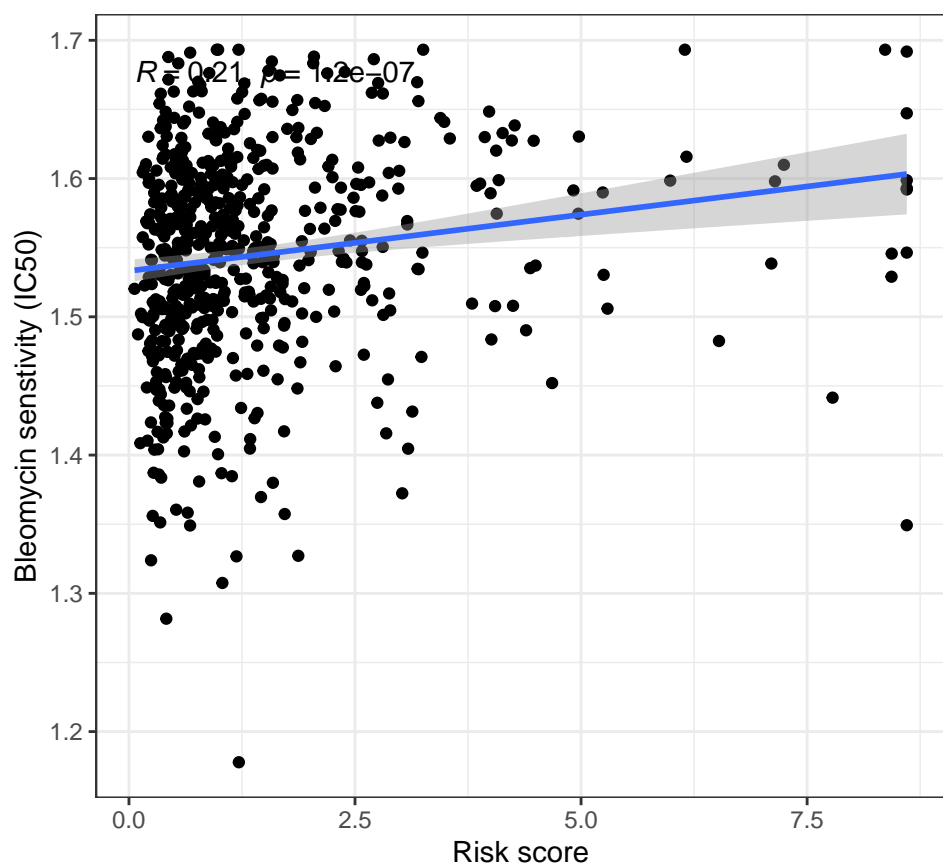

Supplement: Supplementary file 2 [file DataSheet1.ZIP › Source data for review purpose only/Source data/25.pRRophetic/Cor.Bleomycin.pdf]

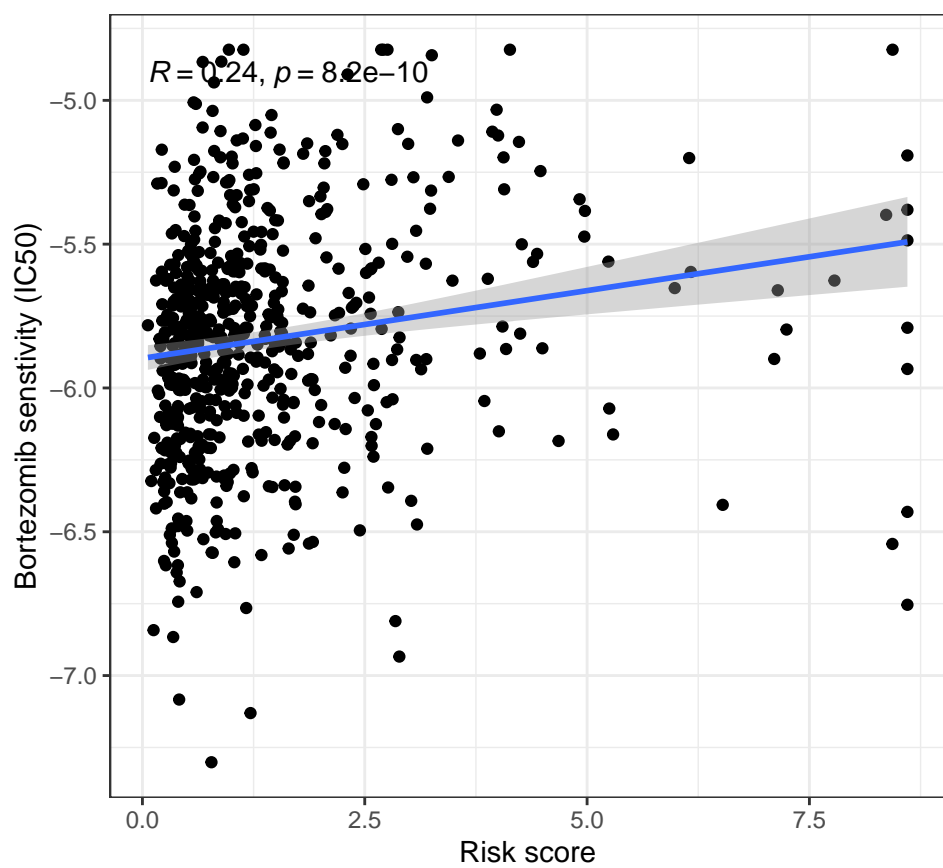

Supplement: Supplementary file 2 [file DataSheet1.ZIP › Source data for review purpose only/Source data/25.pRRophetic/Cor.Bortezomib.pdf]

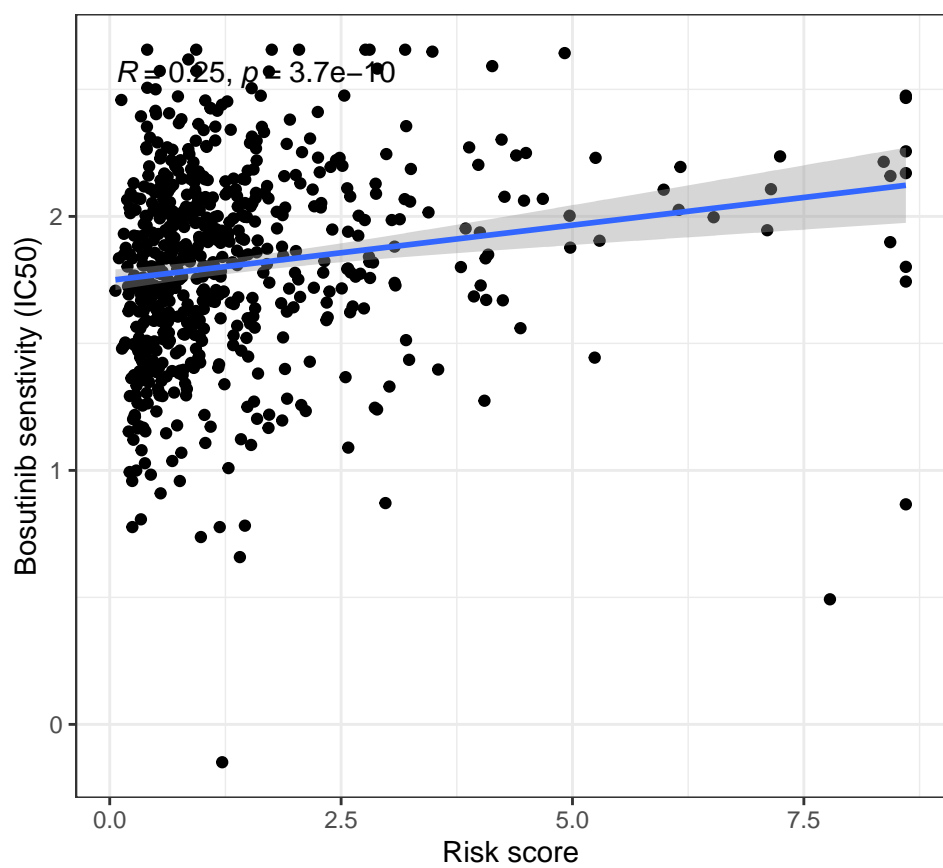

Supplement: Supplementary file 2 [file DataSheet1.ZIP › Source data for review purpose only/Source data/25.pRRophetic/Cor.Bosutinib.pdf]

CAY10603 sensitivity (IC50)

$R^2 = 0.15, p = 0.00013$

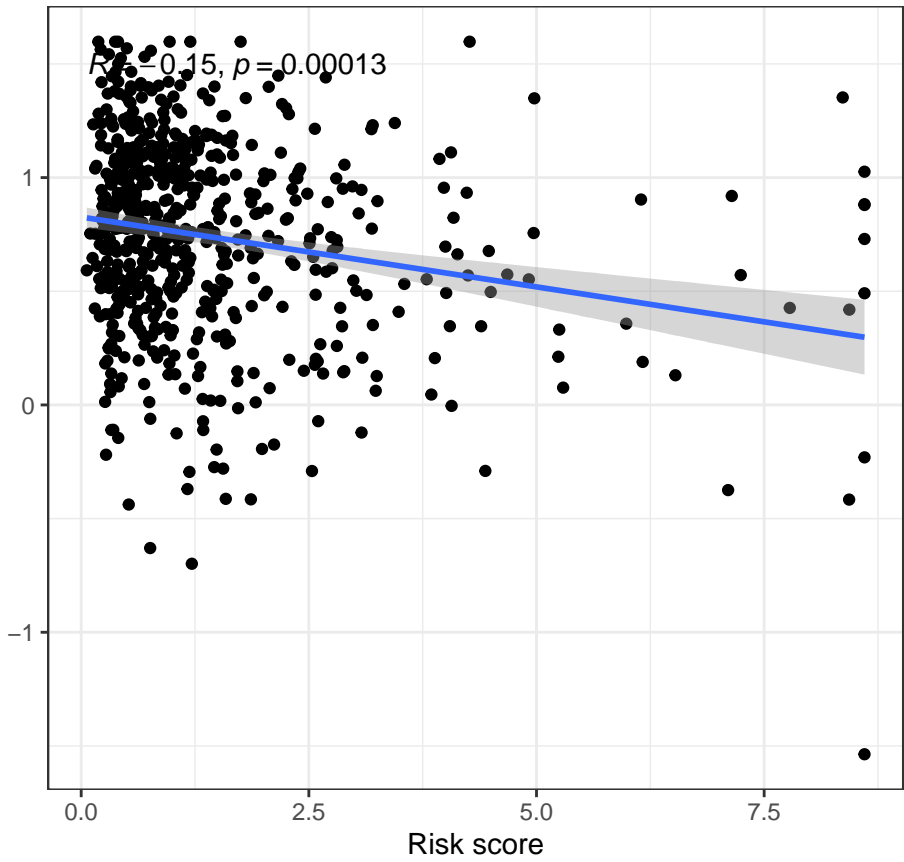

Supplement: Supplementary file 2 [file DataSheet1.ZIP › Source data for review purpose only/Source data/25.pRRophetic/Cor.CAY10603.pdf]

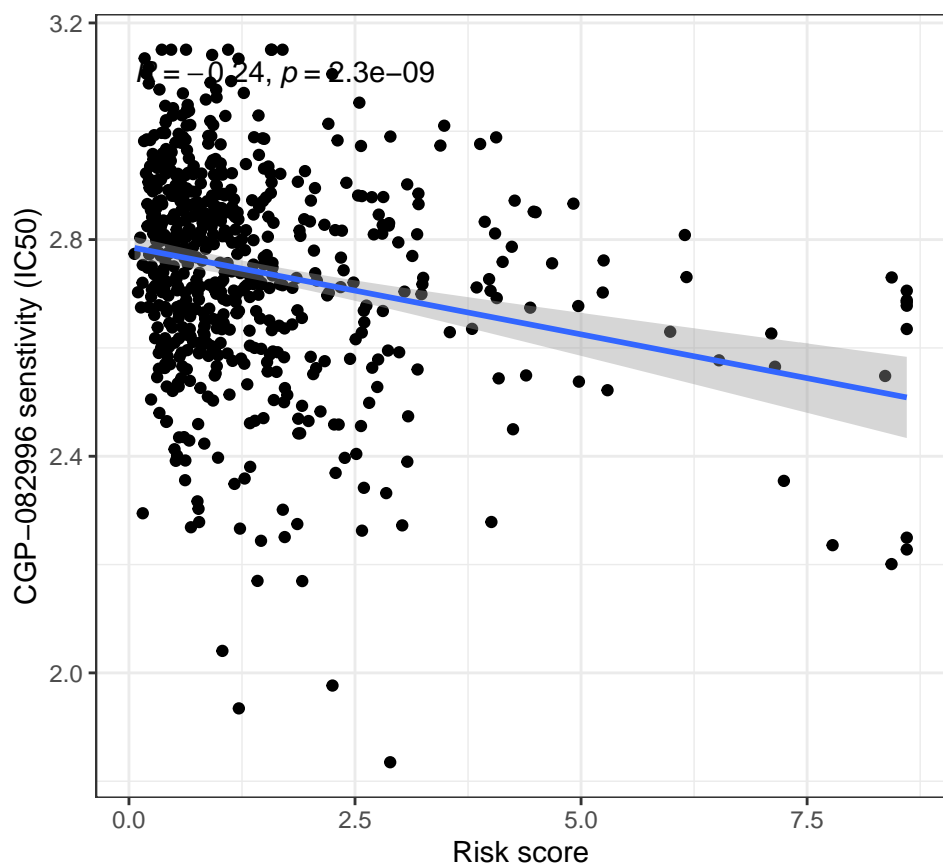

Supplement: Supplementary file 2 [file DataSheet1.ZIP › Source data for review purpose only/Source data/25.pRRophetic/Cor.CGP-082996.pdf]

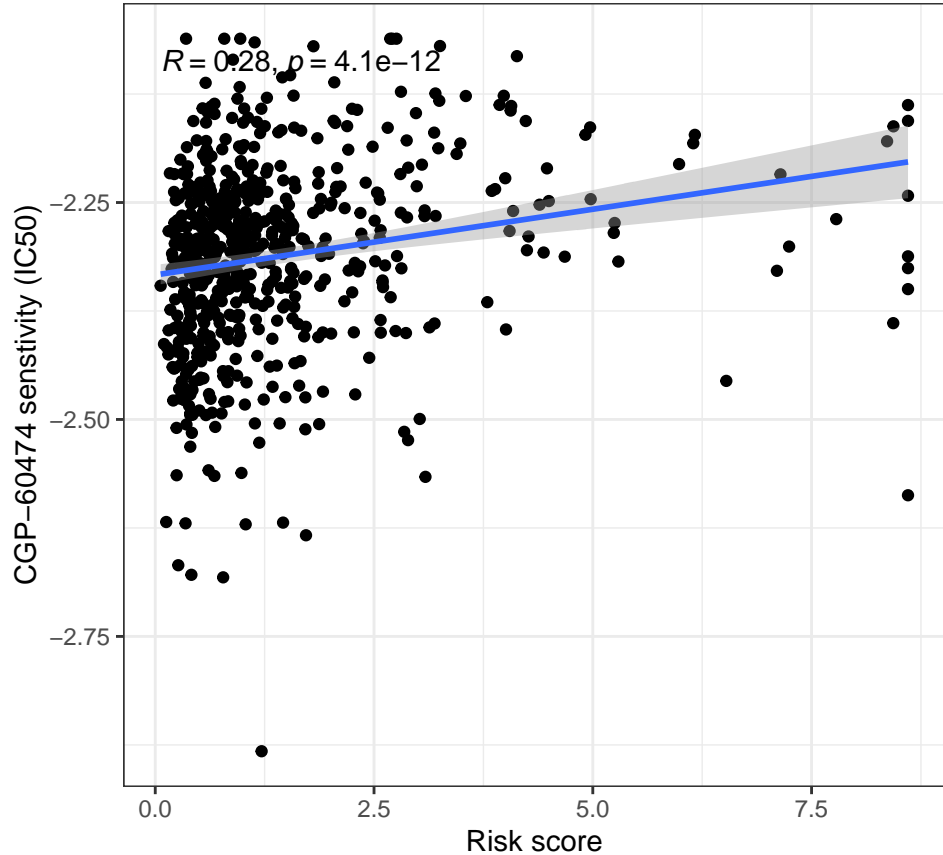

Supplement: Supplementary file 2 [file DataSheet1.ZIP › Source data for review purpose only/Source data/25.pRRophetic/Cor.CGP-60474.pdf]

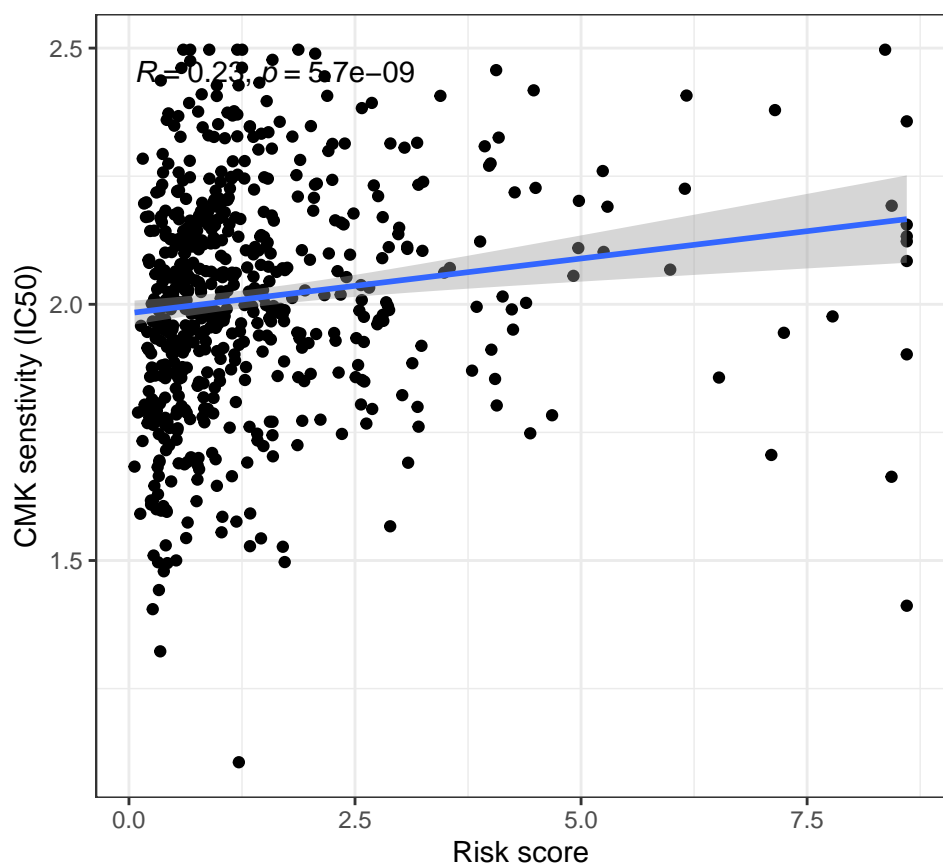

Supplement: Supplementary file 2 [file DataSheet1.ZIP › Source data for review purpose only/Source data/25.pRRophetic/Cor.CMK.pdf]

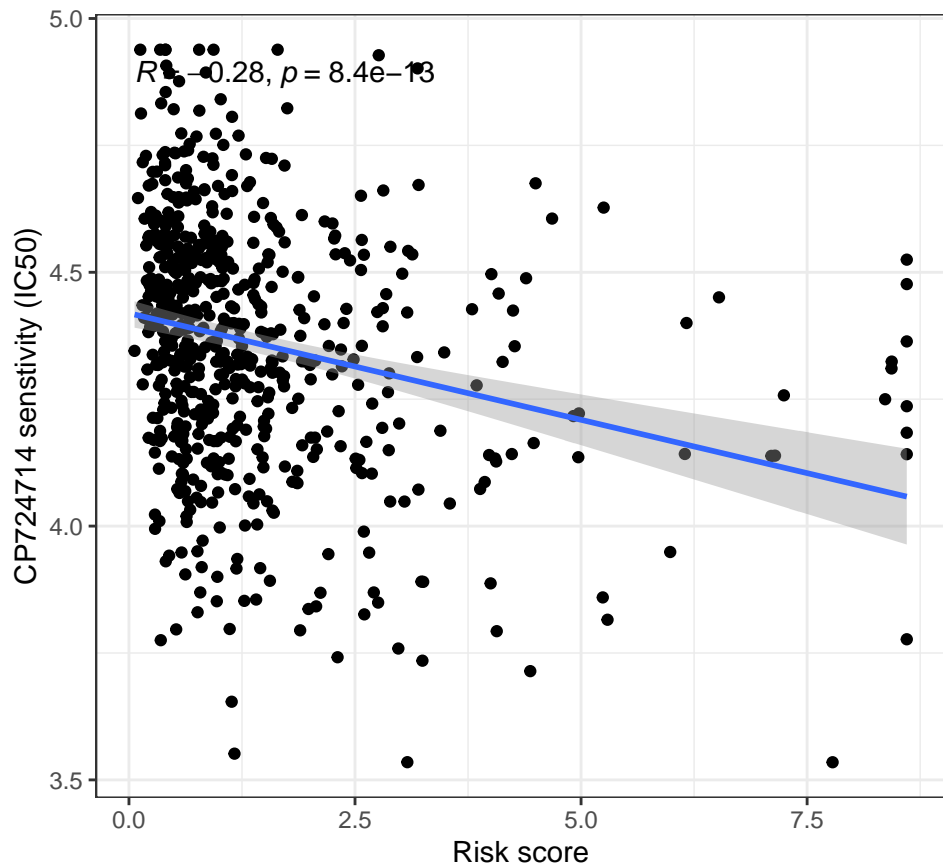

Supplement: Supplementary file 2 [file DataSheet1.ZIP › Source data for review purpose only/Source data/25.pRRophetic/Cor.CP724714.pdf]

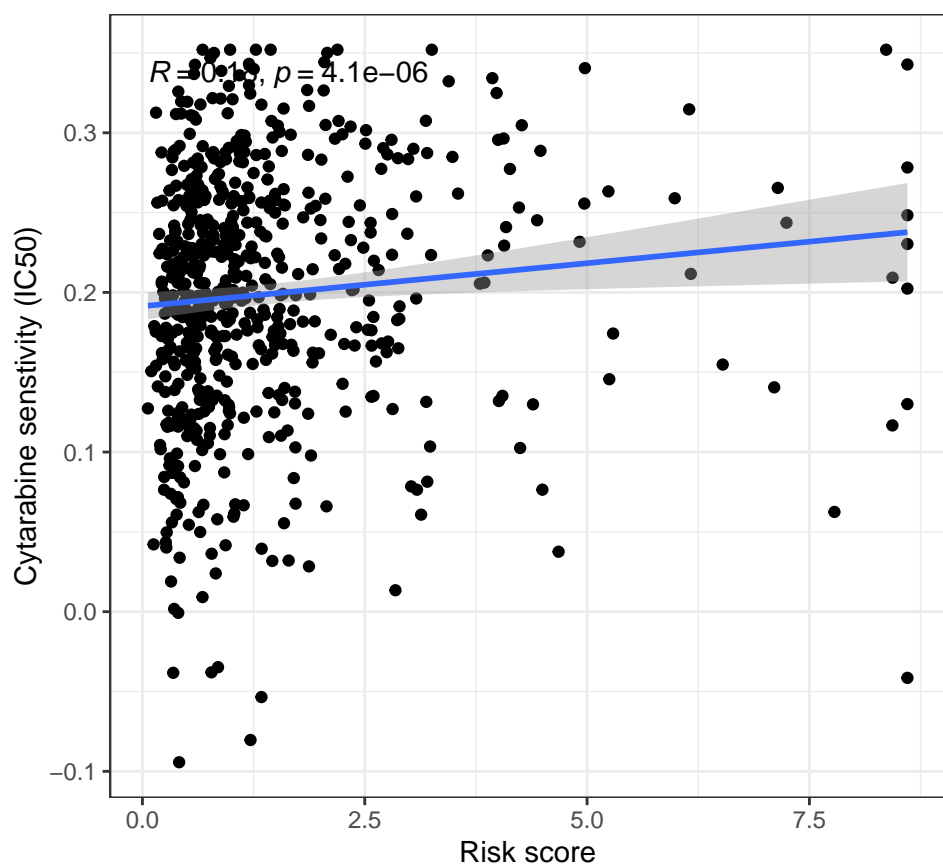

Supplement: Supplementary file 2 [file DataSheet1.ZIP › Source data for review purpose only/Source data/25.pRRophetic/Cor.Cytarabine.pdf]

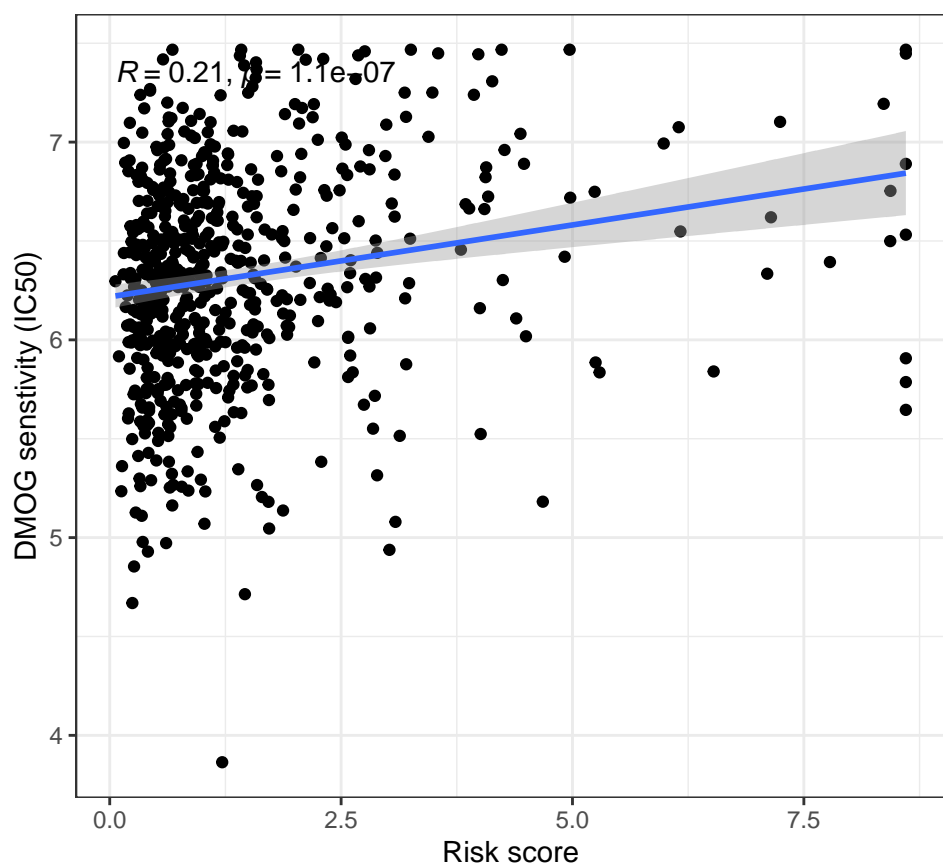

Supplement: Supplementary file 2 [file DataSheet1.ZIP › Source data for review purpose only/Source data/25.pRRophetic/Cor.DMOG.pdf]

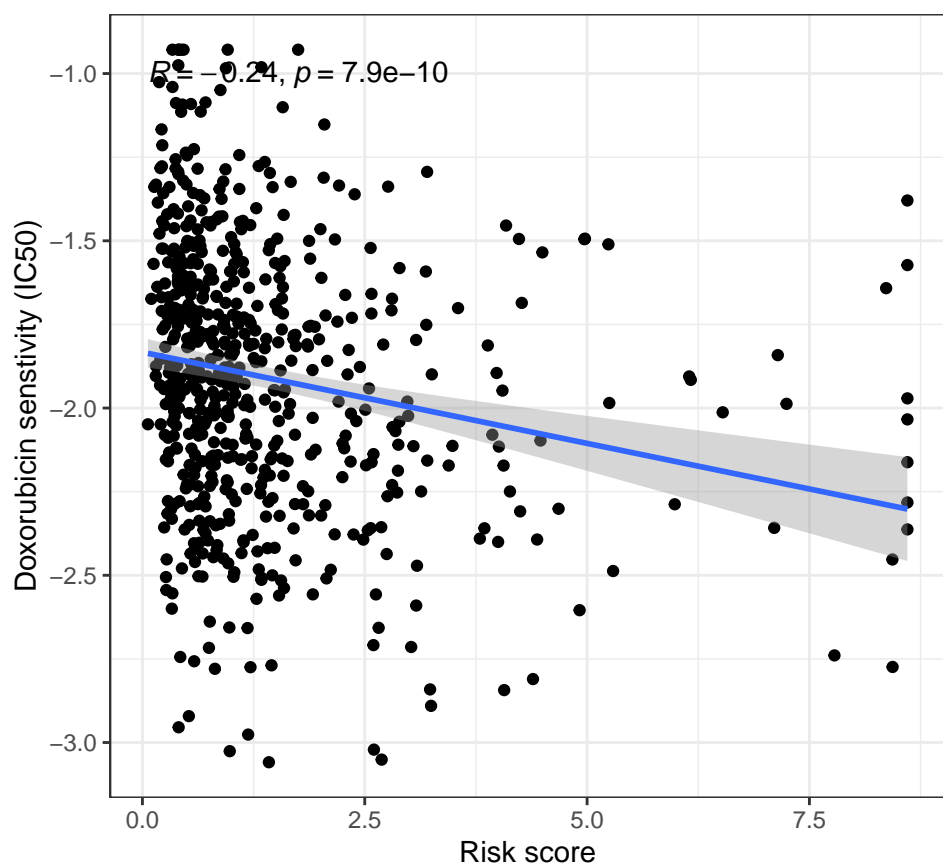

Supplement: Supplementary file 2 [file DataSheet1.ZIP › Source data for review purpose only/Source data/25.pRRophetic/Cor.Doxorubicin.pdf]

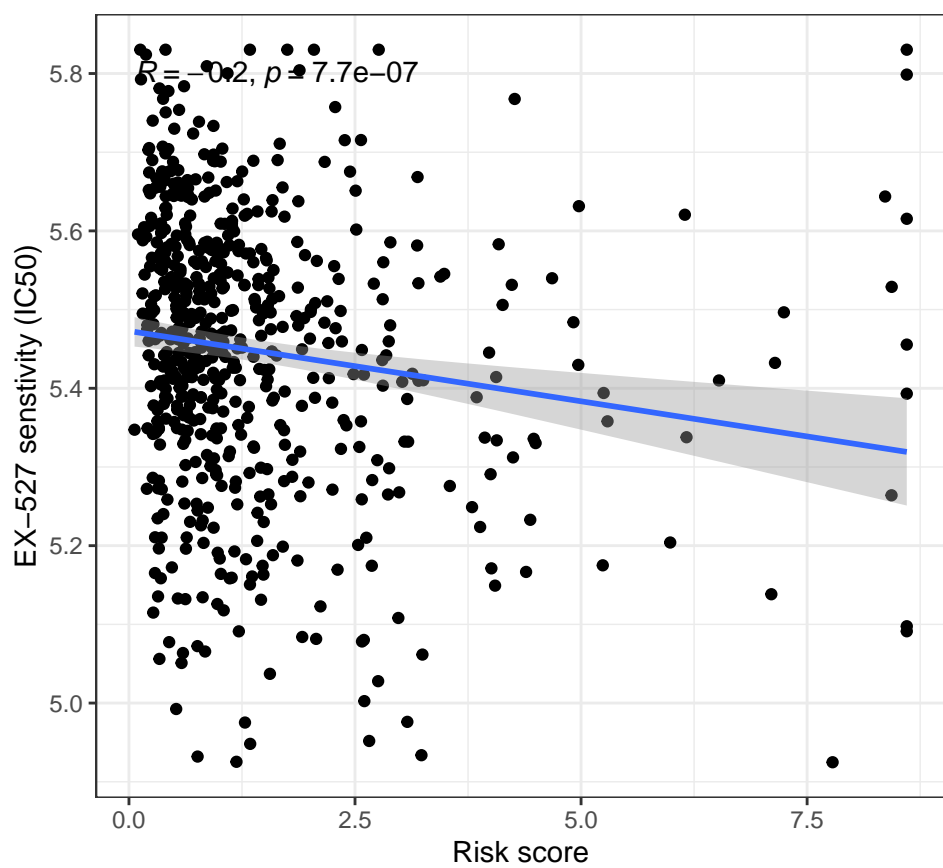

Supplement: Supplementary file 2 [file DataSheet1.ZIP › Source data for review purpose only/Source data/25.pRRophetic/Cor.EX-527.pdf]

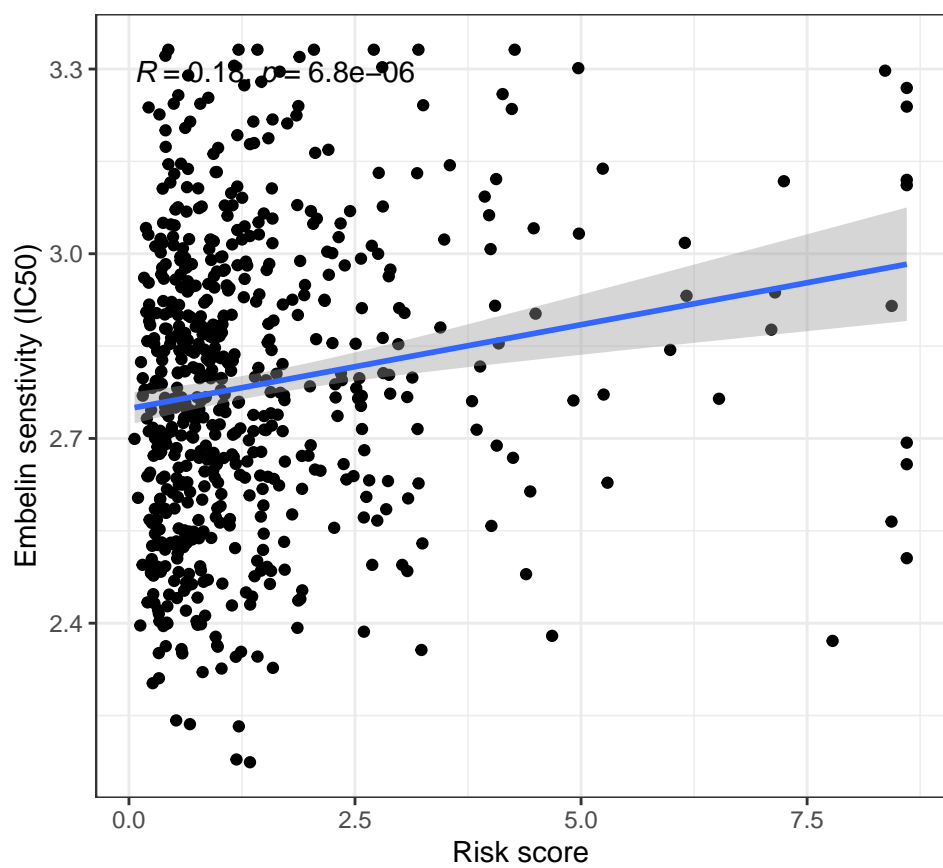

Supplement: Supplementary file 2 [file DataSheet1.ZIP › Source data for review purpose only/Source data/25.pRRophetic/Cor.Embelin.pdf]

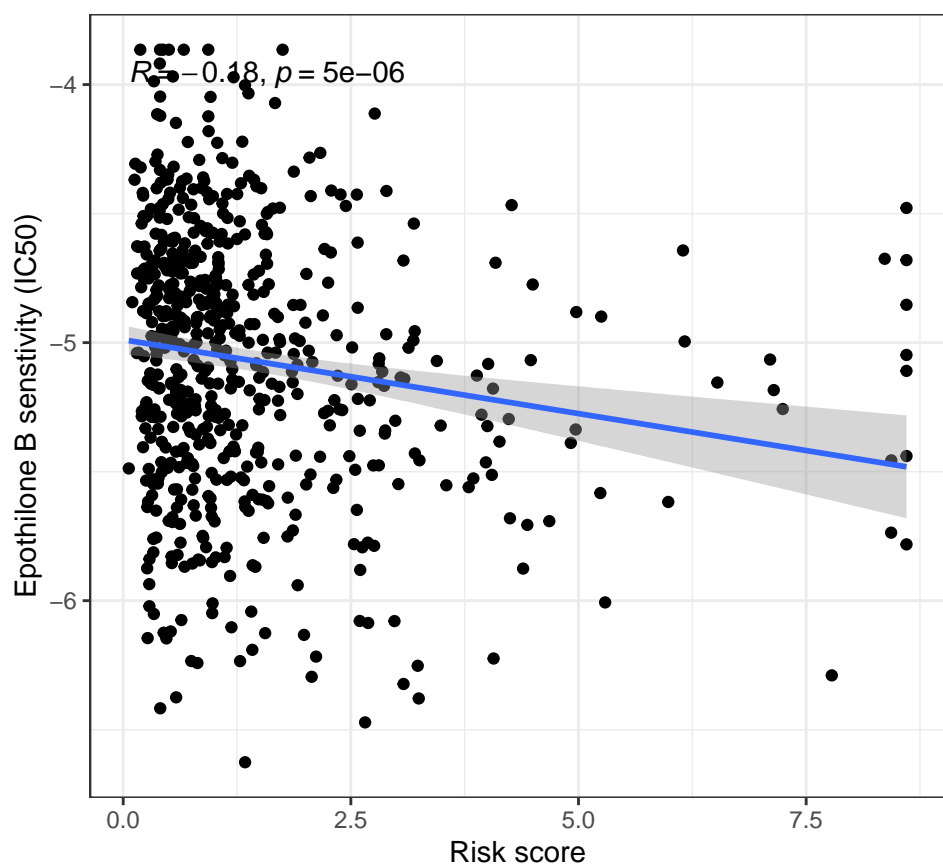

Supplement: Supplementary file 2 [file DataSheet1.ZIP › Source data for review purpose only/Source data/25.pRRophetic/Cor.Epothilone B.pdf]

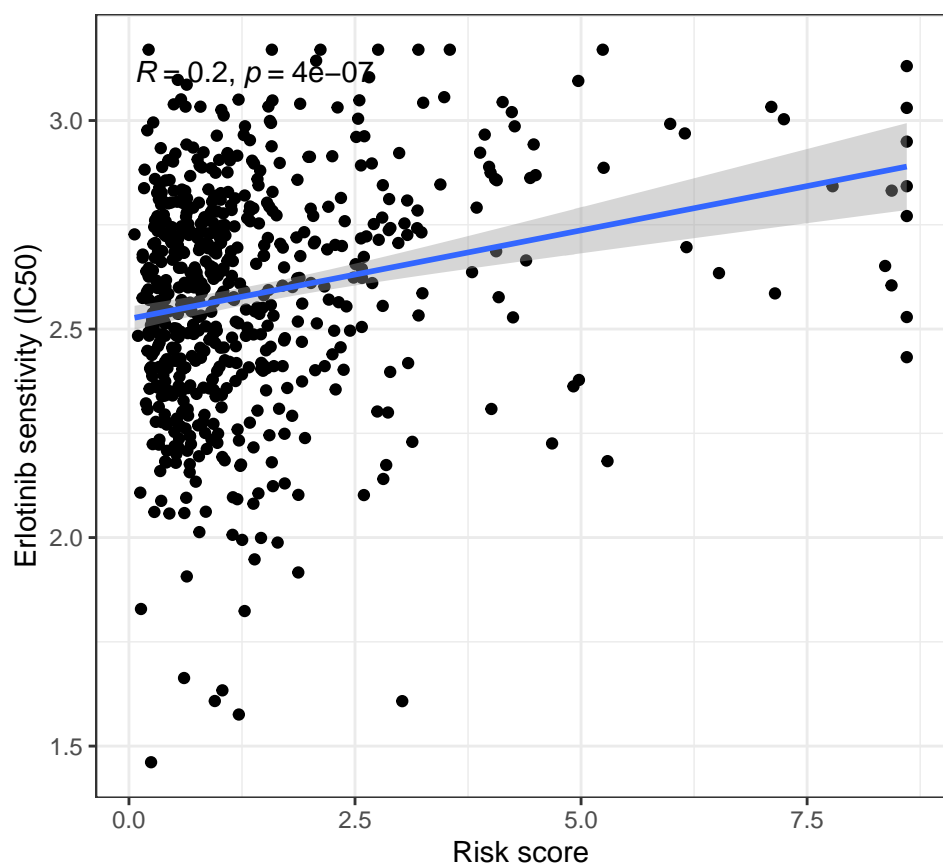

Supplement: Supplementary file 2 [file DataSheet1.ZIP › Source data for review purpose only/Source data/25.pRRophetic/Cor.Erlotinib.pdf]

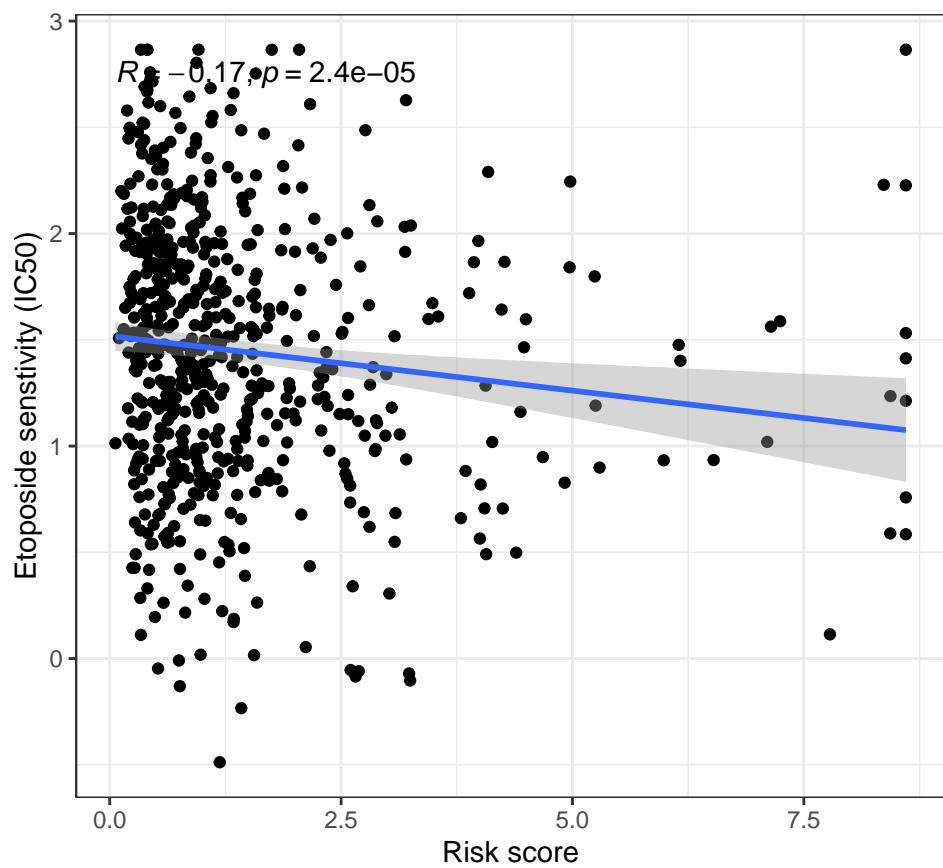

Supplement: Supplementary file 2 [file DataSheet1.ZIP › Source data for review purpose only/Source data/25.pRRophetic/Cor.Etoposide.pdf]

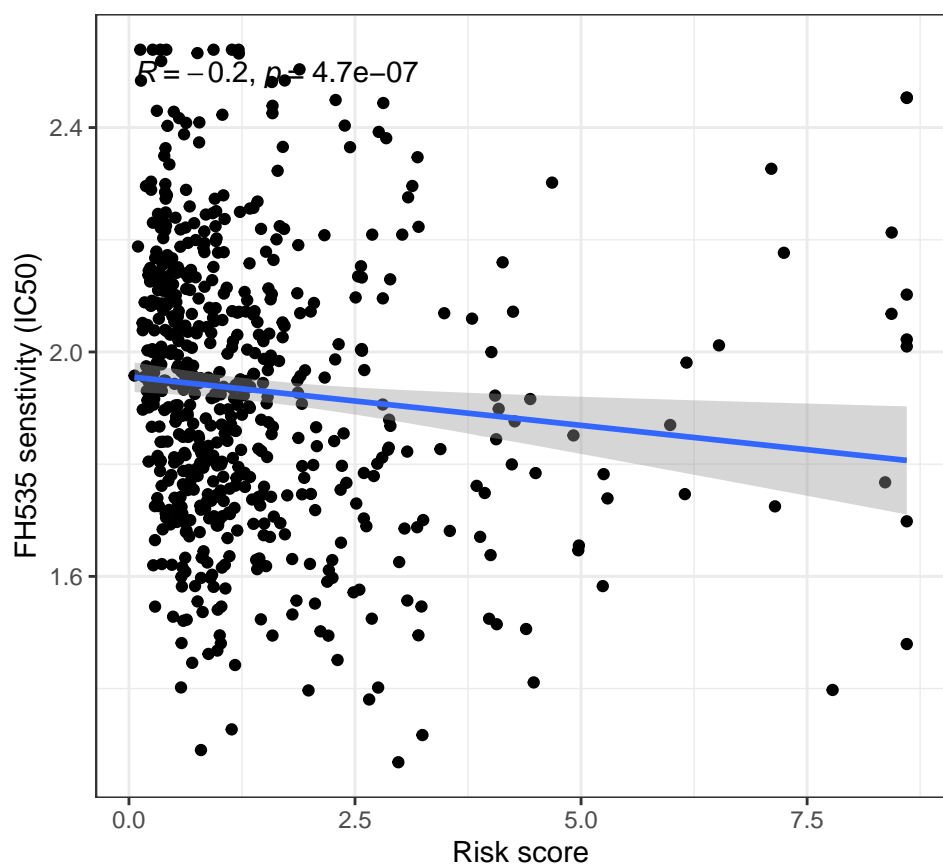

Supplement: Supplementary file 2 [file DataSheet1.ZIP › Source data for review purpose only/Source data/25.pRRophetic/Cor.FH535.pdf]

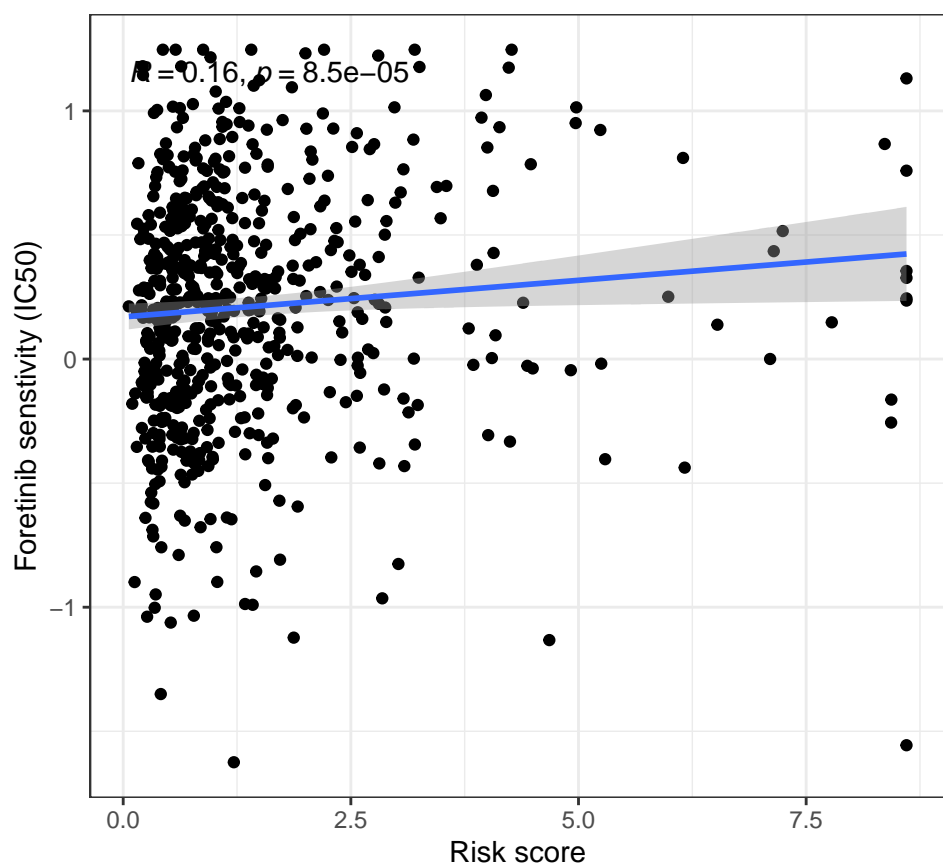

Supplement: Supplementary file 2 [file DataSheet1.ZIP › Source data for review purpose only/Source data/25.pRRophetic/Cor.Foretinib.pdf]

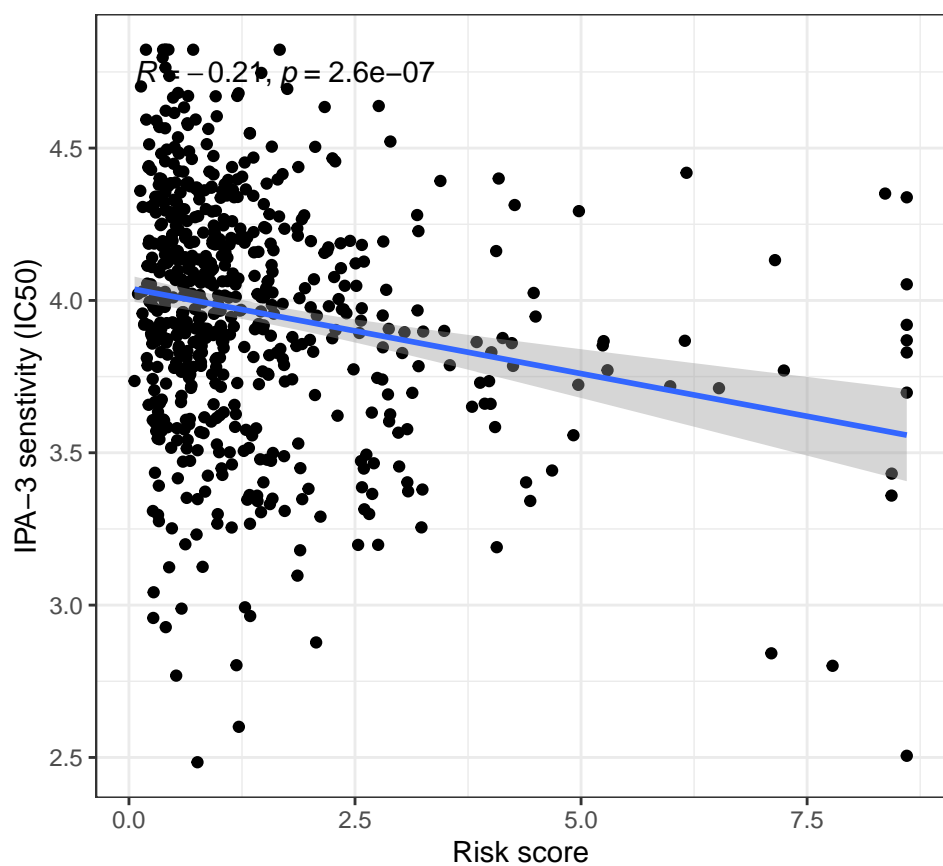

Supplement: Supplementary file 2 [file DataSheet1.ZIP › Source data for review purpose only/Source data/25.pRRophetic/Cor.IPA-3.pdf]

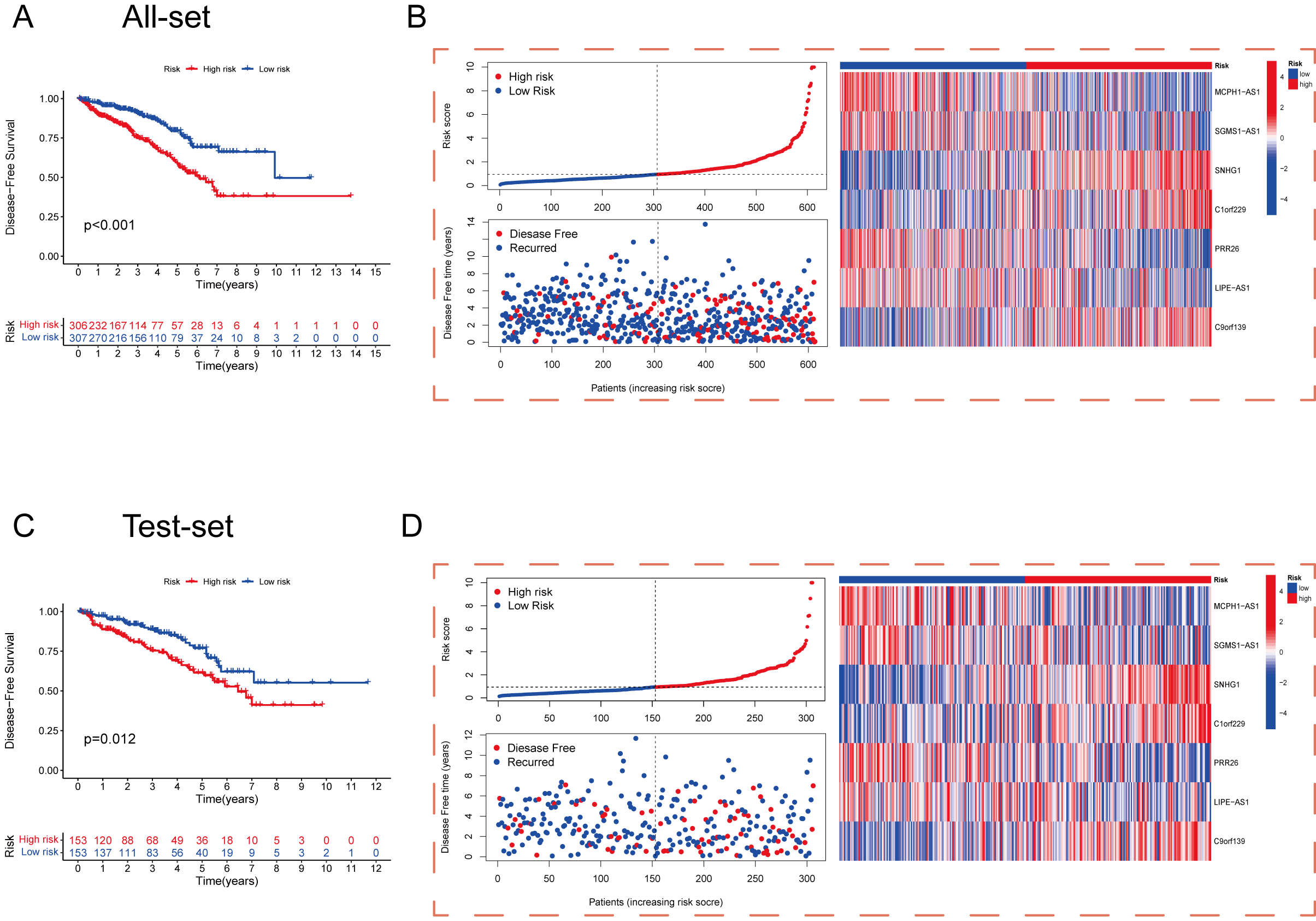

Supplement: Supplementary file 3 [file Image2.TIF]

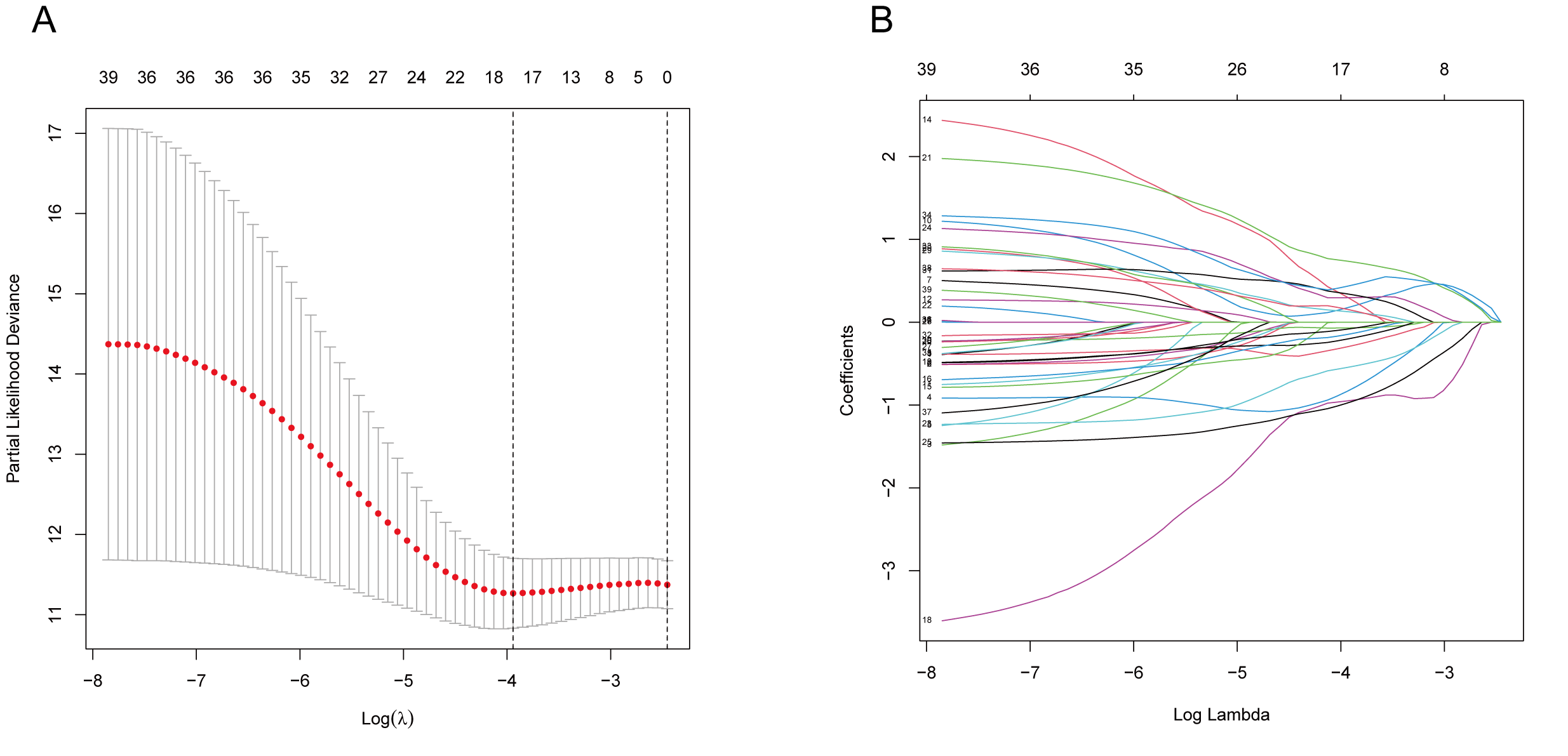

Supplement: Supplementary file 4 [file Image1.TIF]
