# Supplementary material for: Construction and validation of a novel cuproptosis-related long noncoding RNA signature for predicting the outcome of prostate cancer
Source: Front Genet. 2022 Dec 6;13:976850. doi: 10.3389/fgene.2022.976850 (PMC9763621; doi:10.3389/fgene.2022.976850)
Supplement: Supplementary file 2 [file DataSheet1.ZIP › Source data for review purpose only/Source data/04.ggalluvial/ggalluvial.pdf]

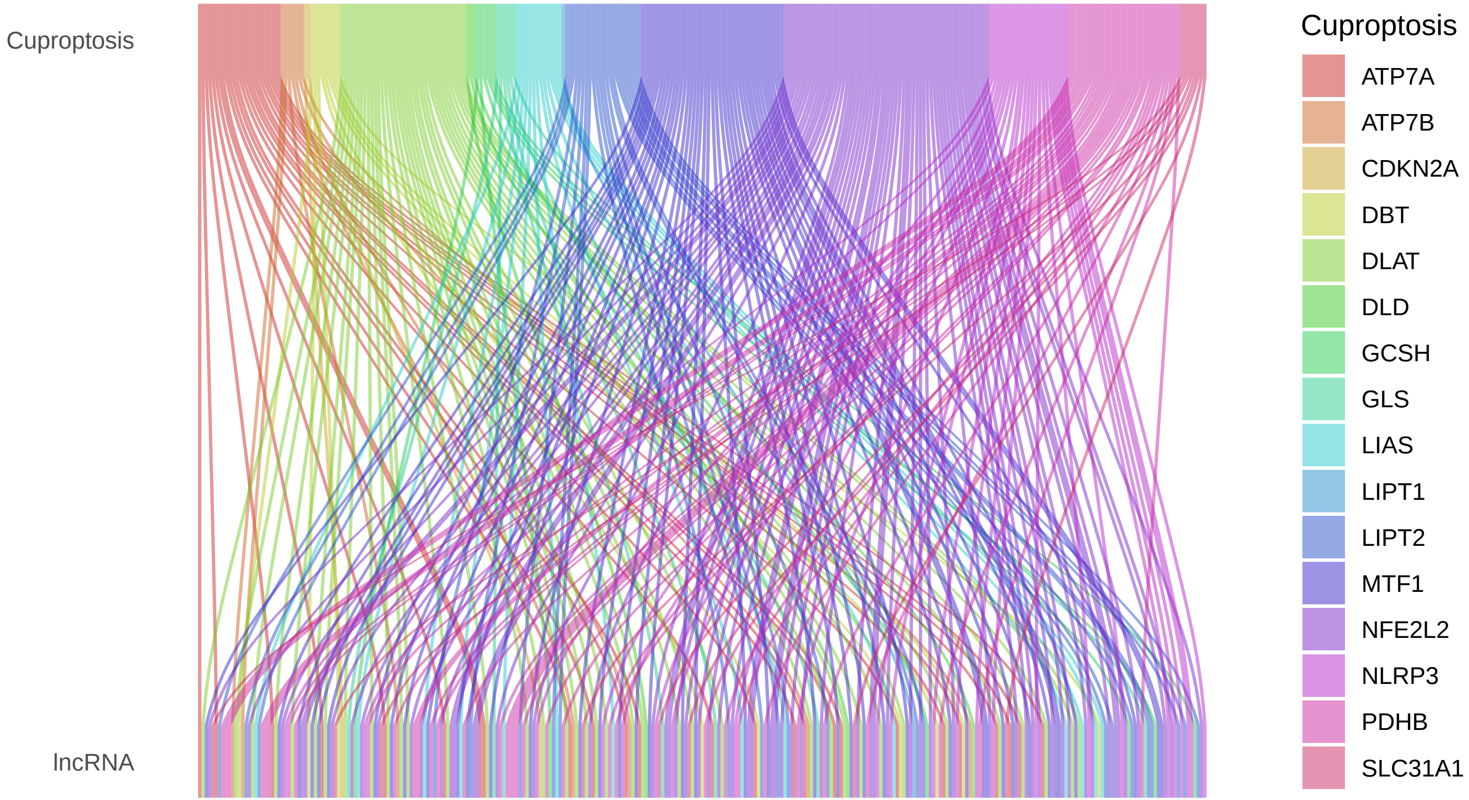

Cuproptosis
lncRNA
Cuproptosis
ATP7A
ATP7B
CDKN2A
DBT
DLAT
DLD
GCSH
GLS
LIAS
LIPT1
LIPT2
MTF1
NFE2L2
NLRP3
PDHB
SLC31A1
