# Supplementary material for: Construction and validation of a novel cuproptosis-related long noncoding RNA signature for predicting the outcome of prostate cancer
Source: Front Genet. 2022 Dec 6;13:976850. doi: 10.3389/fgene.2022.976850 (PMC9763621; doi:10.3389/fgene.2022.976850)

	pvalue	Hazard ratio
SNHG19	0.030	7.811(1.221−49.955)
CAHM	0.016	2.351(1.169−4.727)
DICER1−AS1	0.001	4.264(1.752−10.378)
MCPH1−AS1	0.017	0.208(0.057−0.757)
SNHG6	0.001	20.854(3.232−134.554)
GNG12−AS1	0.041	0.360(0.135−0.960)
TOB1−AS1	0.029	3.125(1.127−8.669)
SGMS1−AS1	0.013	0.219(0.066−0.723)
RGMB−AS1	0.040	0.601(0.371−0.976)
C8orf44	<0.001	10.094(2.825−36.071)
ATP2A1−AS1	0.048	1.892(1.005−3.562)
HCG11	0.012	0.392(0.189−0.814)
DANT2	0.032	0.366(0.146−0.917)
SNHG1	0.004	11.072(2.138−57.346)
LINC01311	0.041	2.107(1.032−4.299)
AC156455.1	0.028	0.386(0.165−0.902)
MBNL1−AS1	0.033	0.404(0.176−0.928)
OIP5−AS1	0.004	0.052(0.007−0.379)
LINC00571	0.037	0.464(0.225−0.955)
GS1−124K5.4	0.025	3.576(1.171−10.921)
C1orf229	<0.001	6.014(2.181−16.580)
MINCR	0.029	4.444(1.169−16.891)
PRR26	0.013	0.279(0.101−0.767)
LINC01138	0.001	6.923(2.171−22.074)
LIPE−AS1	0.002	0.248(0.102−0.600)
DLG5−AS1	0.029	2.520(1.100−5.774)
PAXIP1−AS1	0.009	11.332(1.838−69.858)
SRRM2−AS1	0.008	4.111(1.456−11.605)
LINC01569	0.004	5.822(1.745−19.429)
TFAP2A−AS1	0.048	2.302(1.006−5.269)
C9orf139	0.042	2.433(1.031−5.742)
ZNF529−AS1	0.023	4.956(1.243−19.749)
FENDRR	0.015	0.528(0.315−0.884)
SNHG12	<0.001	5.910(2.108−16.571)
ADAMTS9−AS2	0.014	0.396(0.189−0.832)
MAGI2−AS3	0.017	0.392(0.182−0.846)
MZF1−AS1	0.031	3.758(1.129−12.511)
LINC00623	0.026	3.365(1.158−9.779)
LINC01004	0.004	3.755(1.539−9.166)

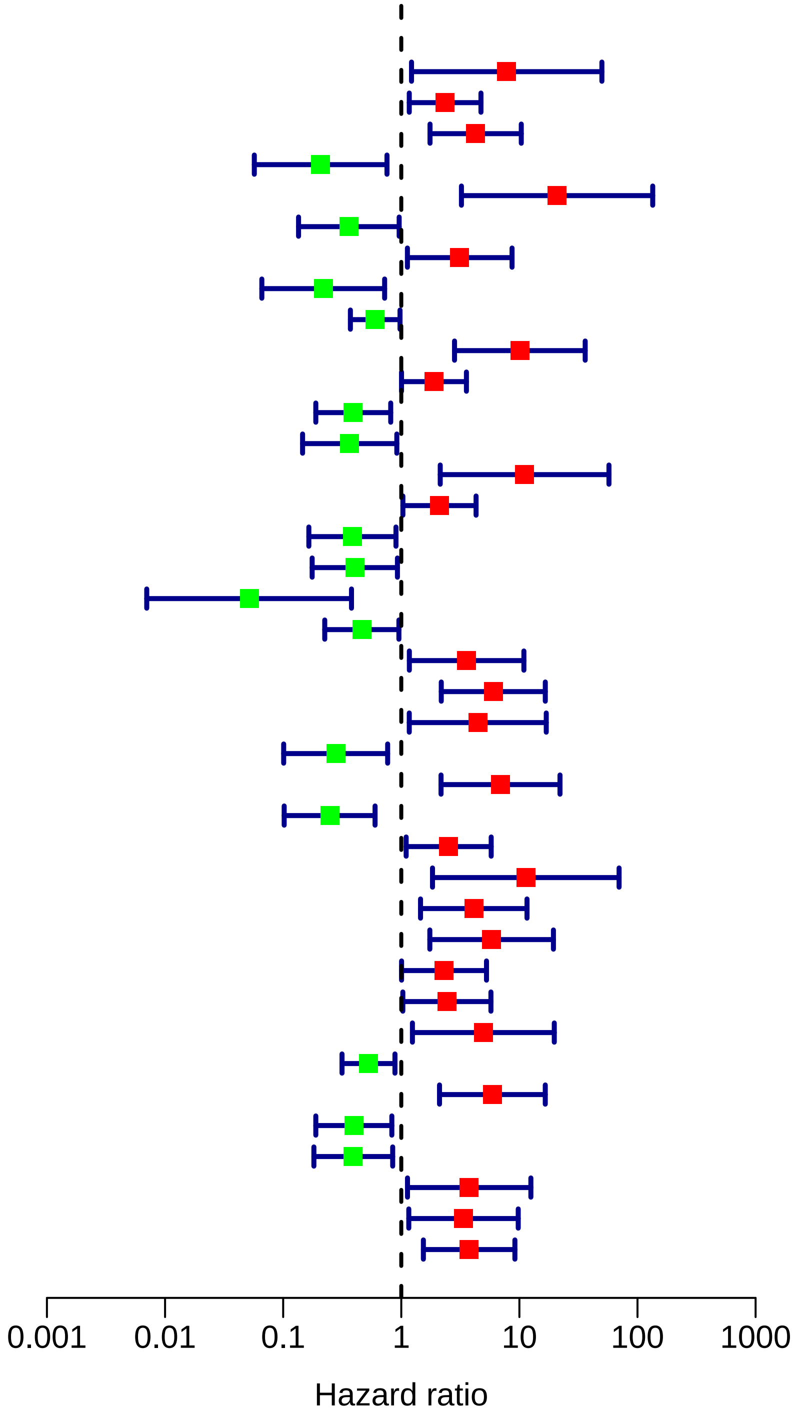

Supplement: Supplementary file 2 [file DataSheet1.ZIP › Source data for review purpose only/Source data/06.model/uni.foreast.pdf]
