# Supplementary material for: Construction and validation of a novel cuproptosis-related long noncoding RNA signature for predicting the outcome of prostate cancer
Source: Front Genet. 2022 Dec 6;13:976850. doi: 10.3389/fgene.2022.976850 (PMC9763621; doi:10.3389/fgene.2022.976850)
Supplement: Supplementary file 5 [file DataSheet3.PDF]

Supplementary Table S1.The clinical characteristics of PCa patients in the train and test set.

Characteristics	Train set	%	Test set	%	P-Value
Age					
≤57	72	23.45	92	30.07	>0.05
>57	235	76.55	214	69.63	-
Gleason					
≤7	176	57.33	187	61.11	>0.05
>7	130	42.35	119	38.89	-
unknow	1	0.33	0	0	-
Surgical Margin					
R0	182	59.28	202	66.01	>0.05
R1	109	35.5	90	29.41	-
unknow	16	5.21	14	4.58	-
T stage					
T2	112	36.48	137	44.77	0.049
T3	181	58.96	163	53.27	-
T4	10	3.26	4	1.31	-
unknow	4	1.3	2	0.65	-
N stage					
N0	222	72.31	205	66.99	>0.05
N1	47	15.31	47	15.36	-
unknow	38	12.38	54	17.65	-
M stage					
M0	251	251	256	256	>0.05
M1	9	9	6	6	-
M1b	1	1	1	1	-
unknow	46	46	43	43	-

unknow: missing data
