# Supplementary material for: Construction and validation of a novel cuproptosis-related long noncoding RNA signature for predicting the outcome of prostate cancer
Source: Front Genet. 2022 Dec 6;13:976850. doi: 10.3389/fgene.2022.976850 (PMC9763621; doi:10.3389/fgene.2022.976850)
Supplement: Supplementary file 6 [file DataSheet2.ZIP › Supplementary Material 2022 06.23.doc]

Supplementary Material

**Supplementary Figure 1.** LASSO regression model. (**A**) LASSO coefficient profiles of the CRLs correlated with DFS. (**B**) Confidence intervals for each lambda.

**Supplementary Figure 2.** Validation of risk score model. (**A**) All-set Kaplan–Meier analysis of DFS rates of high- and low-risk cohorts. (**B**) All-set risk score distribution, survival distribution, and heatmap of CRL expression for patients in high- and low-risk cohorts. (**C**) Test-set Kaplan–Meier analysis of DFS rates in high- and low-risk cohorts. (**D**) Test-set risk score distribution, survival distribution, and heatmap of CRL expression for patients in high- and low-risk cohorts.

**Supplementary Figure 3.** Calibration curves for 1-, 3-, and 5-year DFS predicted by nomograms.
